# A consistent vigor network in Parkinson's disease: a potential target for brain stimulation effects

**DOI:** 10.3389/fnhum.2026.1928519

**Published:** 2026-09-30

**Authors:** Zahra Kavian, Sepideh Hajipour Sardouie, Emad Arasteh, Maryam S. Mirian, Varsha Sreenivasan, Milad Yazdani, Martin J. McKeown

**Affiliations:** 1 Pacific Parkinson's Research Center, Djavad Mowafaghian Center for Brain Health, University of British Columbia, Vancouver, BC, Canada; 2 Department of Electrical and Computer Engineering, University of British Columbia, Vancouver, BC, Canada; 3 Faculty of Medicine (Neurology), University of British Columbia, Vancouver, BC, Canada

**Keywords:** dopaminergic medication, fMRI, functional connectivity, galvanic vestibular stimulation, motor vigor, Parkinson's disease, reaction-time variability

## Abstract

**Introduction:**

In motor tasks with repetitive trials, motor vigor varies substantially from trial to trial in people with Parkinson's disease (PD). A key question is whether neural variability reflects behavioral variability or whether additional variability emerges as the neural signal is translated into movement. We aimed to identify a vigor-related neural network that could provide a candidate target for brain stimulation.

**Methods:**

We analyzed individual-trial fMRI data from 18 participants with PD who performed an overlearned ballistic hand-squeeze task in OFF- and ON-medication sessions while receiving subthreshold galvanic vestibular stimulation (GVS) with different stimulus waveforms. Single-trial fMRI responses were estimated using GLMsingle and entered into a constrained optimisation framework designed to identify a network that was task-active, encoded behavior, showed low trial-to-trial variability, and was spatially coherent.

**Results:**

The analysis identified a distributed vigor network whose activity remained related to behavioral vigor while showing lower variability than behavior itself. The network included motor, striatal, cerebellar, thalamic, parietal, temporal-limbic, and orbitofrontal regions and showed limited overlap with the standard task-activation map. GVS waveforms associated with faster responses were accompanied by corresponding changes in vigor-network activity, but not in the broader task-activation network. Dopaminergic medication was also associated with stronger local functional connectivity within the vigor network.

**Discussion:**

These findings suggest that movement variability in PD may arise partly when a relatively stable central vigor-related command is translated downstream into movement. The identified network may therefore provide a potential target for neuromodulation approaches aimed at increasing motor vigor in people with PD. Independent validation and future causal testing are needed.

## 1 Introduction

Motor vigor refers to how quickly and energetically an action is initiated and carried out. It is not a fixed property of the movement itself: the same person can perform the same action faster or slower across trials, and different people often express different levels of speed and force during the same task (Berret et al., 2018). fMRI studies have suggested that movement speed is related to a distributed set of cortical and subcortical regions, including motor and premotor cortices, supplementary motor area, basal ganglia, thalamus, parietal cortex, and cerebellum (Shirinbayan et al., 2019). Consistent with this, direct electrocorticographic recordings have demonstrated that cortical activity encodes both the direction and velocity of human arm movements (Anderson et al., 2012), while studies of the basal ganglia have shown that individual nuclei contribute to the regulation of movement force (Spraker et al., 2007). Collectively, these findings suggest that motor vigor is shaped by coordinated activity distributed across cortical and subcortical motor circuits.

Dopamine is central to motor vigor. Basal-ganglia output can change movement speed and amplitude without necessarily changing which action is selected (Turner and Desmurget, 2010), and striatal pathway manipulations can speed up or slow down movement in a dopamine-dependent manner (Yttri and Dudman, 2016). Reward and motivational value also influence motor vigor: actions associated with higher value are often initiated faster and executed with greater speed (Kawagoe et al., 1998; Reppert et al., 2015; Summerside et al., 2018). Parkinson's disease (PD), which is characterized by dopaminergic loss in basal-ganglia circuits, is therefore a useful model for asking how the dopaminergic state affects the expression of motor vigor.

Dopamine deficiency in PD is closely linked to bradykinesia, i.e., movements that are slower and typically smaller in amplitude. However, it is not simply that PD patients cannot move quickly. When speed is strongly encouraged, patients with PD can sometimes produce rapid movements (Mazzoni et al., 2007). This points to a different problem: PD may make it harder to set and maintain the appropriate level of motor vigor from one trial to the next. Behavioral studies report larger short-term reaction-time fluctuations in PD (MacDonald et al., 2025), and earlier work found increased endpoint variability that could be partly reduced when participants slowed down or relied on visual guidance (Sheridan and Flowers, 1990). Motor output is also less stable: motor-unit discharge can become more variable in mild-to-moderate PD even when mean discharge rates are relatively preserved (Wilson et al., 2020). Dopamine loss may therefore affect not only average movement features, but also how consistently those features are expressed (Lee et al., 2015a).

A key difficulty in interpreting motor vigor variability is that there can be several possible sources. Some movement variability is useful, helping the motor system explore actions, adapt after errors, and learn from reward; in particular, task-relevant movement variability can predict faster motor learning (Pekny et al., 2015; Wu et al., 2014). Some variability reflects the neural state before movement; preparatory activity in motor and premotor cortex can predict the speed of the upcoming reach (Churchland et al., 2006). Another source is motor noise (Faisal et al., 2008), especially signal-dependent noise, where larger motor commands generate greater variability and make fast or forceful movements harder to control accurately (Harris and Wolpert, 1998). In PD, such downstream noise may be behaviourally costly: signal-dependent noise has been linked to effort costs in the more affected arm, and reducing this noise increased patients' willingness to use that arm (Salimpour et al., 2015). More generally, noisy motor output can make the outcome of an action less predictable. When similar commands produce inconsistent outcomes, the motor system receives a less reliable teaching signal, which can impair learning (Therrien et al., 2016).

Since these sources occur together, behavior alone cannot distinguish whether variable motor vigor reflects instability of a central neural command or noise added later in the motor pathway. This distinction matters for PD: if the central motor vigor command itself is unstable, then bradykinesia and inconsistency may reflect impaired neural coding. If a relatively stable central neural command still tracks trial-wise behavior while behavioral output fluctuates, then part of the instability may arise downstream of that command or during its translation into movement. Distinguishing these possibilities requires measuring the neural activity. Trial-wise fMRI provides a way to estimate neural responses for individual trials (Mumford et al., 2012; Prince et al., 2022) and relating neural variability to behavior (Garrett et al., 2013; Haar et al., 2017).

Defining a trial-wise fMRI network related to motor vigor would provide a new network for evaluating the effects of neuromodulatory techniques that may influence motor circuits in PD. Galvanic vestibular stimulation (GVS), whereby weak electrical currents are applied through electrodes placed over the mastoid processes, thereby perturbing vestibular afferent activity, is one such perturbation (Fitzpatrick and Day, 2004; Kwan et al., 2019). Because vestibular signals contribute to posture, balance, and motor control, GVS has been used to test whether vestibular perturbation can influence motor behavior in PD (Lee et al., 2015b, 2021). GVS has also been shown to alter functional connectivity in PD, including connectivity involving the pedunculopontine nucleus and larger brain subnetworks (Cai et al., 2018; Liu et al., 2021). In PD, GVS effects appear to depend on the stimulation waveform and medication state. For example, multisine GVS shortened response time in an OFF-medication simple response-time task, but not in the ON-medication state (Lee et al., 2021). Separately, fMRI work showed that noisy and sinusoidal GVS increased the overall magnitude of deficient pedunculopontine nucleus connectivity in PD in a stimulus-dependent manner (Cai et al., 2018). These findings suggest that vestibular stimulation can interact with motor-related circuits in PD, especially when the dopaminergic state is altered. However, they do not identify which brain system should be used to evaluate GVS effects on motor vigor. Most prior work has focused on predefined vestibular, gait, brainstem, or task-related targets, whereas motor vigor is likely expressed through a distributed network linking motor, basal-ganglia, cerebellar, and motivational systems. A data-driven vigor network could therefore provide a more appropriate target for determining the effects of different GVS waveforms to augment motor vigor.

We identified and internally evaluated a data-driven vigor network in PD. The analyses are organized into two stages: first, we use trial-wise fMRI and behavioral data from a ballistic hand-squeeze task in participants with PD, scanned OFF and ON dopaminergic medication while receiving different GVS stimuli, to define the network. This stage uses a constrained optimisation problem to ensure task engagement, behavioral linkage, spatial coherence, and low consecutive-trial variability, and then tests whether the resulting network is reproducible, less variable than RT, and anatomically distinct from standard task activation. Second, once the vigor network is defined, we interrogate it to determine the effects of GVS and medication. GVS is used to test whether external vestibular stimulation alters the temporal profile of the vigor network, or functional connectivity within the network, relative to sham stimulation and to the task-activation map. We then determine whether the functional organization of the network changes with dopaminergic state by assessing people OFF and ON dopaminergic medication.

## 2 Materials and methods

### 2.1 Participants and medication-state design

Eighteen participants with Parkinson's disease were included in the final analysis: 4 female and 14 male participants, with a mean age of 67.22 ± 7.03 years, range 55--82 years. Fourteen participants were right-handed, and four were left-handed. Participants performed the task with their dominant hand, except for one right-handed participant who used the left hand because of severe tremor in the dominant hand. Data from one participant's first session, the OFF-medication session, were excluded because one recording run was excessively noisy. Exclusion criteria included prior neurosurgical treatment for PD, other neurological or major medical conditions, dementia severe enough to preclude informed consent, severe levodopa-induced dyskinesia incompatible with MRI, and standard contraindications to MRI. Clinical severity was assessed using the MDS-UPDRS Part III (Goetz et al., 2008); medication-state totals are reported in Table 1. Further participant characteristics and MDS-UPDRS domain scores are reported in Supplementary Tables S1, S2. All participants provided written informed consent, and the study was approved by the Clinical Research Ethics Board at the University of British Columbia.

**Table 1:** Clinical motor severity by medication state.

Measure	OFF medication	ON medication	ON–OFF
MDS-UPDRS Part III total, mean ± SD	37.3 ± 17.3	28.0 ± 15.0	−9.3 ± 4.2
MDS-UPDRS Part III total, median [range]	35.0 [10, 75]	22.0 [7, 61]	−9.0 [−17, −3]
Relative reduction from OFF, %, mean ± SD	—	26.6 ± 9.1	—
Minutes since last dose, median [range]	931.5 [720, 2,286]	71.0 [36, 102]	—
H&Y stage, median [range]	2 [1, 4]	2 [0, 3]	—
Dyskinesias present, *n/N*	1/18	3/18	—
Dyskinesias interfered with ratings, *n/N*	1/18	0/18	—

MDS-UPDRS Part III ratings were available for all 18 participants. Values are reported as mean ± SD, median [range], or *n/N*.

Participants completed two medication sessions on the same day: OFF-medication and ON-medication. They were instructed to stop dopaminergic medication at least 12 h before recording. The session order was fixed across participants: the OFF-medication session was always completed first, after which participants took their dopaminergic medication and completed the ON-medication session approximately 1 h later. Because OFF sessions always preceded ON sessions, medication effects could be partly confounded with practice, fatigue, time-on-task, or scanner drift.

### 2.2 Behavioral task and GVS protocol

During fMRI, participants performed a ballistic hand-squeeze task implemented in MATLAB with Psychtoolbox (Brainard, 1997) while lying supine in the scanner and viewing the task through a mirror display (Figure 1A). On each trial, a visual cue indicating reward value was presented for 2 s, followed by a variable delay of 2–3.5 s. A moving balloon then appeared as the Go signal. Participants were instructed to squeeze a mechanical bulb as quickly and forcefully as possible when the Go signal appeared, and successful responses were followed by a visual burst of the balloon. Reaction time (RT), defined as the interval between Go-signal onset and squeeze initiation, was used as the primary trial-wise index of motor vigor because it was the most robust behavioral measure in this dataset.

**Figure 1:**
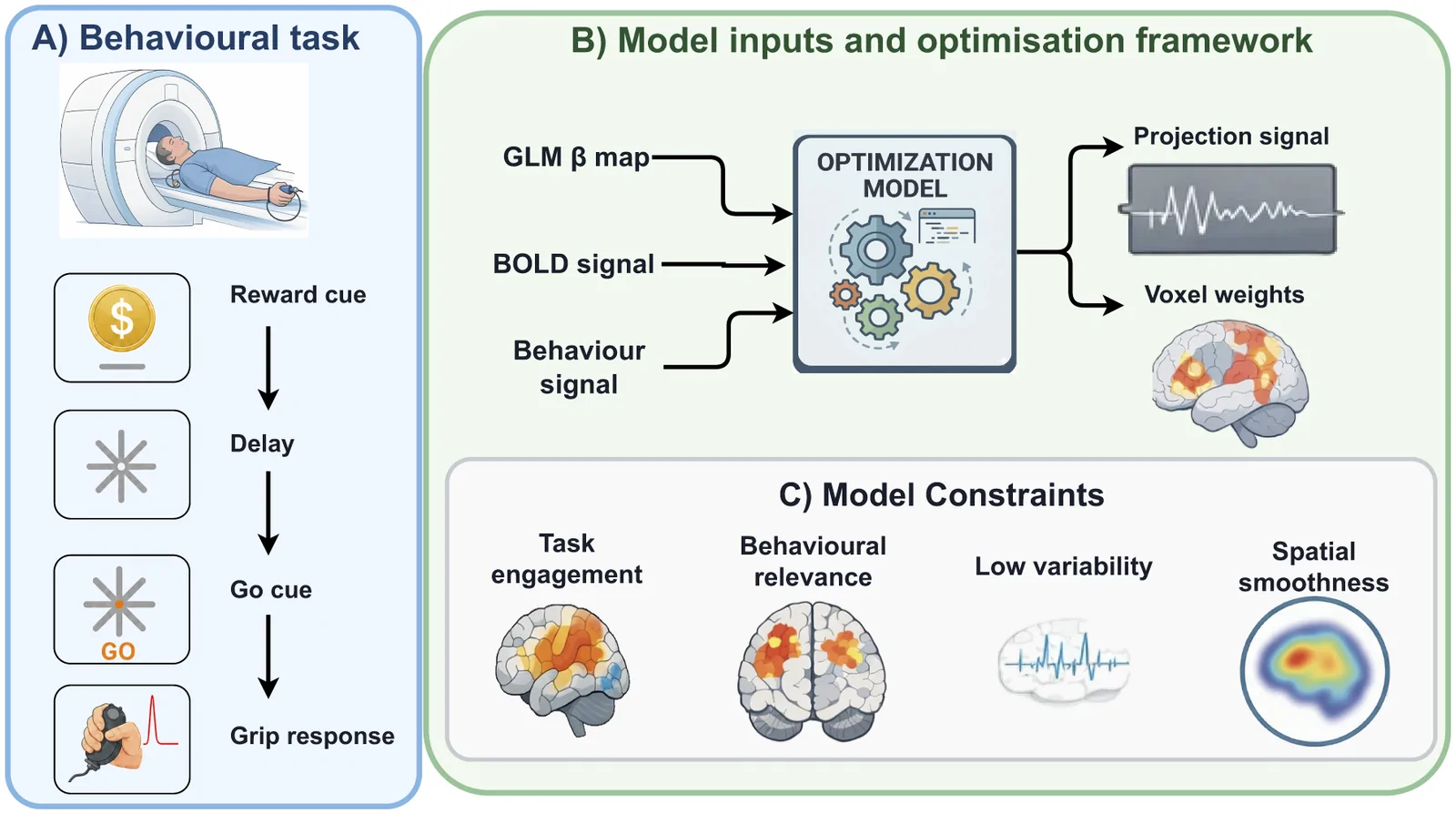
Overview of the behavioral task and analysis pipeline. **(A)**, The task panel shows a reward cue, variable delay, Go signal, grip response, and recorded reaction-time and whole-brain BOLD measures. **(B)**, The analysis panel shows GLMsingle single-trial beta estimates, BOLD-derived features, reaction-time measures, and the constrained optimisation inputs and outputs. **(C)**, The optimisation panel shows the task-engagement, behavior-association, spatial-coherence, and trial-to-trial stability terms used to estimate the projection signal and voxel-weight map.

Each fMRI session consisted of two task runs, and each run included nine blocks of 10 trials. Most trials were rewarded, with low- and high-reward conditions counterbalanced across runs, and 10% were catch trials in which participants were required to withhold a response to avoid a penalty. Task difficulty was adjusted online by varying the response window based on recent hit rate, with the aim of maintaining accuracy at or above 65%. A 20-s rest period was provided after every three blocks. Behavioral quality-control and reward-related RT summaries are reported in Supplementary Figures S1–S3.

GVS (STMISOLA, BIOPAC Systems Inc.) was delivered continuously throughout each run using bilateral mastoid electrodes. Each run contained one sham block and eight active amplitude-modulated GVS blocks. The active waveforms crossed carrier frequency (75 or 125 Hz) with envelope frequency (0, 10, 16, or 25 Hz), producing GVS1-GVS8. The order of the nine stimulation conditions was randomized independently for each run. Stimulation was well tolerated. All active waveforms were delivered at an individually determined amplitude below the perceptual threshold. Participants reported no cutaneous or vestibular sensation and could not distinguish active from sham blocks; they were not informed of the condition order. The GVS analyses are considered exploratory because stimulation was presented in short blocks, observations from the same participant were repeated, and no dedicated washout interval separated every adjacent block. Full stimulation parameters, electrode placement, thresholding, and block-order details are provided in the Supplementary material.

### 2.3 MRI acquisition, pre-processing, and trial-wise beta estimation

Structural and functional MRI (Philips Healthcare, Best, The Netherlands) data were acquired on a 3T Ingenia Elition X whole-body MRI scanner upgraded to an MR7700 system (Philips Healthcare, Best, The Netherlands) using a 32-channel head coil. For each participant, a T1-weighted structural image was acquired, followed by two task-based fMRI runs per medication session. Functional MRI data were acquired using an EPI sequence. The first three participants were scanned with a multi-echo EPI protocol, whereas the remaining participants were scanned with a single-echo EPI protocol. Full sequence parameters are provided in the Supplementary material.

Image pre-processing was performed in FSL (Smith et al., 2004). Structural images underwent bias-field correction and brain extraction. Functional images were corrected for head motion and susceptibility-induced distortion, high-pass filtered, brain extracted, denoised using ICA-AROMA (Pruim et al., 2015), spatially smoothed with a 5 mm full-width at half-maximum Gaussian kernel, registered to each participant's structural image, and normalized to MNI152 space. The scanner automatically discarded four dummy volumes before image data were saved, so no additional initial volumes were removed during pre-processing.

Task-related responses for individual trials were estimated using GLMsingle (Prince et al., 2022), with HRF fitting, GLMdenoise (Kay et al., 2013), and fractional ridge regression enabled. Analyses were performed separately for each subject-session, with the two task runs analyzed jointly while preserving run identity in the design matrix. Go-signal onsets were modeled using 2-s event regressors; catch trials were excluded because no squeeze response was required. The mean CSF time series was included as a nuisance regressor. After robust beta-value cleaning and group-level coverage filtering, the final group beta matrix contained 91, 769 voxels. Detailed beta-cleaning thresholds, outlier rules, and coverage calculations are reported in the Supplementary material.

### 2.4 Stage 1: Vigor network identification and validation

#### 2.4.1 Task-activation map

To provide a conventional task-engagement reference, we ran a standard FSL FEAT GLM separately from the trial-wise GLMsingle model. All valid Go trials were modeled with one event regressor defined at the Go-signal onset, with duration 2 s and amplitude 1. First-level maps tested the positive effect of task activity; runs were combined within subject-session using fixed effects, and subject-session maps were entered into a group mixed-effects model using FLAME stage 1 (Woolrich et al., 2004; Beckmann et al., 2003). Group maps were thresholded using clusters formed at *Z* ≥ 3.1 with corrected cluster significance *p* ≤ 0.05 (Worsley, 2001). This thresholded map is used only as a “task-activation map”, not as the main model. The full standard GLM map and comparisons with GLMsingle activation maps are shown in Supplementary Figures S4, S5.

#### 2.4.2 Constrained optimisation framework

The central analysis is designed to recover a trial-wise neural projection related to motor vigor (Figures 1B, C). The optimisation asks which multivoxel pattern produces a single neural signal that is task-engaged, related to trial-wise RT, spatially coherent, and locally stable across consecutive trials. This framing is important because the study question was not only where the task activates the brain, but whether a relatively stable central signal can be recovered despite behavioral variability.

The inputs are the cleaned single-trial GLMsingle beta estimates, denoted *X*_β_, and the corresponding trial-window BOLD, denoted *X*_BOLD_. Columns represent trials or trial-window samples, and rows represent voxels. Because optimisation in the full 91, 769-voxel space is computationally impractical, both matrices were first reduced using expectation-maximization principal component analysis (EMPCA) (Roweis, 1997). The same EMPCA basis was used for the beta and BOLD matrices. An 80% cumulative explained-variance threshold retained 14 components, and optimisation was performed in this reduced space.

For a candidate weight vector *w*, the beta and BOLD projections are


$$\begin{array}{l} y_{\beta }=w^{⊤}X_{\beta },\;y_{\text{BOLD}}=w^{⊤}X_{\text{BOLD}}, \end{array}$$ (1)

where *y*_β_ is one scalar neural value per trial. The same weight vector is applied to *X*_BOLD_ to quantify over-trial instability in the BOLD signal *y*_*BOLD*_. We refer to this *y*_β_ and *y*_*BOLD*_ throughout the paper as the “projection”. The EMPCA weights are then projected back to voxel space, resulting in a voxel-weight map. Voxels in the upper tail of the map are used to define the vigor network, as described below.

The objective combines four penalty terms with one RT-alignment term. The four penalty terms are defined as follows. *L*_task_ is the task-engagement penalty: it assigns larger penalties to weakly task-engaged components and smaller penalties to components with stronger task-related beta responses. *L*_BOLD_ is the consecutive-trial BOLD instability penalty, formed from squared differences between adjacent projected BOLD feature trials. *L*_β_ is the corresponding consecutive-trial beta instability penalty, formed from squared differences between adjacent single-trial beta feature vectors. *L*_smooth_ is the spatial smoothness penalty, obtained from the voxel-neighbor graph Laplacian. Consecutive-trial variability is used instead of total variance because it preserves trial order: it penalizes abrupt local changes while allowing slower changes across task context, block, or run. Run and block boundaries are excluded from the BOLD and beta instability penalties because adjacent samples across those boundaries do not represent continuous local dynamics.

These four penalties are combined into a single total loss,


$$\begin{array}{r} L_{\text{total}}\left(w\right)=\alpha_{\text{task}}L_{\text{task}}\left(w\right)+\alpha_{\text{BOLD}}L_{\text{BOLD}}\left(w\right)+\\ \alpha_{\beta }L_{\beta }\left(w\right)+\alpha_{\text{smooth}}L_{\text{smooth}}\left(w\right). \end{array}$$ (2)

Here, α_task_, α_BOLD_, α_β_, and α_smooth_ control the relative contributions of task engagement, BOLD local stability, beta local stability, and spatial smoothness, respectively. The behavioral relevance penalty is represented by *L*_RT_, which captures alignment between the projected beta signal and RT. This behavioral penalty term is normalized by *L*_var_, the beta-signal variance matrix, so that the objective favors RT alignment rather than simply selecting high-variance components. The final optimisation problem is


$$\begin{array}{l} \underset{\|w{\|}_{2}=1}{\min}\frac{\gamma L_{\text{total}}\left(w\right)-L_{\text{RT}}\left(w\right)}{L_{\text{var}}\left(w\right)}. \end{array}$$ (3)

The negative sign before *L*_RT_ reflects that RT alignment is maximized, whereas the other terms are penalized. The scalar γ controls the overall balance between regularization and behavioral relevance. These are graded criteria, not hard exclusion rules: a feature is not discarded because it is weak on one term; the final solution is the projection that best balances all criteria. All defined penalty terms are in quadratic form. Their full definitions and derivation are provided in Appendix A.

The constrained optimisation was solved using Sequential Least Squares Programming (SLSQP) (Nocedal and Wright, 2006; Kraft, 1988). Hyperparameters were selected with a coarse-to-fine cross-validation procedure using contiguous folds. We first tested a wide range of candidate settings and then tested smaller proportional changes around the best setting from this first search. Model selection used the held-out absolute correlation between *y*_β_ and RT. The final setting was chosen using held-out RT correlation, stability across folds, and small train-test differences. For each fold, the model was fit on the training data and projected back to voxel space. The fold-specific voxel-weight maps were then averaged to obtain the final voxel-weight map and trial-wise projection. Medication state and GVS condition were not used to define the vigor network; they were analyzed only after the vigor network had been estimated.

#### 2.4.3 Network definition and validation

The vigor network is defined as voxels with weights above the 90th percentile. This threshold was chosen because it provided a stable compromise between spatial specificity and anatomical coverage: lower thresholds retained substantially more voxels, whereas stricter thresholds began to remove several anatomical groups. Threshold robustness is shown in Supplementary Figure S6.

We validated the model in three ways. (i) We assessed map reproducibility using nine-fold participant-level cross-validation, holding out two participants per fold and repeating the full network-definition procedure using only the other 16 participants. (ii) We compared the consecutive-trial variability of the motor vigor projection with the consecutive-trial variability of RT. The normalized consecutive-trial variability metric is


$$\begin{array}{l} \underset{i}{\sum }\frac{{\left(x_{i}-x_{i+1}\right)}^{2}}{x_{i}^{2}+x_{i+1}^{2}}, \end{array}$$ (4)

where *x*_*i*_ denotes the value of the signal on trial *i*, using either the projection or RT depending on the comparison. The summation index *i* therefore runs over adjacent valid trial pairs after excluding run and block boundaries. Inferential statistics used subject-level summaries. (iii) We tested whether vigor network voxels were less variable than a motor-area control pool. The control pool was defined from bilateral motor-area atlas labels after removing selected voxels, and 1, 000 size-matched samples were drawn without replacement. The control distribution was matched to the vigor network by sample size only; samples were not additionally matched by hemisphere, region of interest (ROI) size, spatial location, or local smoothness.

We next assessed whether the defined vigor network was anatomically distinct from the standard task-activation map. Voxel-wise overlap between the vigor network and the task-activation map was quantified using the number of shared voxels, the Dice coefficient (Elin et al., 2022), and the Jaccard index (Maitra, 2010). ROI-level anatomical coverage was summarized as the percentage of each atlas-defined ROI occupied by the vigor network, the task-activation map, and their same-voxel overlap.

### 2.5 Stage 2: network characterization and modulation

These analyses were performed after the vigor network had been defined. We specifically investigated the effects of different GVS stimuli and dopaminergic medication on the defined network.

#### 2.5.1 Exploratory GVS-related network analysis

We first tested whether GVS modulated behavioral vigor. Trial-wise reaction-time data were extracted for each subject, session, run, and stimulation block. The sham condition was used as the reference condition, and the eight GVS waveforms were labeled GVS1-GVS8. To reduce between-subject scaling differences, inverse RT values were z-scored within each subject and session.

Separate mixed-effects models were fitted for the OFF-medication and ON-medication sessions. For each medication session, two nested models were compared. The reduced model included run as a fixed effect, accounting for general differences between runs. The full model included both run and GVS stimuli as fixed effects, with sham as the reference condition. A likelihood-ratio test was used to determine whether adding a GVS stimulus explained additional variance in inverse RT beyond run effects. We then examined the individual GVS coefficients from the full model. Because sham was the reference condition, each coefficient represented the difference between one active GVS waveform and sham. The eight active-GVS-vs.-sham p-values were corrected separately within each medication session using Benjamini-Hochberg false-discovery-rate correction (Benjamini and Hochberg, 1995).

We next examined whether GVS altered the temporal profile of the vigor network signal. As described in Section 2.4.2, trial-wise BOLD time courses were projected onto the vigor network for each trial. For each subject, medication session, run, and GVS stimulus, projected signals were averaged within a stimulation block. The sham block from the same run served as the within-run reference for each active GVS stimulus. This within-run comparison was used to reduce the influence of run-level differences unrelated to stimulation. For visual inspection, subject-level condition time courses were plotted separately for the OFF-medication and ON-medication sessions. These plots included the sham condition and all eight active GVS stimuli. The purpose of these time-course plots is descriptive: they are used to inspect whether different GVS waveforms appear to change the shape, amplitude, or temporal evolution of the projected motor-vigor signal.

To test whether any observed GVS-related changes were specific to the vigor network, the same procedure was repeated for the task-activation map signal. In this control analysis, the projected motor-vigor signal was replaced by the task-activation map BOLD signal, while the same GVS labels, medication-session split, within-run sham reference, and plotting approach were retained. This comparison allows us to assess whether stimulation effects are expressed preferentially in the vigor network or whether similar changes are also present in a task-activation map signal.

To quantify GVS-related changes in the shape of the projected time courses, four predefined features were extracted from each trial-level signal: late-early response difference, linear slope, maximum absolute baseline response, and peak-to-peak amplitude. The late-early response difference is calculated as the mean of the last three TRs minus the mean of the initial three TRs. This feature indicates whether the signal tends to increase or decrease across the trial. The linear slope is obtained by fitting a least-squares regression line to the entire trial time course. It provides a second, complementary measure of gradual change over time. The maximum absolute baseline response is defined as the largest absolute deviation from the value at the first time point, reflecting the strongest departure from the starting level. Finally, the peak-to-peak amplitude is the difference between the maximum and minimum signal values across the trial, representing the overall dynamic range of the response. For each feature, values were first averaged within subject, medication session, run, and GVS stimulus. Each active GVS stimulus condition was then paired with the sham condition from the same subject, medication session, and run. Active-minus-sham feature differences were computed at the run level and then averaged within subject. For each active GVS stimulus and each feature, subject-level active-minus-sham differences were tested against zero using a two-sided sign-flip permutation test. Confidence intervals were estimated using bootstrap resampling. P-values were corrected across the GVS-by-feature family using Benjamini-Hochberg FDR correction (Benjamini and Hochberg, 1995). The same feature-quantification procedure was applied to both the vigor network and the task-activation map.

To compare GVS effects between the two networks directly, we fitted an additional linear mixed-effects model for each temporal feature. Fixed effects were stimulation condition, network, the stimulation-by-network interaction, and medication state; a participant-specific random intercept accounted for repeated observations. Model-based contrasts compared GVS1-GVS8 with sham within each network. Benjamini-Hochberg FDR correction was applied across all 16 contrasts per feature, with significance defined as *q* < 0.05. Full results are reported in Supplementary Figure S15.

Finally, we examined whether GVS modulated functional connectivity within the map-specific sets of voxels assigned to each ROI. For each subject, medication session, run, and GVS stimulus, trial-wise ROI beta estimates were grouped by stimulation block. The sham block served as the within-run baseline, and each GVS waveform was compared with the matched sham block from the same run. Connectivity was computed across 38 ROIs, yielding 703 unique undirected edges for each of the two metrics: Pearson correlation and mutual information. For each GVS block, active-sham edge changes were computed separately for each metric. Thus, the GVS analysis asks whether stimulation changes between-ROI coupling relative to the matched sham condition. Block-level edge-change values were pooled across subjects, medication sessions, runs, and active GVS stimuli. Each edge was tested against zero using a two-sided sign-flip permutation test with 5, 000 permutations, followed by false-discovery-rate (FDR) correction within metric and analysis scope (Benjamini and Hochberg, 1995). Because this analysis included repeated block-level observations from participants and each block contained few trials, GVS findings are treated as exploratory.

#### 2.5.2 Medication-state network analysis

We also characterized the vigor network by examining its response to dopaminergic medication. Medication state was not explicitly used during network optimisation, so this analysis provides an independent characterization of the vigor network. Only participants with data in both medication sessions were included in paired analyses (*n* = 17). Selected voxels were assigned to atlas-defined ROIs, and the optimized voxel weights were used to compute a weighted mean beta series for each ROI. The same connectivity analysis was repeated using the standard task-activation map as a reference network. Unlike the vigor network, the task-activation map did not contain voxel weights; therefore, ROI beta series for this reference network were computed as unweighted averages across task-active voxels within each ROI.

We used two complementary undirected functional connectivity metrics to assess medication effects: (i) mutual information, which tests whether the activity level of one ROI provides information about the activity level of another ROI beyond chance, including dependencies that may not be purely linear, and (ii) Pearson correlation, which captures linear co-fluctuation (Cover and Thomas, 2006). Mutual information was estimated after discretising each beta series into three rank-based quantile bins. Because both metrics are symmetric, related effects were interpreted as changes in statistical coupling rather than causal influence.

We tested whether medication changed the overall ROI-to-ROI connectivity beyond within-session run-to-run variability. For each participant, medication session, run, and connectivity metric, the upper-triangular ROI-pair values from the connectivity matrix were vectorised into an edge vector. Network-pattern dissimilarity between two runs was quantified using correlation distance, defined as 1−|*r*|, where *r* was the Pearson correlation between the two edge vectors. Smaller values, therefore, indicate more similar edge patterns, whereas larger values indicate greater dissimilarity. For each participant and connectivity metric, within-state run-to-run distance was calculated as the mean of the OFF run-1 vs. OFF run-2 distance and the ON run-1 vs. ON run-2 distance. Cross-state distance was calculated as the mean of the four OFF-run to ON-run distances. Medication-related excess distance was then defined as cross-state distance minus within-state run-to-run distance. Positive values indicate that the edge pattern differs more between medication sessions than expected from run-to-run variability alone. Group inference tested whether subject-level excess distance was greater than zero using a sign-flip permutation test. Cohen's *d* was computed as the mean excess distance divided by its subject-level standard deviation (Supplementary Figure S9).

We also tested whether medication preferentially affected local within-ROI coupling or distributed between-ROI coupling. Between-ROI connectivity is the average Fisher-*z*-transformed Pearson correlation between all pairs of ROI beta series. Within-ROI connectivity is the average Fisher-*z*-transformed Pearson correlation between voxel beta series inside each ROI; within-ROI values were averaged equally across ROIs so that larger ROIs did not dominate. The primary contrast tests whether the ON–OFF change in within-ROI connectivity is larger than the ON–OFF change in between-ROI connectivity.

## 3 Results

We present the Results in two stages. Stage 1 describes the identification and validation of the vigor network, including its stability, behavioral relevance, and distinction from standard task activation. Stage 2 examines whether this network is modulated by dopaminergic medication and by different GVS stimuli.

### 3.1 Behavioral dataset and reaction-time profile

Clinical motor severity scores were lower ON than OFF medication: mean MDS-UPDRS Part III scores were 37.3 ± 17.3 OFF medication and 28.0 ± 15.0 ON medication, corresponding to a mean relative reduction from OFF of 26.6 ± 9.1% (Table 1). After trial- and run-level quality control, the final behavioral dataset included 18 participants, 35 sessions, and 70 runs. Mean RT was 389.1 ± 67.4 ms in the OFF-medication session and 375.5 ± 64.7 ms in the ON-medication session, with no significant difference between medication states (mean ON-OFF difference = −13.6 ms, 95% CI [−27.5, 0.3] ms; *t*(16) = −2.08, *p* = 0.054; Supplementary Figure S2). Consecutive-trial RT variability also did not differ significantly between sessions (OFF: 85.5 ± 41.8 ms; ON: 83.4 ± 29.2 ms; mean ON-OFF difference = −2.1 ms, 95% CI [−18.2, 14.0] ms; *t*(16) = −0.27, *p* = 0.787). Reward effects varied across participants, with some showing faster responses in high-reward trials and others showing minimal or opposite reward-related changes (Supplementary Figure S3).

### 3.2 Stage 1: identification and validation of the vigor network

This stage addresses the central question of whether a reproducible, behavior-linked neural network could be recovered that was more stable than RT and distinct from standard task activation.

#### 3.2.1 The optimized projection was reproducible and less variable than RT

Across the nine participant-level cross-validation folds, the training-derived voxel-weight maps showed moderate reproducibility [ICC(2,1) of voxel weights = 0.68]. At the subject level, the vigor-network projection showed lower consecutive-trial variability than RT, with a positive behavior-minus-projection difference (mean difference = 7.8, 95% CI [2.9, 12.7]; paired *p* = 0.004; Figure 2A). Thus, the optimized projection preserved behavioral relevance while varying more smoothly than RT.

**Figure 2:**
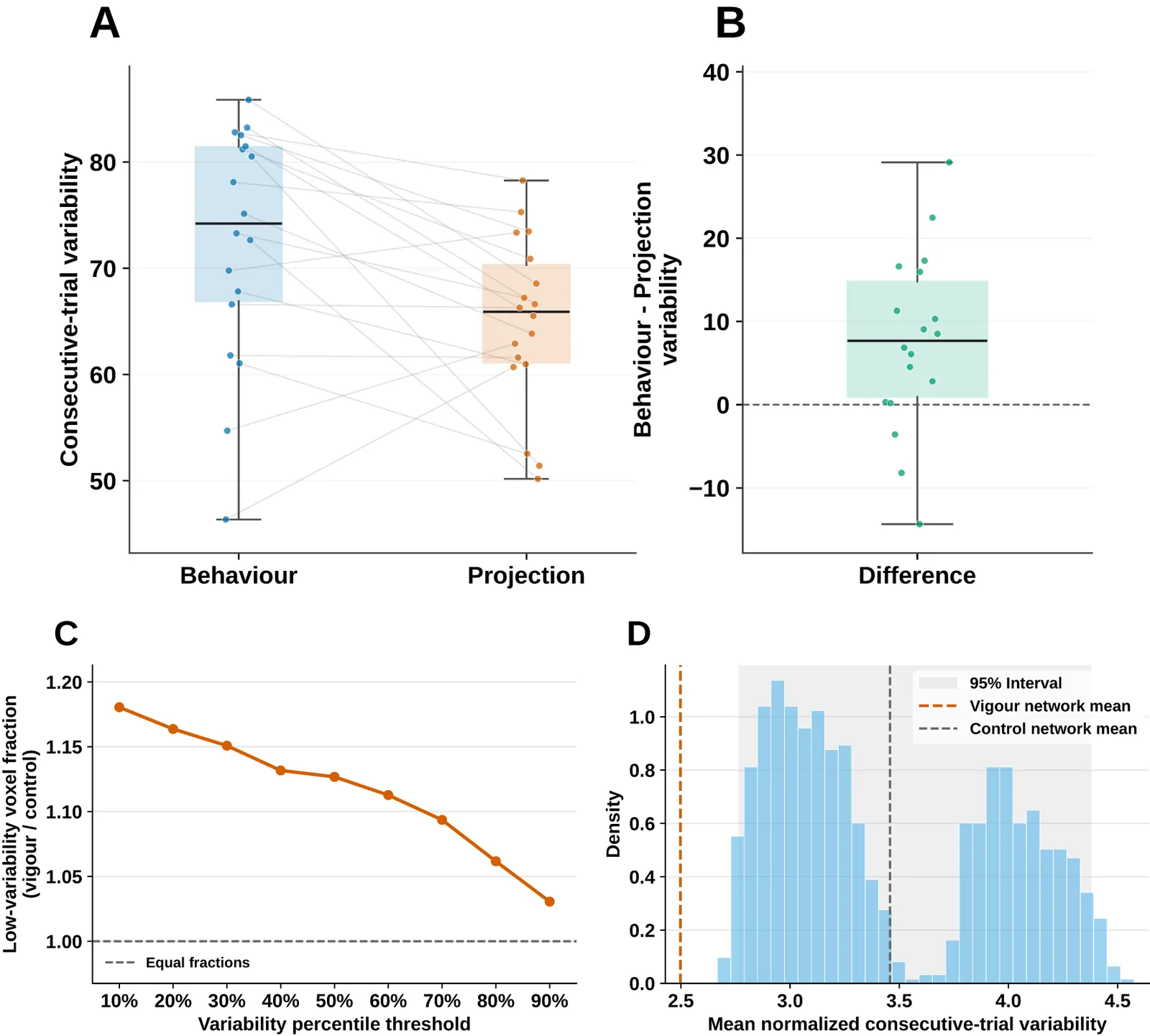
Validation displays for the optimisation framework. **(A)** Subject-level consecutive-trial variability in reaction time and in the vigor-network projection. Points denote subjects and gray lines show paired subject estimates. **(B)** Within-subject behavior-minus-projection differences. **(C)** Across variability percentile thresholds, vigor-network voxels are enriched for voxels with low consecutive-trial variability relative to motor-control voxels. **(D)** The mean normalized consecutive-trial variability of vigor-network voxels is compared with the matched motor-control resampling distribution.

Selected voxels also showed lower consecutive-trial beta variability than size-matched samples from the non-selected motor-area control pool (vigor network mean = 2.50; control mean = 3.46; 95%CI [2.76, 4.38]; *p* = 0.001 from 1, 000 resamples; Figure 2B). The threshold-dependent enrichment and control resampling distribution are shown in Figures 2C, D.

Together, these analyses indicated that the model recovered a behavior-linked neural projection with lower consecutive-trial variability than RT. Because reduced consecutive–trial variability was part of the optimisation objective, this result serves as a model verification rather than independent evidence of neural stability.

#### 3.2.2 The vigor network is anatomically distributed

The vigor network comprises 8, 182 voxels and is distributed across both hemispheres (Figure 3), and is not limited to a single motor cluster. The largest portions of the map are in temporal, parietal, and frontal cortices. A sensorimotor component is also present in precentral, postcentral, paracentral, and supplementary motor areas. Additional clusters extend into the orbitofrontal cortex, occipital cortex, fusiform and parahippocampal regions, hippocampus, amygdala, caudate, putamen, thalamus, and cerebellum (Figure 3 and Supplementary Figure S8).

**Figure 3:**
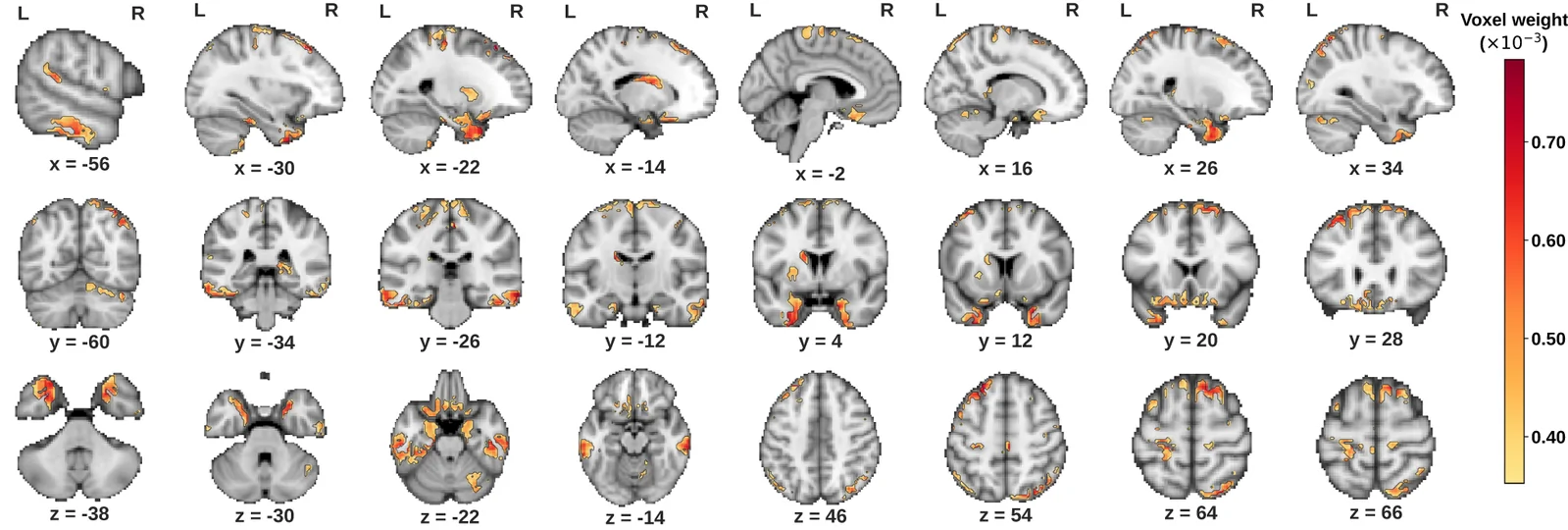
Spatial distribution of the vigor network. Thresholded voxel weights are shown on representative sagittal, coronal, and axial MNI slices. Warmer colors show larger model-derived voxel contributions, scaled by 10^−3^.

Overall, the optimized map reflects a distributed network that includes motor–related regions but extends well beyond primary movement–related areas, with strong coverage in temporal, parietal, frontal, limbic, subcortical, and cerebellar regions.

#### 3.2.3 The vigor network is distinct from standard task activation

We compared the vigor network with the task-activation map to test whether the vigor network is mainly a subset of the task-activation map (Figures 4, 5). The two maps show limited same-voxel overlap. The vigor network contains 8, 182 voxels, whereas the task-activation map contains 21, 610 voxels. Only 835 voxels are shared, giving a Dice coefficient of 0.056 and a Jaccard index of 0.029.

**Figure 4:**
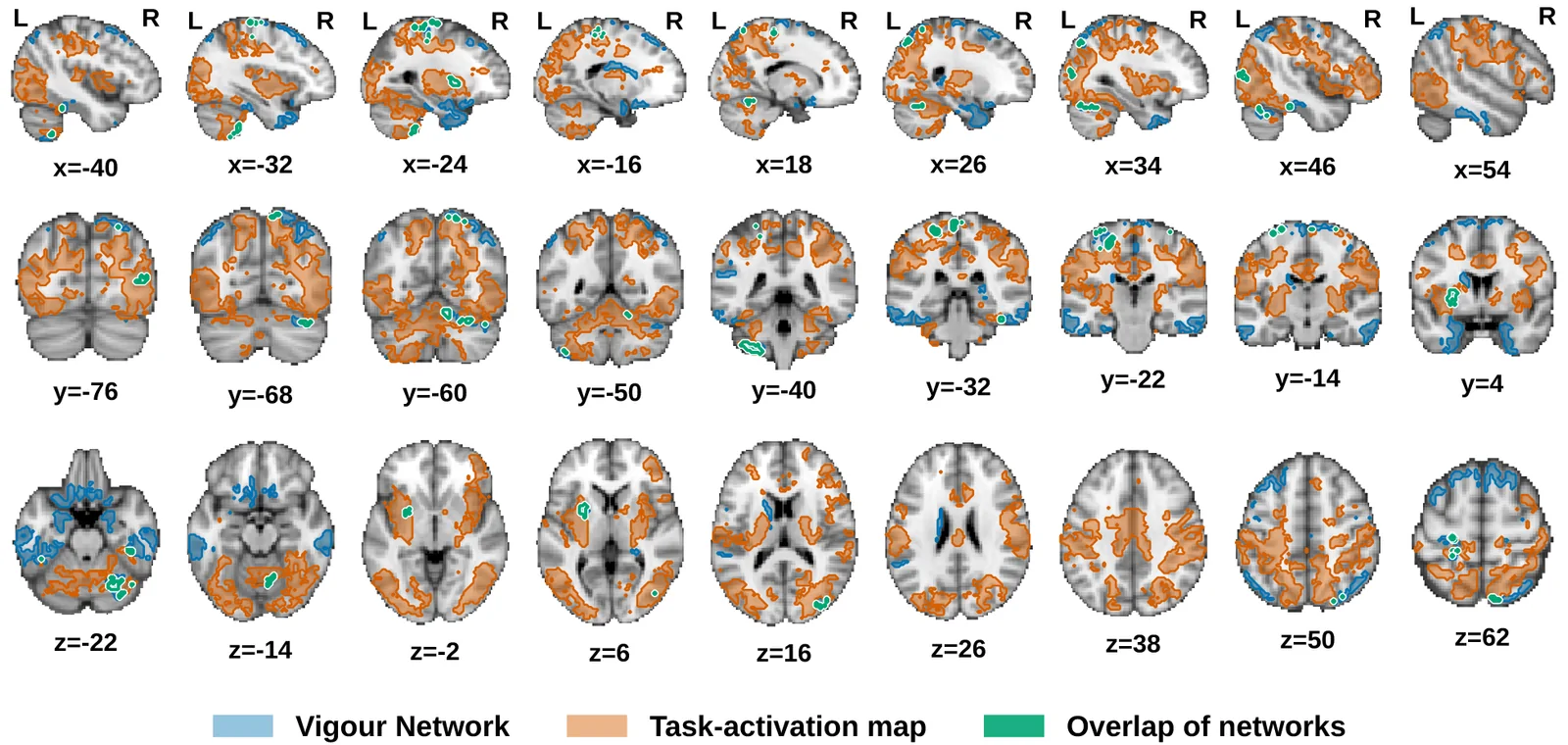
Voxelwise overlap between the vigor network and the standard task-activation map. Blue marks the vigor network, orange marks the task-activation map, and green marks the same-voxel overlap across representative brain slices.

**Figure 5:**
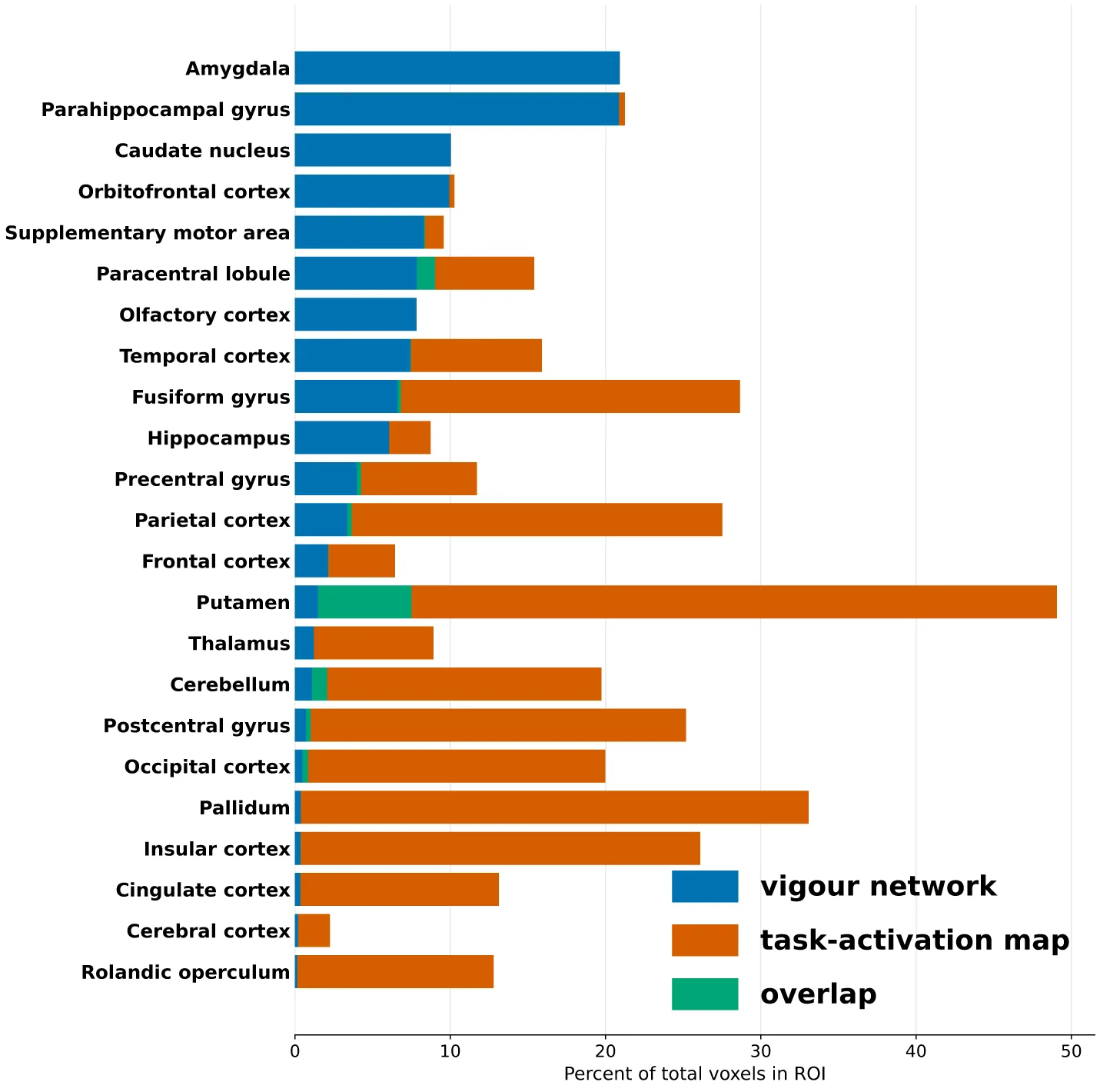
Anatomical comparison by ROI for the vigor network, the standard task-activation map, and their same-voxel overlap. Horizontal bars show the percentage of each ROI occupied by the vigor network, the task-activation map, and overlapping voxels. ROI labels are grouped by anatomical sector, with separate bars for vigor network, task activation, and overlap values.

The task–activation map broadly covers regions engaged during task performance, whereas the vigor network is more selective. Some overlap occurs in motor–related regions-including pericentral cortex, supplementary/paracentral motor areas, putamen, cerebellum, and parietal cortex-but many regions contain both maps with little or no same–voxel overlap. Temporal–limbic and orbitofrontal components are more prominent in the vigor network than in the task–activation map (Figure 5; Supplementary Tables S3, S4).

### 3.3 Stage 2: Stimulation and medication sensitivity of the vigor network

After validating the network, we tested whether its functional organization changed with dopaminergic medication and with different GVS stimuli.

#### 3.3.1 GVS-specific modulation of behavior and network responses

Across the eight GVS waveforms, the behavioral effect of GVS depended on the medication state. In the OFF-medication session, GVS7 was the only GVS waveform that differed significantly from sham after correction for multiple comparisons, producing faster responses, reflected by lower normalized RTs (standardized paired effect size *d*_*z*_ = −0.64; *p* = 0.004, FDR *q* = 0.033; Figure 6 first row). No GVS waveform survived correction in the ON-medication session.

**Figure 6:**
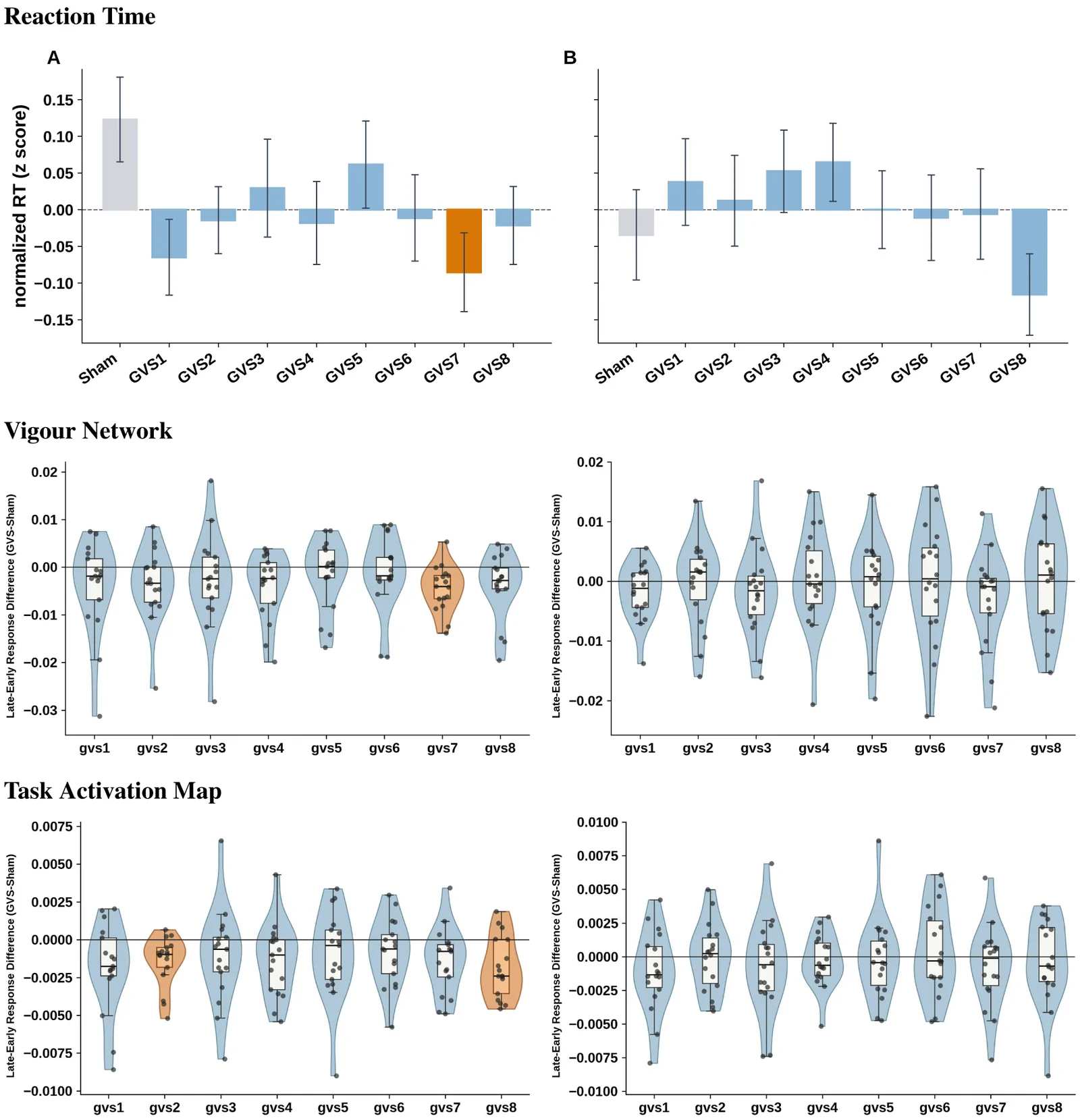
GVS effects on reaction time and projected signal dynamics. Top row: normalized reaction time across sham and GVS1-GVS8 conditions during medication OFF **(A)** and medication ON **(B)**; lower normalized RT indicates faster responses, and error bars show SEM. Middle row: subject-level GVS-minus-sham late-early response differences from the vigor network projected signal. Bottom row: the same late-early feature computed from the task-activation map BOLD signal. In the neural panels, violins show the subject distribution, boxes show median/IQR, and points show individual subjects after pairing each GVS waveform with sham within subject/session/run. The horizontal zero line indicates no difference from sham. Orange highlights GVS waveforms surviving multiple-comparison correction; blue indicates non-significant GVS waveform.

Projected time courses suggested stimulus–specific modulation of the vigor-network signal. Across GVS conditions, the vigor–network projection showed distinct waveform and medication-dependent patterns (Supplementary Figure S12A). For the *late-early response* feature, GVS7 was the only GVS waveform that differed from sham in the OFF-medication session, with a standardized paired effect size of *d*_*z*_ = −0.94 (Figure 6 middle row). The negative active-minus-sham difference indicated that GVS7 reduced the late relative to the early component of the projected vigor network response. This effect was absent during the ON-medication session, in which no GVS waveform survived correction. The full vigor network feature summaries appear in Supplementary Figures S13A, B.

In contrast, the task-activation map projection did not mirror this behavioral specificity. In the ON-medication session, no task-activation map response survived correction, similar to the vigor network. However, in the OFF-medication session, significant task-activation map effects appeared for other stimulation conditions (e.g., GVS2 (*d*_*z*_ = −0.86) and GVS8 (*d*_*z*_ = −0.92) for the late-early feature; GVS6 for the maximum absolute baseline-response feature; Figure 6 bottom row; Supplementary Figures S12B, S14A, B). These separate analyses suggested different waveform patterns, which were tested directly in the joint model.

In the joint model containing the stimulation-by-network interaction, GVS7 was the only condition with FDR-significant contrasts. In the vigor network, the GVS7-sham difference was −0.00361 for the early-late change (95% CI [−0.00579, −0.00143], *p* = 0.00117, *q* = 0.0187) and −0.000562 for slope (95% CI [−0.000915, −0.000208], *p* = 0.00183, *q* = 0.0293). No other contrast survived the same correction, including GVS7 in the task-activation map. Thus, under the same model and multiplicity correction, the GVS7 temporal effect was localized to the vigor network (Supplementary Figure S15).

We next tested whether GVS produced stimulation-sensitive connectivity changes in the vigor network and task-activation map. Under both mutual-information and Pearson-correlation analyses, active GVS was associated with FDR-significant sham-referenced edge changes in each network. The two metrics identified different edge sets, and only a small minority of significant edges overlapped between the vigor network and task-activation map. This descriptive overlap does not establish that GVS effects differed statistically between the network definitions. Because each condition comprised only 10 trials per run and repeated block-level observations were pooled, these findings are exploratory and indicate network perturbability rather than a waveform-specific or causal therapeutic effect. Detailed edge counts, anatomical patterns, and connectivity maps are reported in Supplementary Figures S16, S17.

#### 3.3.2 [

Medication was associated with stronger local coupling within the vigor network] Medication was associated with stronger local coupling within the vigor network We next tested whether dopaminergic medication altered vigor-network connectivity. Network-pattern dissimilarity analysis showed that connectivity patterns differed more between medication states than between repeated runs within the same state, for both Pearson- and mutual-information-based edges (Supplementary Figure S9).

We then assessed whether medication-related reorganization occurred locally within ROIs or between ROIs (Figure 7). Medication was mainly associated with increased local connectivity within vigor-network ROIs. Within-ROI FC increased significantly from OFF to ON-medication (Δ_ON–OFF_ = +0.0147, *p* = 0.006), whereas between-ROI FC showed no reliable change and remained close to zero (Δ_ON–OFF_ = −0.0006, *p* = 0.723). This suggests that dopaminergic medication strengthened local functional coupling within the vigor network rather than broadly increasing distributed connectivity between ROIs.

**Figure 7:**
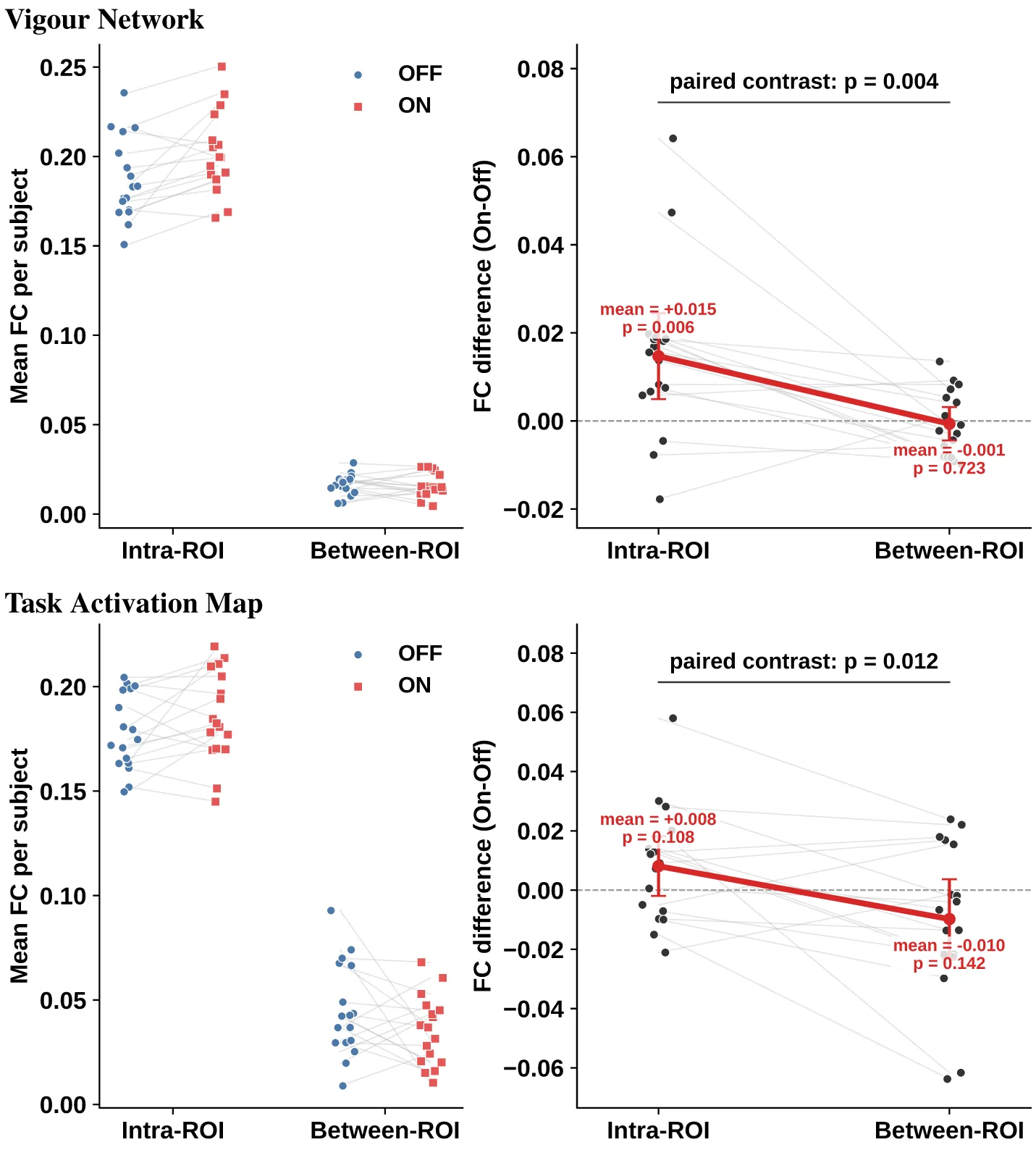
Medication-state functional connectivity summaries. For the vigor network, plots show within-ROI and between-ROI Pearson functional connectivity for OFF-medication, ON-medication, and participant-level ON-OFF differences. The corresponding plots show within-ROI and between-ROI Pearson functional connectivity for the standard task-activation map.

This pattern remained after adjustment for session-level mean framewise displacement: the ON-OFF within-ROI difference remained significant (β = 0.0159, *p* = 0.0019), whereas the between-ROI difference remained non-significant (β = −0.0013, *p* = 0.523; Supplementary material).

To assess whether this pattern was specific to the vigor network, we repeated the same analysis using the task-activation map. The task-activation map also showed a significant difference between within-ROI and between-ROI medication-related changes (*p* = 0.012; Figure 7). However, the underlying pattern was weaker and less specific than in the vigor network. In the vigor network, the effect was driven by a significant increase in within-ROI FC. In the task-activation map, within-ROI FC showed only a non-significant increase (Δ_ON–OFF_ = +0.0081, *p* = 0.108), together with a non-significant tendency toward reduced between-ROI FC (Δ_ON–OFF_ = −0.0098, *p* = 0.142). Thus, while task-active regions also exhibited a local-vs.-distributed medication contrast, the vigor network more clearly captured medication-related strengthening of local coupling.

## 4 Discussion

This study investigated whether trial-wise variability in motor vigor in PD reflects instability in a central vigor-related network, or whether some behavioral variability arises downstream from a relatively stable original motor command. The results are more consistent with the latter interpretation. Using fMRI during a ballistic hand-squeeze task, we identified a distributed vigor network that tracked RT while showing lower consecutive-trial variability than the behavioral signal itself. The main finding is therefore not simply that RT was associated with brain activity. Rather, our results suggest a behavior-linked neural expression of motor vigor in PD that was more stable than the behavior. The vigor network includes a motor-related core with contributions from pericentral cortex, superior parietal cortex, putamen, and cerebellar regions. This is consistent with prior work implicating motor cortical, basal-ganglia, thalamic, and cerebellar systems in movement speed, force, amplitude, and vigor (Anderson et al., 2012; Dudman and Krakauer, 2016; Shirinbayan et al., 2019; Spraker et al., 2007; Turner and Desmurget, 2010; Yttri and Dudman, 2016; Niv et al., 2007; Zénon et al., 2016). Amygdala involvement is also plausible because primate amygdala neurons encode positive and negative visual-stimulus value during learning (Paton et al., 2006), and anatomical evidence supports an amygdala-motor pathway through which affective or salient cues can shape action (Grèzes et al., 2014). Hippocampal and parahippocampal involvement may reflect contextual organization of action: reward behavior depends on hippocampus-nucleus accumbens synaptic strength (LeGates et al., 2018), the hippocampus can organize upcoming skilled action sequences (Yewbrey and Kornysheva, 2024), and hippocampal mechanisms support visually cued contextual response selection (Kim et al., 2012). Thus, the vigor network includes regions that can integrate cue value, context, salience, motivational state, and action readiness to determine whether an action is initiated quickly on a given trial (Wallis, 2007; Rolls et al., 2020; Pierrieau et al., 2025).

The present findings are difficult to reconcile with a simple interpretation in which variable motor vigor solely reflects an unstable central vigor-related network. Instead, they suggest that a relatively stable behavior-linked projection can be recovered in PD, and that some behavioral fluctuation in movement initiation may arise as this command is converted into movement. Importantly, this does not mean that lower BOLD variability is always better: for example, in expert racing drivers, higher BOLD temporal variability has been interpreted as part of a more efficient task-related architecture, whereas in an adult lifespan working-memory sample, higher BOLD variability was linked to poorer performance (Grady and Garrett, 2014; Boylan et al., 2021). The direction of the effect, therefore, has to be interpreted in relation to the task, population, and neural system being studied.

The GVS analyses suggest that the vigor network is sensitive to external vestibular perturbation, but not in a general or uniform manner. Among the GVS waveforms, only GVS7 (125 Hz carrier and 16 Hz envelope) produced a corrected behavioral effect, with faster responses in the OFF-medication session. Importantly, this behaviourally effective waveform was also the only GVS waveform that altered the late-early feature of the vigor network projection in the same medication state. This pattern was not reproduced by the task-activation network: significant task-activation map effects appeared for other stimulation conditions rather than for GVS7. Thus, the key finding is not simply that GVS altered both behavior and brain activity, but that the only stimulation waveform associated with improved behavioral vigor was also selectively reflected in the vigor-related projection. This supports the interpretation that the vigor network captures behaviourally relevant stimulation effects more specifically than the broader task-activation network. Exploratory connectivity analyses found FDR-significant active-vs.-sham edge changes under both connectivity metrics in both networks. Only a small minority of the significant edges overlapped between maps, showing that the identified stimulation-sensitive edge sets were partly non-overlapping; however, this descriptive result is not a formal between-network comparison. Given the small number of trials per block and the pooled repeated observations, these findings indicate network perturbability rather than a waveform-specific or therapeutic connectivity mechanism (Supplementary Figures S16, S17).

The medication analysis shows that the vigor network is not fixed across the dopaminergic state. Connectivity patterns within the network differed more between OFF and ON-medication than between repeated runs within the same medication state, for both Pearson-based and mutual-information-based measures. More specifically, ON-medication was associated with a selective increase in local within-ROI coupling, whereas between-ROI coupling remained close to zero and did not change reliably. This pattern was clearer in the vigor network than in the task-activation map: it showed a significant local-vs.-distributed contrast, but its separate within-ROI and between-ROI changes were weaker and individually non-significant. Thus, the main medication-related finding is not a global increase in network connectivity, nor is there evidence that medication normalized the network. Rather, a dopaminergic state was associated with stronger local coherence within the vigor network. This interpretation is consistent with prior work linking dopamine to motor vigor and with evidence that levodopa can alter functional network organization in PD (Niv et al., 2007; Berman et al., 2016; Zénon et al., 2016; Mazzoni et al., 2007).

This study has several limitations. Because the study included only participants with PD, we cannot determine whether the projection is specific to PD or reflects more general features of the task or analysis pipeline. The sample included only four female participants, precluding a meaningful assessment of sex-related differences and limiting the generalisability of the findings across sexes. Motor vigor was indexed mainly by reaction time, which captures response initiation; however, other components such as movement speed, force amplitude, force rise time, and movement duration are also important aspects of motor vigor and may dissociate depending on task demands (Pierrieau et al., 2025). The fixed OFF-then-ON also limits causal interpretation of medication order (necessitated for safety and comfort of the participants), which may confound medication effects with practice, fatigue, time-on-task, or scanner drift. In addition, reward cues, GVS stimulus, block context, and adaptation across the run may have contributed to both RT and trial-wise neural activity, not just the vigor network. A related methodological caveat is that BOLD variability can include non-neural vascular contributions; scaling with resting-state fluctuation amplitude or breath-hold responses can reduce this variability, especially in block-design motor fMRI (Kannurpatti et al., 2011). Finally, the optimisation was linear and was developed and characterized in the same dataset. Cross-validation and control analyses reduce this concern, but independent validation is needed in new PD cohorts, healthy adults, and different motor tasks. An argument could be made that our results are the result of a circular argument: low consecutive-trial variability was one optimisation criterion. However, the model was constrained not only by stability, but also by task engagement, behavioral relevance, and spatial coherence. At the subject level, the optimized projection was less variable than RT. At the voxel level, the selected voxels were concentrated among low-variability voxels and showed lower mean variability than size-matched samples from non-selected motor-area voxels. These validation analyses support the view that the solution was neither an arbitrary smooth sample of motor activity nor simply a restatement of behavioral variability.

Overall, the present study suggests that trial-wise vigor variability in PD should not be interpreted simply as instability of a central vigor-related command. A relatively stable, behavior-linked neural activity can be recovered, and this activity is organized across motor, striatal, limbic, contextual, and motivational systems. Behavioral variability may therefore partly reflect how central vigor-related coding is converted into movement, and the expression of this upstream network was associated with dopaminergic state and modulated by vestibular stimulation.

## 5 Conclusions

We identified a distributed vigor-related neural projection in Parkinson's disease whose trial-wise expression was more stable than the behavioral reaction-time fluctuations it tracked. This projection was anatomically distributed, differed from standard task activation, and showed higher local within-ROI coupling in ON than OFF sessions. GVS analyses further suggested that one OFF-medication stimulation waveform produced a behavioral effect mirrored by the vigor network projection, while broader connectivity analyses revealed heterogeneous stimulation-dependent perturbations. Together, the findings are consistent with a model in which variability in response initiation observed in this PD cohort is not solely the product of a highly variable central command. Instead, a relatively stable central vigor-related network can be recovered, while behavioral variability may partly reflect how that projection is translated into motor output under different dopaminergic and stimulation states. However, because there was no control group or independent cohort, the PD specificity and external generalisability of this projection remain unknown. This vigor network could provide a hypothesis-generating framework for future studies testing the effects of non-invasive stimulation on motor vigor, with the longer-term aim of mitigating some of the motor effects of PD.

## Data Availability

The raw data supporting the conclusions of this article will be made available by the authors, without undue reservation.

## Appendix A: Optimization derivation and model-selection summary

The Optimization framework identifies a voxel-weight vector and a projected signal related to trial-wise motor vigour while satisfying constraints on task engagement, spatial coherence, and low consecutive-trial variability. In voxel space, the projected signals ${\text{y}}_{\beta }\in {\mathbb{R}}^{n_{\text{trials}}}$ and ${\text{y}}_{\text{BOLD}}\in {\mathbb{R}}^{n_{\text{time}}}$ are defined as:

$$\begin{array}{l} {\text{y}}_{\beta }={\text{w}}^{⊤}{\text{X}}_{\beta }\;{\text{y}}_{\text{BOLD}}={\text{w}}^{⊤}{\text{X}}_{\text{BOLD}} \end{array}$$

${\text{X}}_{\beta }\in {\mathbb{R}}^{n_{\text{voxels}}\times n_{\text{trials}}}$ contains the single-trial beta estimates, ${\text{X}}_{\text{BOLD}}\in {\mathbb{R}}^{n_{\text{voxels}}\times n_{\text{time}}}$ contains the BOLD time courses, and $\text{w}\in {\mathbb{R}}^{n_{\text{voxels}}}$ denotes the model weights. The Optimization model is based on four constraints. First, the signal should come mainly from task-engaged voxels. This term discourages the model from assigning large weights to weakly task-engaged components. For voxel *v*, let *a*_*v*_ = ||*X*_β, *v*:_||_2_, $\text{a}={\left(a_{1},\ldots ,a_{n_{\text{voxels}}}\right)}^{⊤}$ where *X*_β, *v*:_ is the trial-wise beta for voxel *v*. The task-engagement penalty is

$$L_{\text{task}}\left(w\right)=w^{⊤}C_{\text{task}}w,\;C_{\text{task}}={\left[\mathrm{diag}\left(\text{a}+\epsilon 1\right)\right]}^{-1},$$

where ϵ > 0 prevents division by zero. Second, the projected beta signal should encode behavioural information. The behavioural variable is reaction time. Therefore, the model aims to maximise the correlation between *X*_behav_ (RT) and *y*_β_:

$$\begin{array}{l} \underset{w}{\max}\mathrm{Corr}{\left(X_{\text{behav}},w^{⊤}X_{\beta }\right)}^{2}=\frac{w^{⊤}X_{\beta }X_{\text{behav}}^{⊤}X_{\text{behav}}X_{\beta }^{⊤}w}{w^{⊤}X_{\beta }X_{\beta }^{⊤}w}=\frac{w^{⊤}C_{\text{RT}}w}{w^{⊤}C_{\text{var}}w} \end{array}$$

Third, the selected voxels should form a spatially coherent network. Neighbouring voxels are encouraged to have similar weights using the spatial smoothness loss: $L_{\text{smooth}}\left(w\right)=w^{⊤}C_{\text{smooth}}w$, *C*_smooth_ = *L*_graph_.

Fourth, the projected signal should show low consecutive-trial variability. This condition penalises large local changes between neighbouring trials, rather than variance across the full time. For valid consecutive-trial pairs *I*_*c*_, the beta estimate variability loss is

$$\begin{array}{l} L_{\beta }\left(w\right)=w^{⊤}C_{\beta }w,\;C_{\beta }=\underset{i\in I_{c}}{\sum }\left(X_{\beta ,i}-X_{\beta ,i+1}\right){\left(X_{\beta ,i}-X_{\beta ,i+1}\right)}^{⊤} \end{array}$$

The same low-variability condition is also applied to BOLD: $L_{BOLD}\left(w\right)=w^{⊤}C_{BOLD}w$. Both variability terms are included because *L*_β_(*w*) constrains variability in movement-related beta responses, whereas *L*_BOLD_(*w*) also captures broader task-related variability present in the BOLD.

The four penalty terms are combined into a single total loss:

$$\begin{array}{l} \begin{array}{c} L_{\text{total}}\left(w\right)=\alpha_{\text{task}}L_{\text{task}}\left(w\right)+\alpha_{\text{BOLD}}L_{\text{BOLD}}\left(w\right)+\alpha_{\beta }L_{\beta }\left(w\right)\\ +\alpha_{\text{smooth}}L_{\text{smooth}}\left(w\right)=w^{⊤}C_{\text{total}}w\\ C_{\text{total}}=\alpha_{\text{task}}C_{\text{task}}+\alpha_{\text{BOLD}}C_{\text{BOLD}}+\alpha_{\beta }C_{\beta }+\alpha_{\text{smooth}}C_{\text{smooth}} \end{array} \end{array}$$

The final objective minimises the combined penalties while maximising behavioural relevance:

$$\begin{array}{l} \underset{w}{\min}\frac{\gamma w^{⊤}C_{\text{total}}w-w^{⊤}C_{RT}w}{w^{⊤}C_{var}w}\text{ s}.\text{t}.\;\|w{\|}_{2}=1 \end{array}$$

Here, α_task_, α_BOLD_, α_β_, and α_smooth_ control the relative contribution of each penalty term, and γ controls the overall strength of the penalties relative to behavioural relevance.

The Optimization was solved with Sequential Least Squares Programming, and hyperparameters were selected by coarse-to-fine cross-validation. Candidate settings were fitted on training folds and evaluated using held-out |Corr(*y*_β_, *RT*)|, favouring stable performance and small train-test differences. Since voxel-space Optimization was computationally impractical at this dimensionality, the data were first projected into a lower-dimensional PCA subspace.

## References

[B1] Anderson N. R. Blakely T. Schalk G. Leuthardt E. C. Moran D. W. (2012). Electrocorticographic (ECoG) correlates of human arm movements. Exp. Brain Res. 223, 1–10. doi: 10.1007/s00221-012-3226-1 23001369

[B2] Beckmann C. F. Jenkinson M. Smith S. M. (2003). General multilevel linear modeling for group analysis in fMRI. Neuroimage 20, 1052–1063. doi: 10.1016/S1053-8119(03)00435-X 14568475

[B3] Benjamini Y. Hochberg Y. (1995). Controlling the false discovery rate: a practical and powerful approach to multiple testing. J. Royal Statistical Society: Series B (Methodol) 57, 289–300. doi: 10.1111/j.2517-6161.1995.tb02031.x

[B4] Berman B. D. Smucny J. Wylie K. P. Shelton E. Kronberg E. Leehey M. . (2016). Levodopa modulates small-world architecture of functional brain networks in Parkinson's disease. Mov. Disord. 31, 1676–1684. doi: 10.1002/mds.26713 PMC5115928 27461405

[B5] Berret B. Castanier C. Bastide S. Deroche T. (2018). Vigour of self-paced reaching movement: cost of time and individual traits. Sci. Rep. 8:10655. doi: 10.1038/s41598-018-28979-6 PMC6045586 30006639

[B6] Boylan M. A. Foster C. M. Pongpipat E. E. Webb C. E. Rodrigue K. M. Kennedy K. M. (2021). Greater bold variability is associated with poorer cognitive function in an adult lifespan sample. Cerebral Cortex 31, 562–574. doi: 10.1093/cercor/bhaa243 PMC7727366 32915200

[B7] Brainard D. H. (1997). The psychophysics toolbox. Spatial Vision 10, 433–436. doi: 10.1163/156856897X00357 9176952

[B8] Cai J. Lee S. Ba F. Garg S. Kim L. J. Liu A. . (2018). Galvanic vestibular stimulation (GVS) augments deficient pedunculopontine nucleus (PPN) connectivity in mild Parkinson's disease: fMRI effects of different stimuli. Front. Neurosci. 12:101. doi: 10.3389/fnins.2018.00101 PMC5835530 29541016

[B9] Churchland M. M. Santhanam G. Shenoy K. V. (2006). Preparatory activity in premotor and motor cortex reflects the speed of the upcoming reach. J. Neurophysiol. 96, 3130–3146. doi: 10.1152/jn.00307.2006 16855111

[B10] Cover T. M. Thomas J. A. (2006). Elements of Information Theory. Hoboken, NJ: Wiley-Interscience.

[B11] Dudman J. T. Krakauer J. W. (2016). The basal ganglia: from motor commands to the control of vigor. Curr. Opinion Neurobiol. 37, 158–166. doi: 10.1016/j.conb.2016.02.005 27012960

[B12] Elin K. Malyutina S. Bronov O. Stupina E. Marinets A. Zhuravleva A. . (2022). A new functional magnetic resonance imaging localizer for preoperative language mapping using a sentence completion task: validity, choice of baseline condition, and test-retest reliability. Front. Human Neurosci. 16:791577. doi: 10.3389/fnhum.2022.791577 PMC9006995 35431846

[B13] Faisal A. A. Selen L. P. Wolpert D. M. (2008). Noise in the nervous system. Nature Reviews Neurosci. 9, 292–303. doi: 10.1038/nrn2258 PMC2631351 18319728

[B14] Fitzpatrick R. C. Day B. L. (2004). Probing the human vestibular system with galvanic stimulation. J Appl. Physiol. 96, 2301–2316. doi: 10.1152/japplphysiol.00008.2004 15133017

[B15] Garrett D. D. Samanez-Larkin G. R. MacDonald S. W. Lindenberger U. McIntosh A. R. Grady C. L. (2013). Moment-to-moment brain signal variability: a next frontier in human brain mapping? Neurosci. and Biobehav. Rev. 37, 610–624. doi: 10.1016/j.neubiorev.2013.02.015 PMC3732213 23458776

[B16] Goetz C. G. Tilley B. C. Shaftman S. R. Stebbins G. T. Fahn S. Martinez-Martin P. . (2008). Movement disorder society-sponsored revision of the Unified Parkinson's Disease Rating Scale (MDS-UPDRS): scale presentation and clinimetric testing results. Movement Disord.: Official J. Mov. Disord. Society 23, 2129–2170. doi: 10.1002/mds.22340 19025984

[B17] Grady C. L. Garrett D. D. (2014). Understanding variability in the bold signal and why it matters for aging. Brain Imag. Behav. 8, 274–283. doi: 10.1007/s11682-013-9253-0 PMC3922711 24008589

[B18] Grèzes J. Valabregue R. Gholipour B. Chevallier C. (2014). A direct amygdala-motor pathway for emotional displays to influence action: A diffusion tensor imaging study. Human Brain Mapping 35, 5974–5983. doi: 10.1002/hbm.22598 25053375 PMC6869045

[B19] Haar S. Donchin O. Dinstein I. (2017). Individual movement variability magnitudes are explained by cortical neural variability. J. Neurosci. 37, 9076–9085. doi: 10.1523/JNEUROSCI.1650-17.2017 PMC6596801 28821678

[B20] Harris C. M. Wolpert D. M. (1998). Signal-dependent noise determines motor planning. Nature 394, 780–784. doi: 10.1038/29528 9723616

[B21] Kannurpatti S. S. Motes M. A. Rypma B. Biswal B. B. (2011). Non-neural bold variability in block and event-related paradigms. Magnetic Resonance Imag. 29, 140–146. doi: 10.1016/j.mri.2010.07.006 PMC10653950 20833501

[B22] Kawagoe R. Takikawa Y. Hikosaka O. (1998). Expectation of reward modulates cognitive signals in the basal ganglia. Nature Neurosci.1, 411–416. doi: 10.1038/1625 10196532

[B23] Kay K. Rokem A. Winawer J. Dougherty R. Wandell B. (2013). GLMdenoise: a fast, automated technique for denoising task-based fMRI data. Front. Neurosci. 7:54426. doi: 10.3389/fnins.2013.00247 24381539 PMC3865440

[B24] Kim S. Lee J. Lee I. (2012). The hippocampus is required for visually cued contextual response selection, but not for visual discrimination of contexts. Front. Behav. Neurosci. 6:66. doi: 10.3389/fnbeh.2012.00066 23060765 PMC3460488

[B25] Kraft D. (1988). A software package for sequential quadratic programming. Forschungsbericht- Deutsche Forschungs- und Versuchsanstalt fur Luft- und Raumfahrt.

[B26] Kwan A. Forbes P. A. Mitchell D. E. Blouin J.-S. Cullen K. E. (2019). Neural substrates, dynamics and thresholds of galvanic vestibular stimulation in the behaving primate. Nature Communications 10:1904. doi: 10.1038/s41467-019-09738-1 PMC6478681 31015434

[B27] Lee M. J. Kim S.-L. Lyoo C. H. Rinne J. O. Lee M.-S. (2015a). Impact of regional striatal dopaminergic function on kinematic parameters of Parkinson's disease. J. Neural Transmission 122, 669–677. doi: 10.1007/s00702-014-1296-x 25145816

[B28] Lee S. Kim D. J. Svenkeson D. Parras G. Oishi M. M. K. McKeown M. J. (2015b). Multifaceted effects of noisy galvanic vestibular stimulation on manual tracking behavior in parkinson's disease. Front. Systems Neurosci. 9:5. doi: 10.3389/fnsys.2015.00005 PMC4313776 25698944

[B29] Lee S. Smith P. F. Lee W. H. McKeown M. J. (2021). Frequency-specific effects of galvanic vestibular stimulation on response-time performance in Parkinson's disease. Front. Neurol. 12:758122. doi: 10.3389/fneur.2021.758122 PMC8593161 34795633

[B30] LeGates T. A. Kvarta M. D. Tooley J. R. Francis T. C. Lobo M. K. Creed M. C. . (2018). Reward behaviour is regulated by the strength of hippocampus-nucleus accumbens synapses. Nature 564, 258–262. doi: 10.1038/s41586-018-0740-8 30478293 PMC6292781

[B31] Liu A. Bi H. Li Y. Lee S. Cai J. Mi T. . (2021). Galvanic vestibular stimulation improves subnetwork interactions in parkinson's disease. J. Healthcare Eng. 2021:6632394. doi: 10.1155/2021/6632394 34094040 PMC8137296

[B32] MacDonald H. J. Fasmer O. B. Jønsi O. T. Sørensen L. (2025). Capturing trial-by-trial variability in behaviour: people with Parkinson's disease exhibit a greater rate of short-term fluctuations in response times. Transl. Psychiatry 15:300. doi: 10.1038/s41398-025-03516-y PMC12368238 40835602

[B33] Maitra R. (2010). A re-defined and generalized percent-overlap-of-activation measure for studies of fmri reproducibility and its use in identifying outlier activation maps. NeuroImage 50, 124–135. doi: 10.1016/j.neuroimage.2009.11.070 19963068

[B34] Mazzoni P. Hristova A. Krakauer J. W. (2007). Why don't we move faster? Parkinson's disease, movement vigor, and implicit motivation. J. Neurosci. 27, 7105–7116. doi: 10.1523/JNEUROSCI.0264-07.2007 PMC6794577 17611263

[B35] Mumford J. A. Turner B. O. Ashby F. G. Poldrack R. A. (2012). Deconvolving BOLD activation in event-related designs for multivoxel pattern classification analyses. Neuroimage 59, 2636–2643. doi: 10.1016/j.neuroimage.2011.08.076 21924359 PMC3251697

[B36] Niv Y. Daw N. D. Joel D. Dayan P. (2007). Tonic dopamine: opportunity costs and the control of response vigor. Psychopharmacology 191, 507–520. doi: 10.1007/s00213-006-0502-4 17031711

[B37] Nocedal J. Wright S. J. (2006). Numerical Optimization. Springer. doi: 10.1007/978-0-387-40065-5

[B38] Paton J. J. Belova M. A. Morrison S. E. Salzman C. D. (2006). The primate amygdala represents the positive and negative value of visual stimuli during learning. Nature 439, 865–870. doi: 10.1038/nature04490 PMC2396495 16482160

[B39] Pekny S. E. Izawa J. Shadmehr R. (2015). Reward-dependent modulation of movement variability. J. Neurosci. 35, 4015–4024. doi: 10.1523/JNEUROSCI.3244-14.2015 25740529 PMC4348194

[B40] Pierrieau E. Dussard C. Plantey-Veux A. Guerrini C. Lau B. Pillette L. . (2025). Changes in cortical beta power predict motor control flexibility, not vigor. Communications Biol. 8:1041. doi: 10.1038/s42003-025-08465-2 40640486 PMC12246413

[B41] Prince J. S. Charest I. Kurzawski J. W. Pyles J. A. Tarr M. J. Kay K. N. (2022). Improving the accuracy of single-trial fMRI response estimates using GLMsingle. elife 11:e77599. doi: 10.7554/eLife.77599 36444984 PMC9708069

[B42] Pruim R. H. Mennes M. van Rooij D. Llera A. Buitelaar J. K. Beckmann C. F. (2015). ICA-AROMA: A robust ICA-based strategy for removing motion artifacts from fMRI data. Neuroimage 112:267–277. doi: 10.1016/j.neuroimage.2015.02.064 25770991

[B43] Reppert T. R. Lempert K. M. Glimcher P. W. Shadmehr R. (2015). Modulation of saccade vigor during value-based decision making. J. Neurosci. 35, 15369–15378. doi: 10.1523/JNEUROSCI.2621-15.2015 26586823 PMC4649007

[B44] Rolls E. T. Cheng W. Feng J. (2020). The orbitofrontal cortex: reward, emotion and depression. Brain Communications 2:fcaa196. doi: 10.1093/braincomms/fcaa196 33364600 PMC7749795

[B45] Roweis S. (1997). EM algorithms for PCA and SPCA. Adv. Neural Informat. Proc. Syst. 10, 626–632.

[B46] Salimpour Y. Mari Z. K. Shadmehr R. (2015). Altering effort costs in Parkinson's disease with noninvasive cortical stimulation. J. Neurosci. 35, 12287–12302. doi: 10.1523/JNEUROSCI.1827-15.2015 26338339 PMC4556793

[B47] Sheridan M. R. Flowers K. A. (1990). Movement variability and bradykinesia in Parkinson's disease. Brain 113, 1149–1161. doi: 10.1093/brain/113.4.1149 2397387

[B48] Shirinbayan S. I. Dreyer A. M. Rieger J. W. (2019). Cortical and subcortical areas involved in the regulation of reach movement speed in the human brain: an fMRI study. Human Brain Mapping 40, 151–162. doi: 10.1002/hbm.24361 30251771 PMC6865438

[B49] Smith S. M. Jenkinson M. Woolrich M. W. Beckmann C. F. Behrens T. E. Johansen-Berg H. . (2004). Advances in functional and structural MR image analysis and implementation as FSL. Neuroimage 23, S208–S219. doi: 10.1016/j.neuroimage.2004.07.051 15501092

[B50] Spraker M. B. Yu H. Corcos D. M. Vaillancourt D. E. (2007). Role of individual basal ganglia nuclei in force amplitude generation. J. Neurophysiol. 98, 821–834. doi: 10.1152/jn.00239.2007 17567775 PMC2367092

[B51] Summerside E. M. Shadmehr R. Ahmed A. A. (2018). Vigor of reaching movements: reward discounts the cost of effort. J. Neurophysiol. 119, 2347–2357. doi: 10.1152/jn.00872.2017 29537911 PMC6734091

[B52] Therrien A. S. Wolpert D. M. Bastian A. J. (2016). Effective reinforcement learning following cerebellar damage requires a balance between exploration and motor noise. Brain 139, 101–114. doi: 10.1093/brain/awv329 PMC4949390 26626368

[B53] Turner R. S. Desmurget M. (2010). Basal ganglia contributions to motor control: a vigorous tutor. Curr. Opinion Neurobiol. 20, 704–716. doi: 10.1016/j.conb.2010.08.022 20850966 PMC3025075

[B54] Wallis J. D. (2007). Orbitofrontal cortex and its contribution to decision-making. Annual Rev. Neurosci. 30, 31–56. doi: 10.1146/annurev.neuro.30.051606.094334 17417936

[B55] Wilson J. M. Thompson C. K. McPherson L. M. Zadikoff C. Heckman C. MacKinnon C. D. (2020). Motor unit discharge variability is increased in mild-to-moderate Parkinson's disease. Front. Neurol. 11:477. doi: 10.3389/fneur.2020.00477 32547482 PMC7272659

[B56] Woolrich M. W. Behrens T. E. Beckmann C. F. Jenkinson M. Smith S. M. (2004). Multilevel linear modelling for fMRI group analysis using bayesian inference. Neuroimage 21, 1732–1747. doi: 10.1016/j.neuroimage.2003.12.023 15050594

[B57] Worsley K. J. (2001). “Statistical analysis of activation images,” in: Functional Magnetic Resonance Imaging: An Introduction to Methods, Jezzard, P., Matthews, P. M., and Smith, S. M., editors. (New York, NY: Oxford University Press), 251–270.

[B58] Wu H.-Y. Miyamoto Y. R. Gonzalez Castro L. N. Ölveczky B. P. Smith M. A. (2014). Temporal structure of motor variability is dynamically regulated and predicts motor learning ability. Nature Neurosci. 17, 312–321. doi: 10.1038/nn.3616 24413700 PMC4442489

[B59] Yewbrey R. Kornysheva K. (2024). The hippocampus preorders movements for skilled action sequences. J. Neurosci. 44:e0832242024. doi: 10.1523/JNEUROSCI.0832-24.2024 39317474 PMC11551893

[B60] Yttri E. A. Dudman J. T. (2016). Opponent and bidirectional control of movement velocity in the basal ganglia. Nature 533, 402–406. doi: 10.1038/nature17639 27135927 PMC4873380

[B61] Zénon A. Devesse S. Olivier E. (2016). Dopamine manipulation affects response vigor independently of opportunity cost. J. Neurosci. 36, 9516–9525. doi: 10.1523/JNEUROSCI.4467-15.2016 27629704 PMC6601940

