# Supplementary material for: A consistent vigor network in Parkinson's disease: a potential target for brain stimulation effects
Source: Front Hum Neurosci. 2026 Sep 30;20:1928519. doi: 10.3389/fnhum.2026.1928519 (PMC13662107; doi:10.3389/fnhum.2026.1928519)
Supplement: Supplementary file 1 [file Data_Sheet_1.pdf]

Supplementary Material

1 PARTICIPANT AND BEHAVIOURAL DETAILS

Table S1. Participant clinical, cognitive, affective, and laterality characteristics. Continuous measures are reported as mean ± SD with range; categorical measures are reported as counts.

Characteristic	Summary
Disease duration	2.00 ± 2.04 years (range 0–7; $n = 14$, incomplete, $n = 4$)
Prescribed levodopa equivalent daily dose (LEDD)	767.40 ± 551.06 mg/day (range 275.00–2693.25)
Motor phenotype	Tremor-dominant, $n = 8$; PIGD, $n = 10$
Cognitive screening	MoCA: 27.67 ± 1.81 (range 25–30)
Depression	BDI-II: 8.50 ± 8.26 (range 0–27)
Apathy	Apathy Scale: 11.28 ± 7.17 (range 3–27)
Derived upper-limb asymmetry	Right higher, $n = 8$; left higher, $n = 6$; tied, $n = 3$; incomplete, $n = 1$
Dominant hand	Right, $n = 14$; left, $n = 4$

Table S2. MDS-UPDRS Part III domain scores by medication state. Domain groupings are descriptive and are defined by the item numbers listed in the table. Values are reported as mean ± SD.

Domain	Items included	OFF mean ± SD	ON mean ± SD	ON–OFF mean ± SD
Axial signs	3.1, 3.2, 3.9-3.14	8.4 ± 4.6	7.3 ± 4.5	-1.2 ± 1.3
Rigidity	3.3A-3.3E	6.5 ± 3.9	5.8 ± 3.6	-0.7 ± 1.0
Limb bradykinesia	3.4A-3.8B	15.1 ± 7.6	11.3 ± 7.8	-3.8 ± 3.3
Tremor	3.15A-3.18	7.2 ± 5.3	3.6 ± 3.1	-3.6 ± 3.3

2 BEHAVIOURAL QUALITY CONTROL AND REACTION-TIME SUMMARIES

This section reports behavioural quality-control information. Figure S1 summarises trial-level reaction-time quality control by participant, medication session, and run.

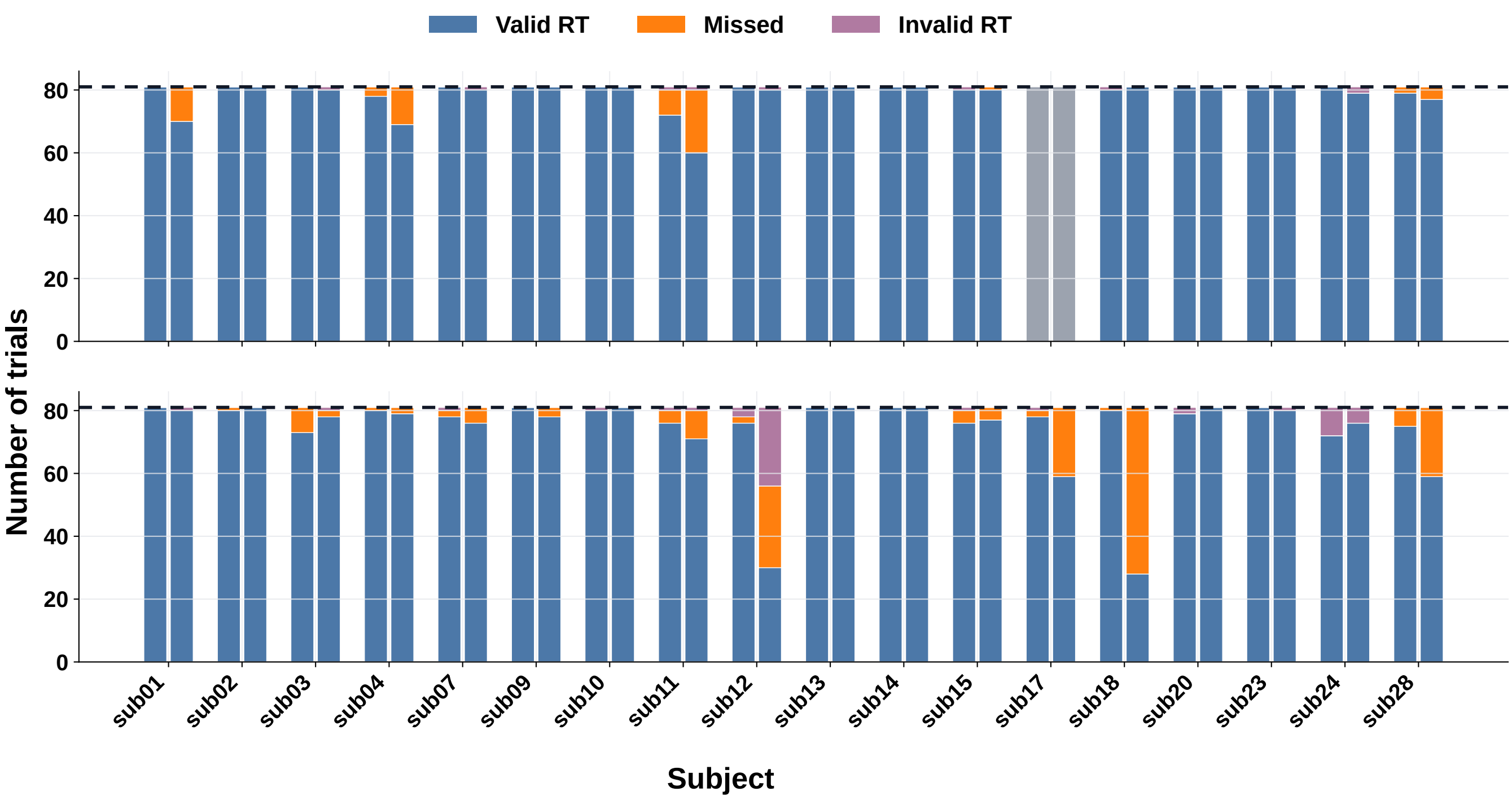

Figure S1: Reaction-time quality-control summary by participant, medication session, and run. Stacked bars show valid, missed, and invalid go trials; top and bottom panels show OFF- and ON-medication sessions, respectively. Within each participant, the left and right bars correspond to runs 1 and 2. The dashed horizontal line marks the expected number of go trials per run. Valid RT trials are go trials with finite positive RT values; missed trials are go trials flagged as no-squeeze events; invalid RT trials are go trials without finite RT values, indicating that the squeeze was not performed correctly. Grey bars mark run/session without corresponding fMRI data.

Figure S2 summarises reaction-time patterns across medication conditions and task progression. Reaction times are modestly faster in the ON-medication condition than in the OFF-medication condition. This pattern is visible both in the trial-wise RT distributions, where the ON distribution is slightly shifted toward shorter latencies, and in the subject-paired means, where most participants show lower mean RT in the ON-medication condition. The paired subject-level analysis is the analysis of record: the mean ON–OFF difference was -13.6 ms, with a 95% CI of $[-27.5, 0.3]$ ms ($t(16) = -2.08$, $p = 0.054$). This indicates a small medication-related speeding effect that approached but did not reach conventional significance. By comparison, temporal effects within the task are limited: RT is only slightly slower in run 2 than run 1, and trial position within blocks shows no clear monotonic practice or drift pattern. Overall, the behavioural results suggest a small medication-associated reduction in RT, with practice, fatigue, and within-task drift contributing only modestly to RT variation.

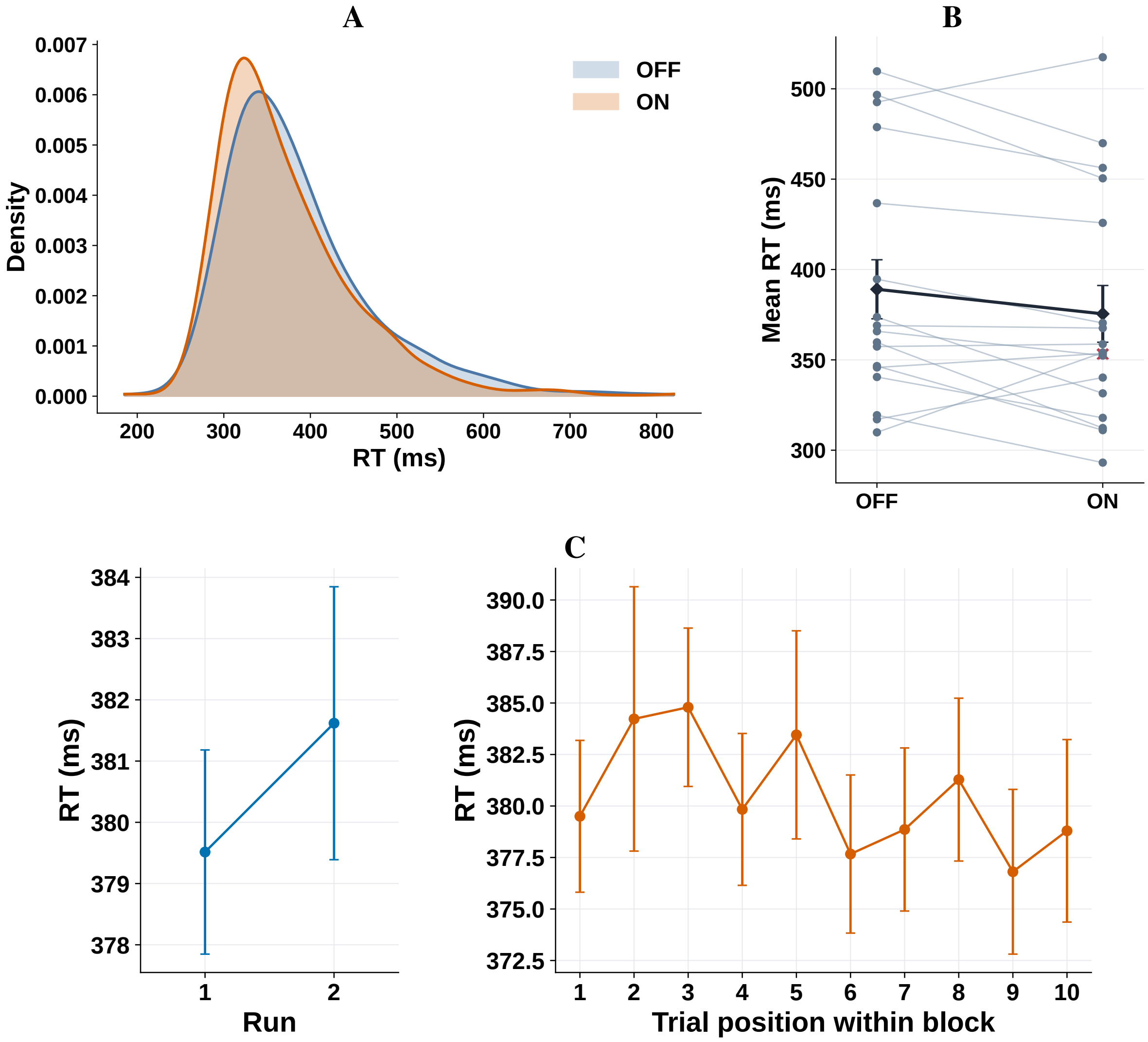

Figure S2: Supplementary behavioural reaction-time summaries. **A**, Trial-wise reaction-time distributions for OFF- and ON-medication sessions. **B**, Participant-level mean reaction times in the OFF- and ON-medication sessions. **C**, Mean reaction time arranged by run, and trial position within block.

Reward-related RT summaries are shown by participant in Figure S3. To examine whether reward value consistently influenced response initiation, we compared RT between low- and high-reward trials within each participant (Figure S3). The reward-related pattern is heterogeneous across participants. Some participants show shorter RTs on high-reward trials, consistent with greater behavioural vigour, whereas others show little difference or a small change in the opposite direction. Only a few participant-level comparisons reached significance. Thus, reward value modulates RT in some individuals, but it does not produce a uniform reward-related RT effect across the full sample.

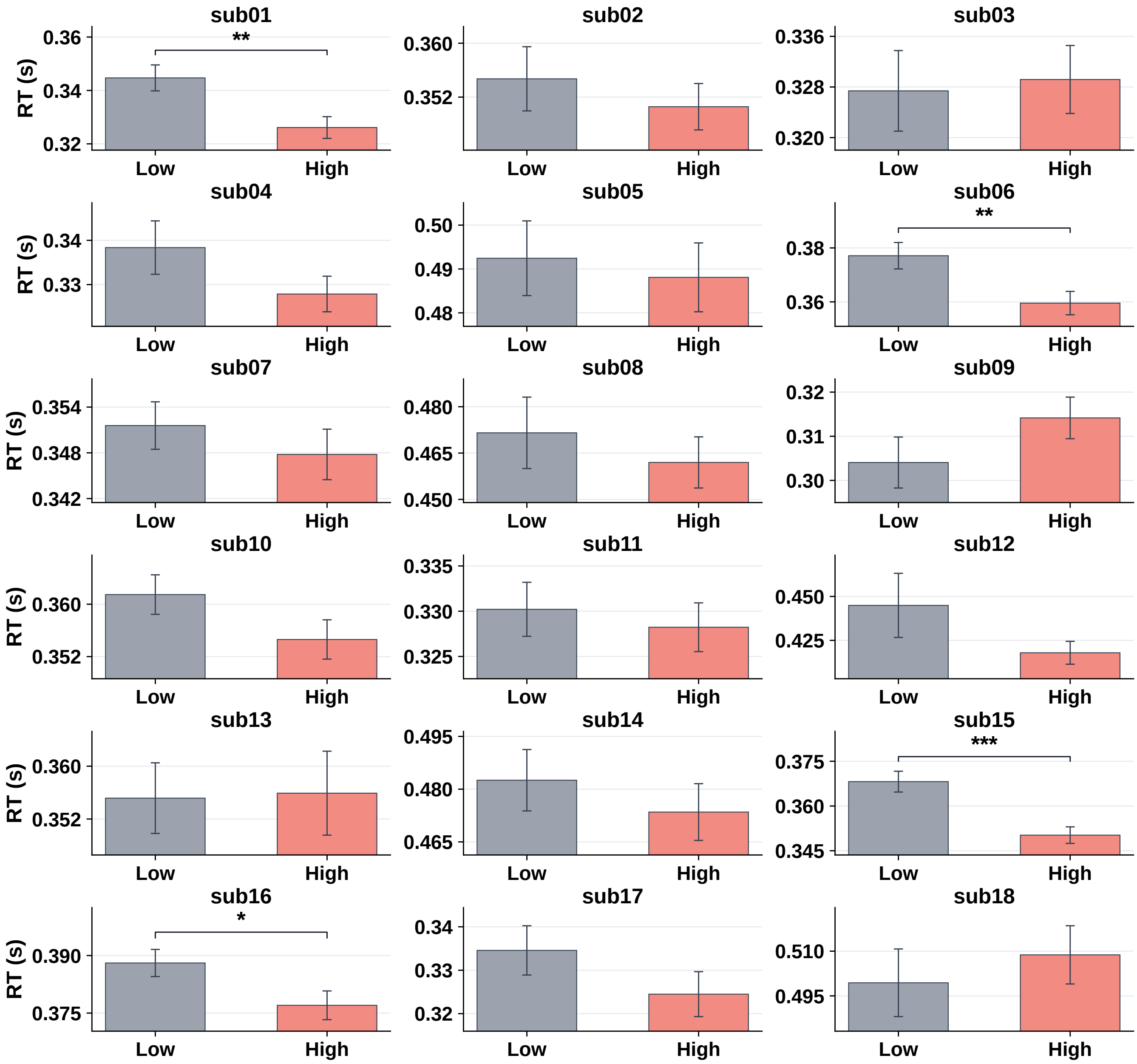

Figure S3: Reward-related reaction-time summaries by participant. Bars show mean reaction time for low- and high-reward trials within each participant. Error bars show the observed RT range, from minimum to maximum. Asterisks indicate significant Welch two-sample comparisons between high- and low-reward trials within each participant.

3 TASK-ACTIVATION MAP AND VIGOUR NETWORK COMPARISONS

Figure S4 shows the spatial distribution of the standard GLM from the group-level mixed-effects analysis. The thresholded map indicates widespread effects across posterior and lateral cortical regions, with the strongest clusters shown in yellow-orange.

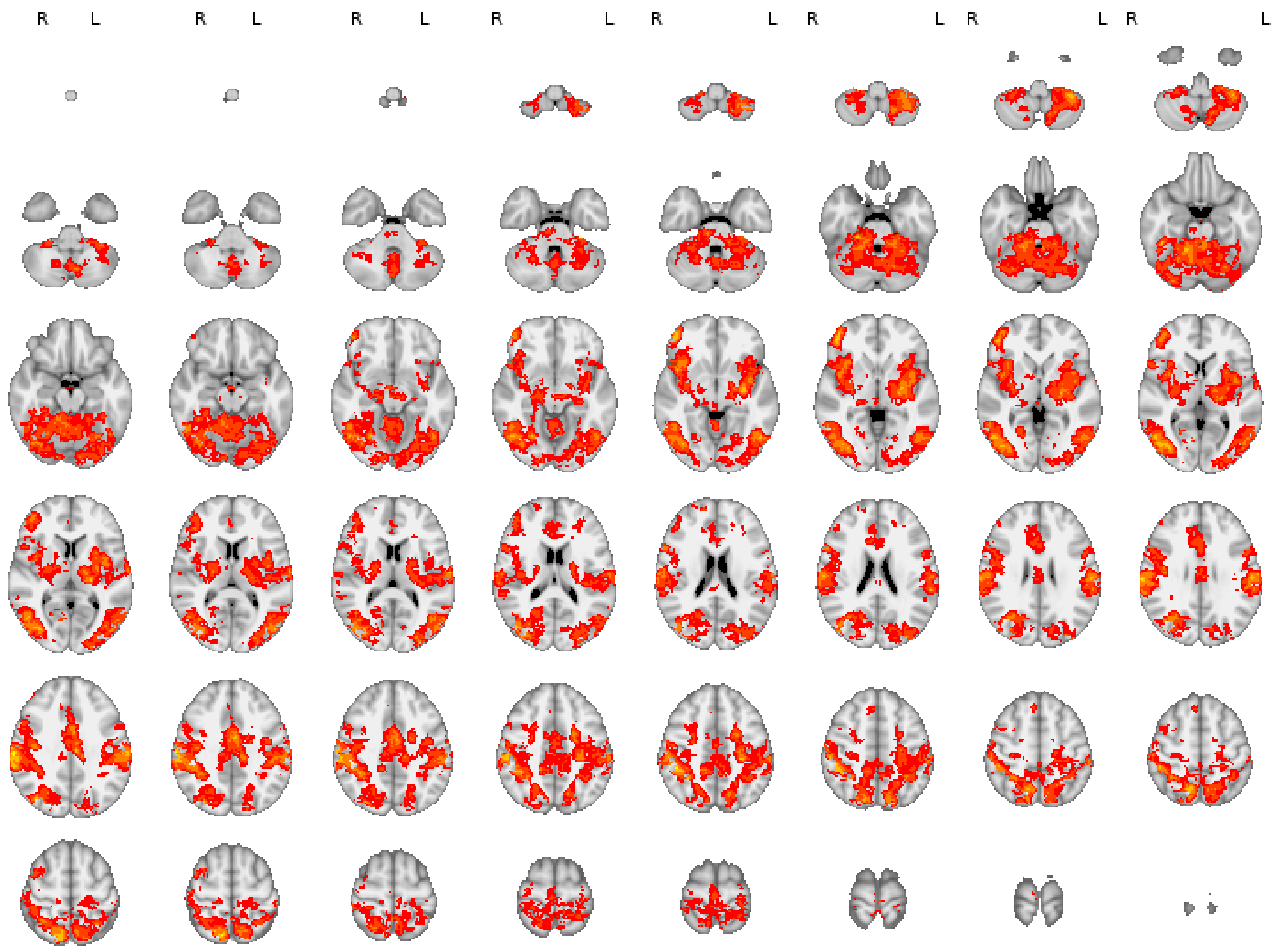

Figure S4: Group-level mixed-effects map. Axial brain slices show thresholded positive z-statistic values overlaid on the anatomical reference image. Warm colours represent suprathreshold values from the FEAT thresholded z-statistic image, with brighter colours reflecting higher z-values. The map is displayed at a cluster-forming threshold of $Z \geq 3.1$; R/L labels mark image orientation.

Spatial similarity between activation-derived maps and the vigour network is summarised below. Figure S5 compares the anatomical composition of the activation-derived maps with the vigour network. The standard GLM map and the two GLMsingle activation maps show broadly similar anatomical profiles, with major contributions from cerebellar, occipital, parietal, and frontal regions. Their pairwise Dice coefficients are moderate, indicating that these activation-derived maps capture overlapping task-related spatial patterns. In contrast, the vigour network shows low Dice overlap with each activation-derived map (standard GLM: Dice = 0.09; GLMsingle A: Dice = 0.11; GLMsingle D: Dice = 0.10). The vigour network also shows a different regional profile, with stronger relative representation in temporal and orbitofrontal regions and less dominance of the cerebellar and occipital regions seen in the activation-derived maps. This supports the interpretation that the vigour network is not simply a subset of general task activation, but reflects a more selective pattern related to the optimisation criteria used to identify the vigour projection.

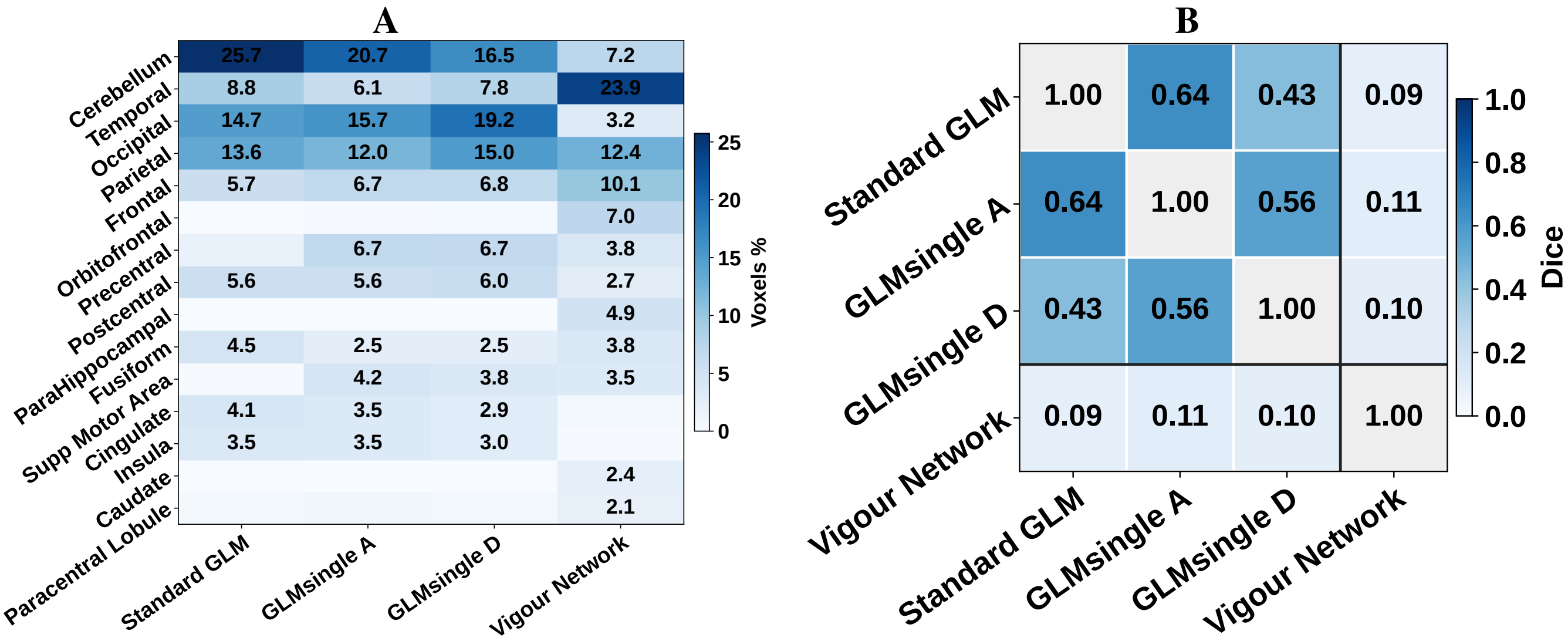

Figure S5: Spatial similarity between activation-derived maps and the vigour network. **A**, Regional composition of each map, expressed as the percentage of suprathreshold voxels assigned to each anatomical group. **B**, Dice coefficient matrix comparing the standard GLM map, two GLMsingle activation maps, and the vigour network.

4 ANATOMICAL CHARACTERISATION OF THE VIGOUR NETWORK

4.1 Threshold Robustness Analysis

We tested whether the definition of the vigour network depended strongly on the voxel-weight threshold. The threshold was varied across the voxel-weight distribution at p80, p85, p90, p95, and p97.5 (Figure S6). To summarise the anatomical composition of each thresholded map, suprathreshold voxels were assigned anatomical labels using the Automated Anatomical Labelling atlas 3, version 2 (AAL3v2; Rolls et al., 2020), and then grouped into broader bilateral anatomical regions. This grouping was applied only after thresholding and did not affect the voxel weights or the optimisation result.

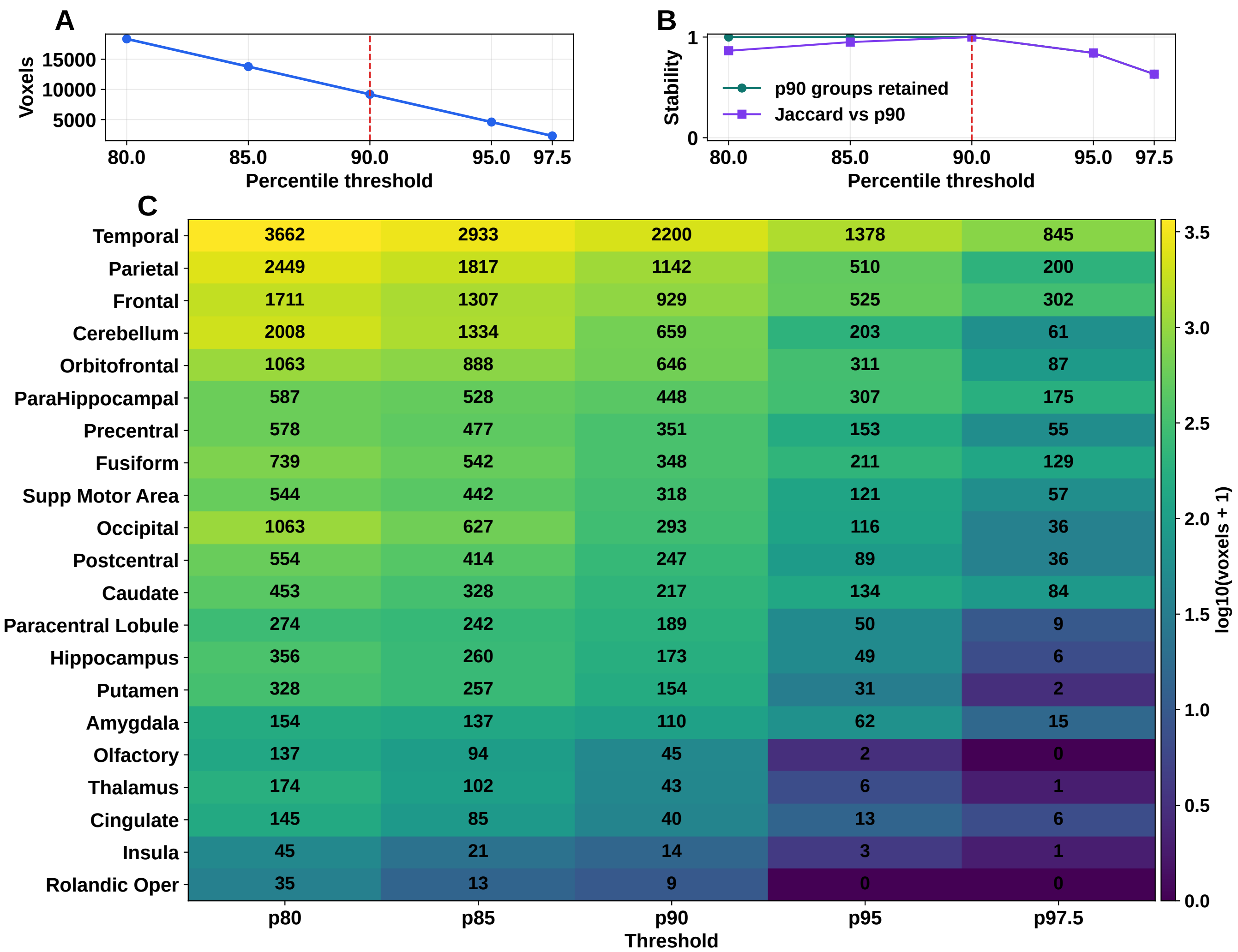

Figure S6: Threshold robustness analysis for the vigour network. The voxel-weight threshold was varied across percentiles p80, p85, p90, p95, and p97.5. (A) Number of retained voxels at each threshold. (B) Retention and Jaccard overlap of anatomical regions relative to the p90 reference set. (C) Anatomical composition of each thresholded map, showing voxel counts by region and threshold. Panel A includes all suprathreshold voxels in the threshold-robustness map, whereas panel C includes only voxels assigned to the anatomical groups displayed in the heat map.

Figure S6 reports the threshold-robustness counts from the full voxel-weight map. Panel A contains all suprathreshold voxels: 18,375 at p80, 13,782 at p85, 9,189 at p90, 4,595 at p95, and 2,297 at p97.5. Panel C is restricted to the anatomical groups displayed in the heat map and therefore sums to 17,059 voxels at p80 and 8,575 voxels at p90. The remaining voxels were atlas-unassigned or belonged to small atlas groups not displayed in panel C. The final ROI-wise table applies a further segmentation of the p90 vigour network into the predefined ROIs used for ROI-level analyses, retaining 8,182 of the 8,575 voxels shown in panel C. The 393-voxel difference therefore consists of panel-C voxels outside the final ROI masks. The p90 threshold provides a balance between reducing the voxel set and preserving the main anatomical pattern. All p90 anatomical groups are also present at p80 and p85, and the Jaccard overlap with the p90 region set remains high at p85 (0.95). In contrast, stricter thresholds begin to remove regions: p95 retains 84.2% of the p90 groups, whereas p97.5 retains only 63.2%.

4.2 ROI-wise composition of the vigour network

The AAL3-derived atlas shown in Figure S7 was used to define ROIs in this study. After thresholding the final voxel-weight map at the 90th percentile, suprathreshold voxels were assigned to AAL3-derived bilateral anatomical groups. These ROI labels were used only for anatomical description and ROI-level signal extraction; they did not affect the optimisation weights or the definition of the voxel-weight map.

The olfactory cortex label should be interpreted cautiously. In this atlas-based summary, it refers to the AAL3 anatomical region around the ventral/anterior frontal base and should not be taken as evidence for odour-related processing.

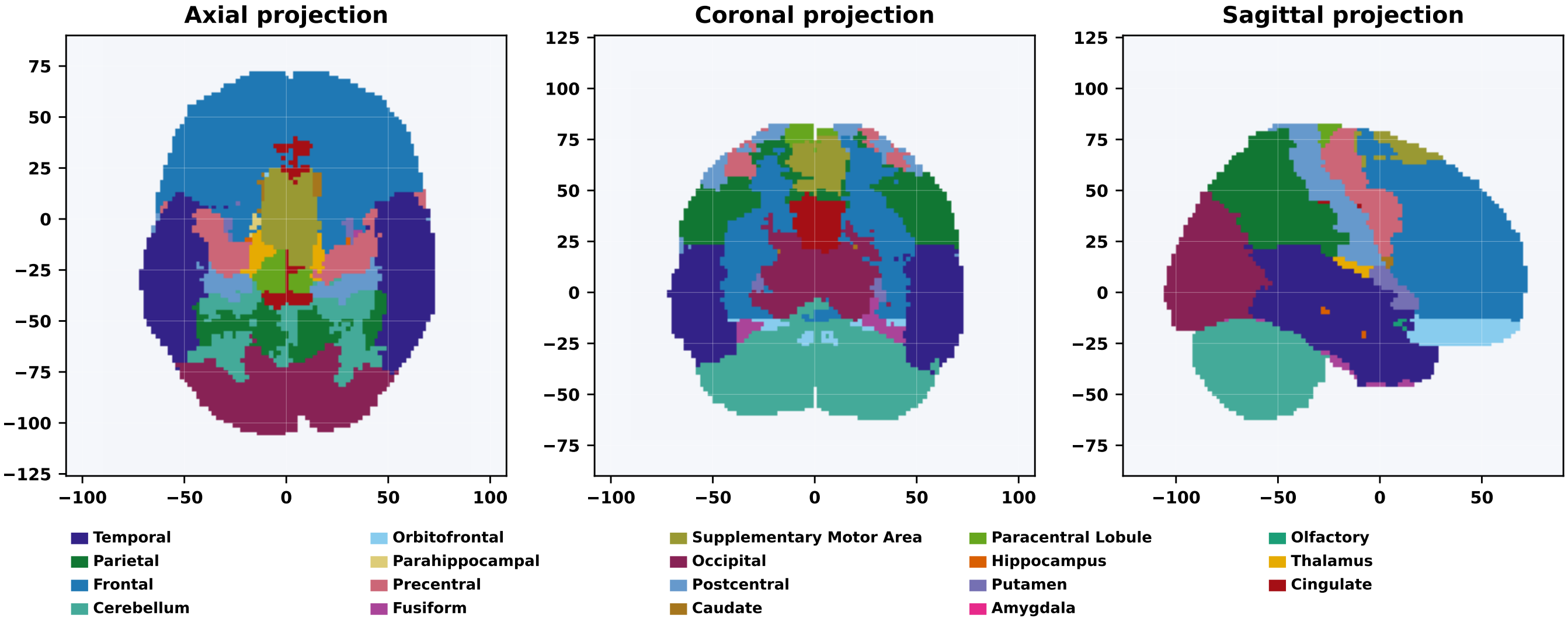

Figure S7: AAL3-derived bilateral ROI atlas used for anatomical summaries and ROI-level signal extraction. Axial, coronal, and sagittal projections show the grouped anatomical labels used in the network, medication-state, and connectivity analyses. Colours mark the broad bilateral ROI groups used for visualisation and summary statistics.

The vigour network is shown by the broad anatomical sector below. The ROI-wise profile shows that the vigour network is distributed across multiple anatomical systems rather than concentrated in a single motor region. In terms of total map composition, temporal cortex makes the largest contribution (25.27%), followed by parietal cortex (13.46%), frontal cortex (11.38%), cerebellum (7.90%), and orbitofrontal cortex (7.22%). These values describe how much each ROI contributes to the selected map, so larger anatomical regions can contribute a greater number of selected voxels.

To account for ROI size, we also examined the percentage of each ROI that was selected. This ROI-normalised view highlights stronger enrichment in temporal-limbic and subcortical regions, including the amygdala (26.52% of the ROI), parahippocampal gyrus (24.82%), caudate (14.27%), orbitofrontal cortex (12.60%), hippocampus (10.66%), paracentral lobule (10.46%), and putamen (10.32%). This pattern indicates that the vigour network is not simply driven by the largest cortical regions, but also includes smaller regions with relatively high selected-voxel coverage.

Motor-related regions are also clearly represented, including the supplementary motor area, precentral gyrus, postcentral gyrus, paracentral lobule, putamen, parietal cortex, and cerebellum. However, these regions do not dominate the ROI-normalised profile. Overall, the ROI-wise composition supports the view that the vigour network contains a motor-related component, but is broader than primary movement execution alone, extending into temporal-limbic, orbitofrontal, associative cortical, cerebellar, and subcortical regions.

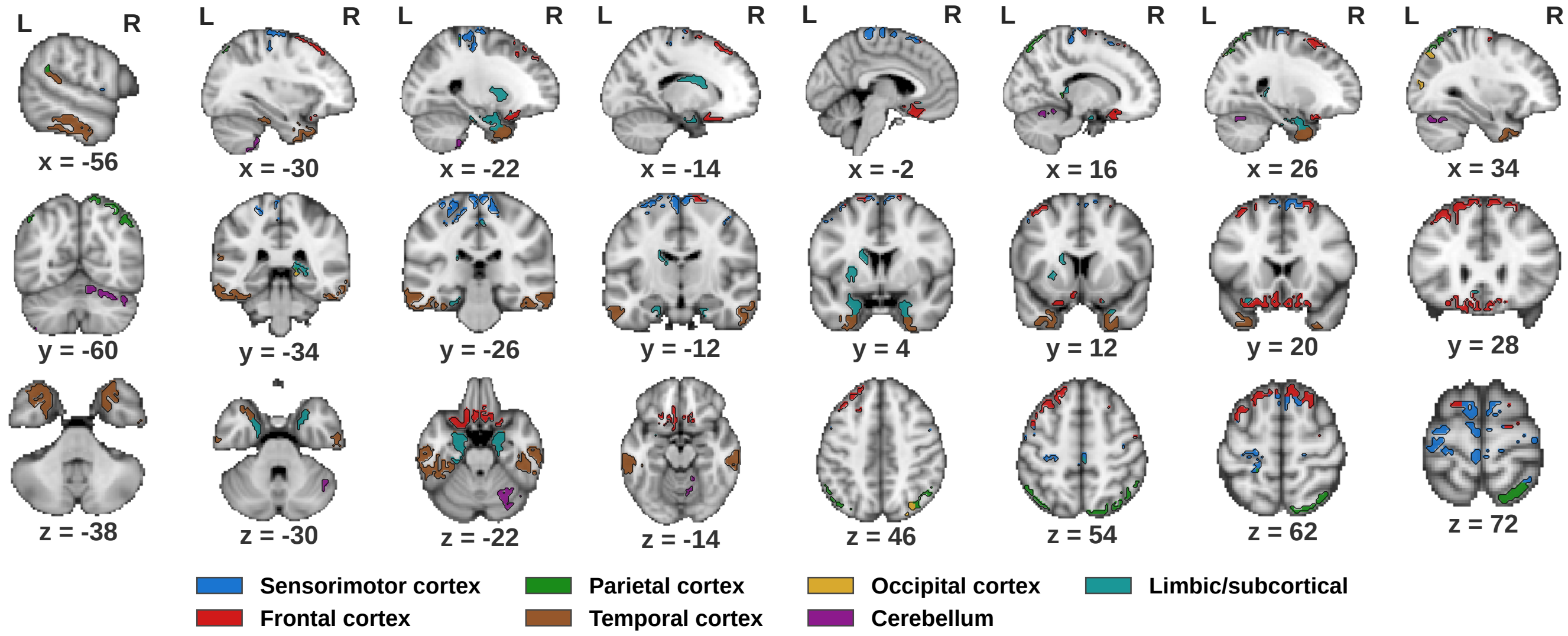

Figure S8: Anatomical-sector view of the vigour network. The thresholded voxel-weight map is overlaid on representative sagittal, coronal, and axial MNI slices and coloured by broad anatomical sector. The map includes sensorimotor, parietal, temporal, frontal, cerebellar, occipital, and limbic/subcortical components.

Table S3: ROI-wise composition of the suprathreshold vigour-related voxel-weight map shown in Fig. S8. ROIs are AAL3-derived bilateral anatomical groups and are ranked by their contribution to the selected map.

ROI	ROI size	Selected voxels	% ROI selected	% of map
Temporal cortex	23811	2,068	8.69	25.27
Parietal cortex	18024	1,101	6.11	13.46
Frontal cortex	29358	931	3.17	11.38
Cerebellum	22855	646	2.83	7.90
Orbitofrontal cortex	4689	591	12.60	7.22
Fusiform gyrus	4445	364	8.19	4.45
Parahippocampal gyrus	1378	342	24.82	4.18
Precentral gyrus	5632	339	6.02	4.14
Supplementary motor area	3394	310	9.13	3.79
Occipital cortex	18157	278	1.53	3.40
Postcentral gyrus	6867	237	3.45	2.90
Caudate	1205	172	14.27	2.10
Paracentral lobule	1615	169	10.46	2.07
Putamen	1492	154	10.32	1.88
Hippocampus	1088	116	10.66	1.42
Cerebral cortex	3610	113	3.13	1.38
Amygdala	411	109	26.52	1.33
Cingulate cortex	5668	38	0.67	0.46
Olfactory cortex	460	38	8.26	0.46
Thalamus	740	25	3.38	0.31
Nucleus accumbens	224	14	6.25	0.17
Insula	3529	13	0.37	0.16
Rolandic operculum	1892	9	0.48	0.11
Pallidum	523	5	0.96	0.06

ROI summaries confirm that the map includes motor and non-motor components (Table S3 and Fig. S8). Temporal cortex contributes 25.27% of selected voxels, parietal cortex 13.46%, frontal cortex 11.38%, cerebellum 7.90%, and orbitofrontal cortex 7.22%. After normalising by ROI size, stronger enrichment appears in temporal-limbic and striatal regions, including the parahippocampal gyrus, amygdala, caudate,

orbitofrontal cortex, and temporal cortex. Motor-related regions are also represented, including the supplementary motor area, paracentral lobule, precentral gyrus, parietal cortex, and cerebellum, but they do not dominate the ROI-normalised profile. Thus, the map contains a motor-related component, but its strongest ROI-level enrichment is distributed across temporal-limbic and associative regions rather than being confined to primary motor cortex.

4.3 Comparison with the task-activation map

Tables S4 and S5 provide a compact summary of the limited overlap between the vigour network and the task-activation map, including both global overlap metrics and region-wise voxel counts. The task-activation map serves as a reference for conventional task-evoked activity. This comparison asks whether the vigour network is simply a smaller subset of voxels that are active during the hand-squeeze task. Because task engagement is included in the optimisation framework, some overlap with the task-activation map is expected. However, task engagement is only one part of the model; the final vigour network is also shaped by behavioural relevance, low consecutive-trial variability, and spatial coherence.

The voxelwise comparison shows that the two maps have limited same-voxel overlap. The task-activation map contains 21,610 voxels, whereas the vigour network contains 8,182 voxels. Only 835 voxels are shared, corresponding to 3.86% of the task-activation map and 10.21% of the vigour network. This produces a Dice coefficient of 0.056 and a Jaccard index of 0.029. The shared voxels are mainly located in regions expected to support task performance, including the putamen, cerebellar lobules, middle occipital cortex, paracentral lobule, superior parietal cortex, and postcentral gyrus. Thus, the vigour network retains a task-related motor and sensorimotor component, but most of its selected voxels do not coincide with the task-activation map.

Table S4. Comparison of the vigour network and the task-activation map.

Network	Voxel count	Shared voxels (% of network)	Main anatomical labels
Task-activation map	21,610	835 (3.86%)	Cerebellum/vermis and thalamic nuclei; supramarginal cortex; occipital, parietal, and insular cortex
Vigour network	8,182	835 (10.21%)	Parahippocampal cortex; amygdala; middle temporal pole; inferior temporal cortex; rectus and medial/posterior orbitofrontal cortex

ROI metrics: voxelwise overlap = 835 voxels; Dice coefficient = 0.056; Jaccard index = 0.029.
The shared voxels are concentrated most in the putamen, cerebellar lobules 6/8, middle occipital cortex, paracentral lobule, superior parietal cortex, and postcentral gyrus.
In the GLM/GLMsingle comparison, the vigour network shows low overlap with each task-activation map: Dice = 0.09 with standard GLM, 0.11 with GLMsingle A, and 0.10 with GLMsingle D.

The ROI-level comparison further supports this separation. The task-activation map shows broad coverage of task-responsive regions, including cerebellar, occipital, parietal, postcentral, insular, and putaminal areas. In contrast, the vigour network shows stronger relative representation in temporal-limbic and associative regions, including parahippocampal, amygdala, orbitofrontal, temporal, caudate, and hippocampal regions. Some ROIs contain voxels from both maps but show little or no same-voxel overlap, indicating that the two maps can occupy the same broad anatomical region while selecting different local voxel patterns.

This distinction is also consistent with the activation-map comparison in Fig. S5. The standard GLM map and the two GLMsingle activation maps show moderate overlap with one another, whereas each shows low Dice overlap with the vigour network. Together, these results indicate that the vigour network is not simply a selection of the task-activation map. Rather, it reflects a more selective voxel pattern related to the optimisation criteria used to identify the vigour projection.

Table S5. ROI-level distribution of the vigour network, task-activation map, and their voxelwise overlap. The vigour network contains 8,182 voxels; the task-activation map contains 21,610 voxels. ROI rows report total voxels in each map within each ROI, including shared voxels; these rows sum to 8,182 vigour voxels, 21,610 task-activation voxels, and 835 common voxels.

ROI	Shared voxel n (% ROI)	Vigour network n (% ROI)	Task-activation map n (% ROI)	Voxelwise relation
Putamen	118 (7.91%)	154 (10.32%)	710 (47.59%)	Shared voxels present
Pallidum	1 (0.19%)	5 (0.96%)	171 (32.70%)	Shared voxels present
Fusiform	28 (0.63%)	364 (8.19%)	979 (22.02%)	Shared voxels present
Parietal	181 (1.00%)	1,101 (6.11%)	4,352 (24.15%)	Shared voxels present
Postcentral	81 (1.18%)	237 (3.45%)	1,679 (24.45%)	Shared voxels present
Amygdala	0 (0.00%)	109 (26.52%)	0 (0.00%)	Vigour network only in ROI
Insula	0 (0.00%)	13 (0.37%)	908 (25.73%)	Both maps in ROI, no shared voxels
Parahippocampal	0 (0.00%)	342 (24.82%)	5 (0.36%)	Both maps in ROI, no shared voxels
Occipital	86 (0.47%)	278 (1.53%)	3,538 (19.49%)	Shared voxels present
Cerebellum	233 (1.02%)	646 (2.83%)	4,259 (18.63%)	Shared voxels present
Temporal	43 (0.18%)	2,068 (8.69%)	2,015 (8.46%)	Shared voxels present
Paracentral_Lobule	19 (1.18%)	169 (10.46%)	122 (7.55%)	Shared voxels present
Caudate	0 (0.00%)	172 (14.27%)	0 (0.00%)	Vigour network only in ROI
Precentral	18 (0.32%)	339 (6.02%)	435 (7.72%)	Shared voxels present
Cingulate	5 (0.09%)	38 (0.67%)	724 (12.77%)	Shared voxels present
Hippocampus	0 (0.00%)	116 (10.66%)	29 (2.67%)	Both maps in ROI, no shared voxels
Rolandic_Oper	0 (0.00%)	9 (0.48%)	239 (12.63%)	Both maps in ROI, no shared voxels
Orbitofrontal	0 (0.00%)	591 (12.60%)	15 (0.32%)	Both maps in ROI, no shared voxels
Thalamus	0 (0.00%)	25 (3.38%)	57 (7.70%)	Both maps in ROI, no shared voxels
Supp_Motor_Area	2 (0.06%)	310 (9.13%)	43 (1.27%)	Shared voxels present
Olfactory	0 (0.00%)	38 (8.26%)	0 (0.00%)	Vigour network only in ROI
Frontal	19 (0.06%)	931 (3.17%)	1,258 (4.29%)	Shared voxels present
N_Acc	0 (0.00%)	14 (6.25%)	0 (0.00%)	Vigour network only in ROI
Cerebral_Cortex	1 (0.03%)	113 (3.13%)	72 (1.99%)	Shared voxels present

5 DETAILED EXPERIMENTAL AND PREPROCESSING METHODS

This section contains procedural details.

5.1 Full galvanic vestibular stimulation protocol

Galvanic vestibular stimulation (GVS) was delivered continuously throughout each run using an STMISOLA constant-current stimulator (BIOPAC Systems Inc.) through 41-mm Ag/AgCl electrodes placed bilaterally over the mastoid processes. Stimulation began before the first trial of the run. Electrode placement was verified using a 1 Hz sinusoidal stimulus. Stimulation amplitude was then individualised using a staircase procedure with pink-noise GVS (Nguyen et al., 2022). Pink-noise current began at 0.10 mA and was increased in 0.10 mA steps until the participant reported a superficial cutaneous sensation, such as tingling or pricking, at the electrode site. The current was then decreased in 0.05 mA steps until the sensation was no longer perceived. This final current setting was used for all eight active GVS waveforms in the corresponding session.

Usable threshold records were available for 16 of 18 participants. Values are reported as mean $\pm$ SD. The pink-noise-derived peak current used for active stimulation was 0.70 ± 0.44 mA (range 0.20-1.65 mA) in the OFF-medication session and 0.68 ± 0.43 mA (range 0.20-1.65 mA) in the ON-medication session. The corresponding 1-Hz electrode-verification thresholds were 0.55 ± 0.37 mA (range 0.20-1.50 mA) and 0.57 ± 0.36 mA (range 0.20-1.40 mA), respectively. Stimulation was well tolerated. At these individually determined subthreshold amplitudes, participants reported no vestibular sensation during any active waveform. Participants were blinded to block condition and could not distinguish active from sham stimulation. Participants were monitored throughout stimulation for discomfort or adverse effects.

Eight active amplitude-modulated waveforms were generated in MATLAB using suppressed-carrier modulation. The waveforms varied in carrier frequency (75 or 125 Hz) and envelope frequency (0, 10, 16, or 25 Hz). The resulting GVS stimuli were labelled as follows: GVS1, 75 Hz carrier and 0 Hz envelope; GVS2, 75 Hz carrier and 10 Hz envelope; GVS3, 75 Hz carrier and 16 Hz envelope; GVS4, 75 Hz carrier and 25 Hz envelope; GVS5, 125 Hz carrier and 0 Hz envelope; GVS6, 125 Hz carrier and 10 Hz envelope; GVS7, 125 Hz carrier and 16 Hz envelope; and GVS8, 125 Hz carrier and 25 Hz envelope. A sham condition, in which no current was delivered (0 mA), was also included. These frequencies were selected to approximate typical vestibular afferent firing rates and oscillatory bands implicated in motor planning and execution. All stimulus waveforms were generated with MATLAB's 'ammod' function using zero initial phase and zero carrier amplitude, producing suppressed-carrier amplitude modulation. Spectral analysis confirmed the expected frequency structure: for the modulated waveforms, the spectrum contained two symmetric sideband peaks around the carrier, located at $f_c \pm f_e$, where f_c is the carrier frequency and f_e is the envelope frequency.

Each run was divided into nine blocks, with every block corresponding to a single stimulation condition: one of the eight active GVS waveforms or the sham condition. Each block contained 10 trials, and the assigned stimulation condition was active throughout the full block, including the cue period, delay period, Go-signal period, and response period. All nine conditions were presented exactly once per run. At block transitions, the next stimulation condition began immediately, except after every third block, when a 20-s rest period was provided before the next condition. The order of the nine blocks was randomised independently for each run, so that the sequence of conditions differed across runs, sessions, and subjects. Participants were not informed of the stimulation order. Because stimulation conditions were presented in a blocked sequence without a dedicated washout interval between all adjacent blocks, carryover effects between successive stimulation conditions cannot be fully excluded. Randomisation of block order was used to reduce systematic order effects, and GVS-related results were therefore interpreted cautiously.

5.2 Full MRI acquisition and preprocessing protocol

Structural and functional MRI data were acquired on a Philips 3T Ingenia Elition X whole-body MRI scanner upgraded to an MR7700 system (Philips Healthcare, Best, The Netherlands) using a 32-channel

head coil. For each participant, a T1-weighted structural image was acquired, followed by two task-based fMRI scans, one for each run of the behavioural task.

T1-weighted images were acquired using a 3D MPRAGE sequence with the following parameters: flip angle = $8°$, echo time (TE) = 3.6 ms, repetition time/shot interval (TR) = 2400 ms, inversion time = 950 ms, 180 slices, field of view (FOV) = $256 \times 256 \times 180$ mm^3, and spatial resolution = 1 mm^3 isotropic. Images were acquired with a compressed SENSE factor of 3.6, and the acquisition time was 3:42 min.

Functional MRI data were acquired using echo-planar imaging (EPI). The functional acquisition protocol differed for the first three participants compared with the rest of the sample. The first three participants were scanned with a multi-echo EPI protocol, with flip angle = $77°$, TE = 12 ms, TR = 2000 ms, 48 slices, FOV = $240 \times 144 \times 240$ mm^3, spatial resolution = 3 mm^3 isotropic, SENSE factor = 2, multiband factor = 2, and acquisition time = 14:22 min. The remaining participants were scanned with a single-echo EPI protocol, with flip angle = $77°$, TE = 30 ms, TR = 1000 ms, 51 slices, FOV = $240 \times 153 \times 240$ mm^3, spatial resolution = 3 mm^3 isotropic, SENSE factor = 2, multiband factor = 3, and acquisition time = 14:17 min. To support distortion correction, two spin-echo EPI images were acquired with opposite fat-shift directions, anterior-posterior and posterior-anterior. These spin-echo images were acquired with flip angle = $77°$, TE = 55 ms, TR = 2650 ms, 51 slices, FOV = $240 \times 153 \times 240$ mm^3, spatial resolution = 3 mm^3 isotropic, SENSE factor = 2, multiband factor = 3, and acquisition time = 5.3 s.

Image preprocessing was performed in FSL (Smith et al., 2004). Structural images underwent bias-field correction and brain extraction. Functional images were corrected for head motion and susceptibility-induced distortions, with distortion correction referring to the correction of susceptibility-related spatial distortions in the functional images using the spin-echo images acquired with opposite fat-shift directions. No initial functional volumes were discarded during preprocessing because the scanner automatically discarded four dummy volumes before the start of saved image acquisition. Functional images were then high-pass filtered using a 100 s cutoff, brain extracted, denoised using ICA-AROMA (Pruim et al., 2015), and spatially smoothed with a 5 mm full-width at half-maximum Gaussian kernel. The preprocessed functional images were then registered to each participant's structural image and normalised to standard MNI152 space.

5.3 Task-activation map

To provide a brain map of task-evoked activation, we first ran a standard GLM using FSL FEAT version 6.00 (Jenkinson et al., 2012). This analysis is separate from the trial-wise GLM described below and is used only to generate the task-activation map shown in Supplementary Figure S4.

For each run, all valid Go trials (Supplementary Figure S1) were modelled with one event regressor. Event timing was defined relative to Go-signal onset, with a duration of 2 s and an amplitude of 1. This regressor was convolved with FSL's double-gamma hemodynamic response function, and a temporal derivative was included.

The first-level contrast tested the positive effect of task activity. The two runs from each subject-session were then combined using a fixed-effects model. The resulting subject-session maps were entered into a higher-level mixed-effects model using FLAME stage 1 (Woolrich et al., 2004; Beckmann et al., 2003). The group model included one mean regressor and tested whether the average task response was greater than zero across the 35 available subject-sessions. Group Z-statistic maps were thresholded using clusters formed at $Z \geq 3.1$ with a corrected cluster significance threshold of $p \leq 0.05$ (Worsley, 2001). The thresholded group map was used as the task-activation map (Supplementary Figure S4).

5.4 Trial-wise GLM estimation and beta preprocessing details

Task-related BOLD responses for each trial were estimated using trial-wise general linear model (GLM) beta estimates with GLMsingle (Prince et al., 2022), with HRF fitting, GLMdenoise (Kay et al., 2013), and fractional ridge regression enabled. Analyses were performed separately for each subject-session. Within each subject-session, the two task runs were analysed jointly, while run identity was preserved in the design matrix and nuisance regressors.

The GLM design matrix included Go-signal onset regressors with a fixed trial duration of 2 s, corresponding to the period during which participants performed the task. Catch trials were not modelled

as separate regressors and were excluded from the analysis because participants did not squeeze the bulb on these trials (630 of $6{,}300$ trials across all datasets). Reward-condition labels were also not included in the GLM design. To restrict the analysis to relevant tissue, background, cerebrospinal fluid (CSF), and white-matter voxels were masked out, retaining only grey-matter voxels. The initial MNI grey-matter, non-CSF candidate mask contained $91{,}781$ voxels. The mean CSF time series for each run was included as a nuisance regressor.

HRF fitting used the default GLMsingle library of 20 candidate HRFs, and the HRF yielding the highest variance explained was selected separately for each voxel. GLMdenoise derived additional nuisance regressors from low task-related-variance voxels using principal component analysis and selected the number of noise components by cross-validation. Fractional ridge regression was used to stabilise single-trial beta estimates. Shrinkage fractions from 0.05 to 1.00 in increments of 0.05 were evaluated, and the optimal shrinkage level was selected separately for each voxel by cross-validation. Run-specific baseline terms were modelled using GLMsingle's run-wise polynomial regressors, including degree-0 intercepts and higher-order polynomial terms up to degree $\mathrm{round}(L/2)$, where L is the run length in minutes.

A second cleaning step was then applied to the GLMsingle beta estimates. To detect outlier beta values, beta estimates were robustly normalised within each subject-session using the session-level median and MAD:

$$\beta_{\mathrm{norm}} = \frac{\beta - \mathrm{median}(\beta)}{\mathrm{MAD}(\beta)}.$$

Beta values with $|\beta_{\mathrm{norm}}|$ above the 99.9th percentile were flagged as beta outliers. For each voxel, the beta-outlier fraction was computed as the proportion of trials flagged as outliers. Voxels were retained when this fraction was ≤ 0.5 and excluded when more than 50% of their trials were beta outliers. This majority-trial threshold was used to remove voxels with persistently unstable beta estimates, rather than voxels with occasional large responses.

This cleaning retained $91{,}701 \pm 54$ voxels on average. This corresponded to an average removal of $0.09\% \pm 0.06\%$. To assess whether this filtering disproportionately affected susceptibility-prone ventral regions, removal rates were summarised using atlas labels and ventral-position proxies. Average run-level removal was 0.20% in frontal orbital cortex, 0.06% in temporal pole, 0.76% in inferior temporal gyrus, 0.25% in the ventral bottom quartile, and 0.03% in all other voxels. Thus, run-level cleaning removed very few voxels overall; removal was somewhat higher in inferior temporal and ventral-bottom voxels, but absolute removal rates remained below 1% in all regions.

The cleaned outputs from all subjects, sessions, and runs were then used to define a shared voxel space for group-level analysis. Before pooling, the single-trial beta and active-task BOLD data were normalised separately within each subject, medication session, and run by subtracting the run mean and dividing by the mean absolute deviation from that mean. The coverage threshold was applied across all concatenated trial observations from all subjects, sessions, and runs, rather than separately within each subject or session. This approach was used because the group-level analysis required a common MNI-space voxel feature set across trials. Applying separate subject-level masks would have produced different voxel sets across subjects, whereas the shared mask allowed all valid trial-wise beta estimates to be aligned into one group-level matrix. The threshold was also permissive: voxels were retained if they had finite beta estimates for at least 70% of all trial observations, so a voxel was not excluded simply because it was missing or unstable in a minority of subjects, sessions, or runs.

The final group coverage mask contained $91{,}769$ voxels, yielding a final group beta matrix of $91{,}769$ voxels $\times$ $5{,}463$ retained trials (Supplementary Figure S1). The filter excluded 12 voxels from the $91{,}781$-voxel candidate mask, retaining 99.99% of candidate voxels. Subsequent analyses used the concatenated cleaned beta data in this final group voxel space.

6 OPTIMISATION DERIVATION AND MODEL-SELECTION DETAILS

A group-level optimisation procedure identifies a vigour network (main Figure 1B, C). The aim of the optimisation framework is to identify a voxel-weight vector that produces a projected neural signal related

to trial-wise motor vigour while satisfying constraints on task engagement, spatial coherence, and low consecutive-trial variability. In voxel space, this projection is defined by a voxel-weight vector, $\mathbf{w}^{\mathrm{org}}$, that combines voxel activity into one scalar signal at each sample. For a neural data matrix $\mathbf{X}^{\mathrm{org}} \in \mathbb{R}^{N_v \times N_s}$, where columns are samples, the projected signal is

$$\mathbf{y}^{\mathrm{org}} = (\mathbf{w}^{\mathrm{org}})^\top \mathbf{X}^{\mathrm{org}}, \qquad \mathbf{w}^{\mathrm{org}} \in \mathbb{R}^{N_v \times 1}, \mathbf{y}^{\mathrm{org}} \in \mathbb{R}^{1 \times N_s}.$$

Here, N_v is the number of voxels and N_s denotes the number of time samples in the neural data matrix. In this study, the same projection can be applied to two forms of neural data: single-trial beta estimates and BOLD time courses:

$$\mathbf{y}^{\mathrm{org}}_\beta = (\mathbf{w}^{\mathrm{org}})^\top \mathbf{X}^{\mathrm{org}}_\beta, \qquad \mathbf{X}^{\mathrm{org}}_\beta \in \mathbb{R}^{N_v \times N_{\mathrm{tr}}}, \mathbf{y}^{\mathrm{org}}_\beta \in \mathbb{R}^{1 \times N_{\mathrm{tr}}}.$$

$$\mathbf{y}^{\mathrm{org}}_{\mathrm{BOLD}} = (\mathbf{w}^{\mathrm{org}})^\top \mathbf{X}^{\mathrm{org}}_{\mathrm{BOLD}}, \qquad \mathbf{X}^{\mathrm{org}}_{\mathrm{BOLD}} \in \mathbb{R}^{N_v \times N_{\mathrm{tp}}}, \mathbf{y}^{\mathrm{org}}_{\mathrm{BOLD}} \in \mathbb{R}^{1 \times N_{\mathrm{tp}}}.$$

Here, N_{tr} is the number of trials, and N_{tp} is the number of time samples. $\mathbf{X}^{\mathrm{org}}_\beta$ and $\mathbf{X}^{\mathrm{org}}_{\mathrm{BOLD}}$ are not separate models; they are two representations of the neural data used by the same projection framework.

Although the framework is defined in voxel space, direct optimisation over all voxels is impractical because of the high dimensionality of the data and memory limitations. In addition, the task is not a simple motor task and includes several task-related and non-motor processes. Therefore, reducing components that are unlikely to reflect the motor-vigour-related signal is important before optimisation. Because missing values remained after beta-estimate preprocessing, the neural data were reduced using expectation-maximisation principal component analysis (EMPCA) (Roweis, 1997). An 80% cumulative explained-variance threshold initially retained 14 components.

EMPCA provides voxel-to-PC weights, $\mathbf{W}_{\mathrm{pca}} \in \mathbb{R}^{N_v \times N_{\mathrm{pc}}}$. Both neural data matrices are projected into this common PCA space:

$$\mathbf{X}_\beta = \mathbf{W}^\top_{\mathrm{pca}} \mathbf{X}^{\mathrm{org}}_\beta, \qquad \mathbf{X}_\beta \in \mathbb{R}^{N_{\mathrm{pc}} \times N_{\mathrm{tr}}},$$
$$\mathbf{X}_{\mathrm{BOLD}} = \mathbf{W}^\top_{\mathrm{pca}} \mathbf{X}^{\mathrm{org}}_{\mathrm{BOLD}}, \quad \mathbf{X}_{\mathrm{BOLD}} \in \mathbb{R}^{N_{\mathrm{pc}} \times N_{\mathrm{tp}}}.$$

The optimisation is then performed in PCA space using $\mathbf{w} \in \mathbb{R}^{N_{\mathrm{pc}} \times 1}$. In this space, the projected beta and BOLD signals are

$$\mathbf{y}_\beta = \mathbf{w}^\top \mathbf{X}_\beta, \qquad \mathbf{y}_\beta \in \mathbb{R}^{1 \times N_{\mathrm{tr}}},$$
$$\mathbf{y}_{\mathrm{BOLD}} = \mathbf{w}^\top \mathbf{X}_{\mathrm{BOLD}}, \quad \mathbf{y}_{\mathrm{BOLD}} \in \mathbb{R}^{1 \times N_{\mathrm{tp}}}.$$

The total loss is defined using these two projected signals: the beta projection is used for the trial-wise beta-based terms, including behavioural relevance to motor vigour, and the BOLD projection is used for the consecutive-trial variability term. After optimisation, the PCA-space weights are projected back to voxel space as $\mathbf{w}^{\mathrm{org}} = \mathbf{W}_{\mathrm{pca}} \mathbf{w}$. The resulting voxel-space weight vector is used to define the vigour network.

The optimisation model is based on four assumptions about the neural representation of motor vigour. First, the vigour network should consist of voxels engaged during task performance. Second, the projected signal should encode behavioural information. Third, neighbouring voxels should show similar contributions, reflecting spatial coherence. Fourth, the selected signal should show low consecutive-trial variability. These four conditions are incorporated into the model one by one, as described below.

Condition 1: The projected signal should come from task-engaged voxels.

The first condition is that the projected signal should be generated mainly from voxels that are engaged during task performance. Each voxel contributes to y_β through its trial-wise beta activity and

its corresponding weight in w. Therefore, assigning a large weight to a voxel with weak task-related beta-response magnitude should be penalised, whereas voxels with stronger task-related beta responses should be penalised less.

For each voxel j, we define the voxel-wise beta-response magnitude across trials as

$$a_j = \|X_{\beta,j:}\|_2 = \left(\sum_{t=1}^{n_{\text{trials}}} X_{\beta,jt}^2\right)^{1/2},$$

where $X_{\beta,j:}$ denotes the row of X_β corresponding to voxel j. Collecting these values gives

$$\mathbf{a} = (a_1, \ldots, a_{n_{\text{voxels}}})^\top.$$

The task-engagement penalty is then written as

$$L_{\text{task}}(w) = w^\top C_{\text{task}} w, \quad C_{\text{task}} = [\text{diag}(\mathbf{a} + \epsilon \mathbf{1})]^{-1}.$$

Thus, C_{task} favours voxels with stronger task-related beta-response magnitude and discourages the model from selecting voxels with weak task engagement.

Condition 2: The projected signal should encode behavioural information.

The second condition is that the projected neural signal should be behaviourally relevant. The behavioural variable was inverse RT. Within each cross-validation training fold, inverse RT and the EMPCA-derived beta scores were mean-centred, and inverse RT was scaled to unit norm. Inverse RT was not additionally z-scored within subjects. Therefore, the model should find w such that y is strongly associated with the behavioural variable X_{behav}:

$$\begin{aligned}
\max_w \text{Corr}\big(X_{\text{behav}}, y_\beta\big)^2 &= \max_w \text{Corr}\Big(X_{\text{behav}}, w^\top X_\beta\Big)^2 \\
&= \max_w \frac{\big[(w^\top X_\beta) X_{\text{behav}}^\top\big]^2}{w^\top (X_\beta X_\beta^\top) w} \\
&= \max_w \frac{w^\top \Big(X_\beta X_{\text{behav}}^\top X_{\text{behav}} X_\beta^\top\Big) w}{w^\top \Big(X_\beta X_\beta^\top\Big) w} \\
&= \max_w \frac{w^\top C_{RT} w}{w^\top C_{var} w},
\end{aligned}$$

where

$$C_{RT} = X_\beta X_{\text{behav}}^\top X_{\text{behav}} X_\beta^\top, \qquad C_{var} = X_\beta X_\beta^\top.$$

Here, C_{RT} captures the component of the projected beta signal that is aligned with behaviour, while C_{var} normalises the association by the variance of the projected beta signal. Since the final optimisation problem is written as a minimisation problem, this behavioural-relevance term is later included with a negative sign.

Condition 3: The projected signal should come from a spatially coherent network.

The third condition is that the selected pattern should form a spatially coherent network rather than a scattered set of isolated voxels. This means that neighbouring voxels are encouraged to have similar weights. If E denotes the set of neighbouring voxel pairs, this penalty can be written as

$$\min_{w} \sum_{(p,q)\in E} (w_p - w_q)^2. \tag{S1}$$

A spatial adjacency matrix is constructed from voxel anatomy, and its graph Laplacian is used to define the spatial smoothness penalty. This gives

$$L_{\text{smooth}}(w) = w^\top C_{\text{smooth}} w, \qquad C_{\text{smooth}} = L_{\text{graph}}. \tag{S2}$$

Thus, C_{smooth} enforces spatial smoothness across neighbouring voxels. When the optimisation is performed in PCA space, this spatial smoothness penalty is expressed in the same reduced space before optimisation.

Condition 4: The projected signal should show low consecutive-trial variability.

The fourth condition is that the selected signal should exhibit low consecutive-trial variability. Variability is therefore defined as the difference between consecutive trials, rather than as variance across the full time series. This distinction is important because variance captures the overall dispersion of a signal around its mean but does not account for trial order. As a result, two projected signals may have similar variance even if one evolves smoothly across trials while the other fluctuates sharply from one trial to the next. Consecutive-trial differences, in contrast, preserve the local temporal structure of the signal and quantify local changes between neighbouring trials.

This definition is motivated by the behavioural structure of motor vigour. Vigour on trial i is unlikely to be statistically independent from vigour on trial $i + 1$, because consecutive trials are shaped by similar physiological, attentional, and task-related states. Trials that occur close in time are therefore expected to be more similar than trials separated by longer intervals. Accordingly, a stable vigour-related neural signal would be expected to show fewer large unexplained jumps between consecutive trials. This reasoning is consistent with previous work on reaction-time variability showing that standard deviation alone does not capture temporal dependency, whereas measures such as lag-one autocorrelation and successive-trial differences reveal the local structure of performance fluctuations (Perquin et al., 2023).

A similar principle has been applied in fMRI research using mean squared successive difference (MSSD) and related measures, which quantify moment-to-moment changes in BOLD activity rather than dispersion around a global mean (Garrett et al., 2011; Wehrheim et al., 2024; Nomi et al., 2018). This approach is particularly appropriate for task fMRI, where the expected signal may change gradually over time, across task conditions, or between blocks. A global variance measure could incorrectly treat such slow task-related changes as variability, whereas a consecutive-difference penalty specifically targets local instability between consecutive trials. Thus, the low-variability term in the present model penalises large consecutive-trial changes in the projected signal while allowing slower task-related changes to remain. Boundaries between runs and blocks are excluded from this penalty because trials across these boundaries do not reflect continuous local temporal dynamics and may contain artificial discontinuities introduced by changes in run structure or task context.

The objective therefore minimises variability between consecutive trials:

$$\begin{aligned}
\min_{w} \sum_{i\in I_c} \left\| y_{\text{bold},i} - y_{\text{bold},i+1} \right\|_2^2 &= \min_{w} \sum_{i\in I_c} \left(y_{\text{bold},i} - y_{\text{bold},i+1} \right) \left(y_{\text{bold},i} - y_{\text{bold},i+1} \right)^\top \\
&= \min_{w} \sum_{i\in I_c} \left(w^\top X_{\text{bold},i} - w^\top X_{\text{bold},i+1} \right) \left(w^\top X_{\text{bold},i} - w^\top X_{\text{bold},i+1} \right)^\top \\
&= \min_{w} \sum_{i\in I_c} w^\top \left(X_{\text{bold},i} - X_{\text{bold},i+1} \right) \left(X_{\text{bold},i} - X_{\text{bold},i+1} \right)^\top w. \qquad \text{(S3)}
\end{aligned}$$

Here, I_c denotes the set of valid consecutive-trial pairs after excluding run and block boundaries, and $y_{\text{bold},i} \in R^{1\times time\_points}$ is the window-BOLD signal in a trial. This gives the BOLD variability loss:

$$
\begin{aligned}
L_{\text{bold}}(w) &= w^\top C_{\text{bold}} w, \\
C_{\text{bold}} &= \sum_{i \in I_c} \left(X_{\text{bold},i} - X_{\text{bold},i+1}\right) \left(X_{\text{bold},i} - X_{\text{bold},i+1}\right)^\top.
\end{aligned} \tag{S4}
$$

Therefore, C_{bold} penalises large changes in BOLD activity between consecutive trials.

The same low-variability condition is also applied to the beta estimates:

$$
\begin{aligned}
\min_w \sum_{i \in I_c} \left\|y_{\beta,i} - y_{\beta,i+1}\right\|_2^2 &= \min_w \sum_{i \in I_c} \left(y_{\beta,i} - y_{\beta,i+1}\right) \left(y_{\beta,i} - y_{\beta,i+1}\right)^\top \\
&= \min_w \sum_{i \in I_c} \left(w^\top X_{\beta,i} - w^\top X_{\beta,i+1}\right) \left(w^\top X_{\beta,i} - w^\top X_{\beta,i+1}\right)^\top \\
&= \min_w \sum_{i \in I_c} w^\top \left(X_{\beta,i} - X_{\beta,i+1}\right) \left(X_{\beta,i} - X_{\beta,i+1}\right)^\top w.
\end{aligned} \tag{S5}
$$

This gives the beta variability loss:

$$
\begin{aligned}
L_\beta(w) &= w^\top C_\beta w, \\
C_\beta &= \sum_{i \in I_c} \left(X_{\beta,i} - X_{\beta,i+1}\right) \left(X_{\beta,i} - X_{\beta,i+1}\right)^\top.
\end{aligned} \tag{S6}
$$

Therefore, C_β penalises large changes in beta values between consecutive trials.

Both BOLD and beta variability terms are included because they capture complementary sources of variability. The beta estimates reflect activation related to movement execution, so $L_\beta(w)$ constrains variability in the movement-related neural response. However, the task also includes other processes. These sources of variability can be encoded in the BOLD signal even when they are not fully captured by movement-related beta estimates. Therefore, $L_{\text{bold}}(w)$ is also included to account for broader task-related variability encoded in the brain.

General loss function: After defining the four conditions, the penalty terms are combined into a single total loss. The total penalty is defined as

$$
L_{\text{total}}(w) = \alpha_{\text{task}} L_{\text{task}}(w) + \alpha_{\text{BOLD}} L_{\text{bold}}(w) + \alpha_\beta L_\beta(w) + \alpha_{\text{smooth}} L_{\text{smooth}}(w) = w^\top C_{\text{total}} w,
$$

where

$$
C_{\text{total}} = \alpha_{\text{task}} C_{\text{task}} + \alpha_{\text{BOLD}} C_{\text{bold}} + \alpha_\beta C_\beta + \alpha_{\text{smooth}} C_{\text{smooth}}. \tag{S7}
$$

The parameters α_{task}, α_{BOLD}, α_β, and α_{smooth} control the relative contributions of task engagement, BOLD variability, beta variability, and spatial smoothness, respectively.

The final objective combines this total penalty with behavioural relevance. The model minimises the regularisation penalties while maximising the association between the projected beta signal and reaction time:

$$
\min_w \frac{\gamma L_{total}(w) - L_{RT}(w)}{L_{var}(w)} = \frac{\gamma w^\top C_{\text{total}} w - w^\top C_b w}{w^\top C_d w} \quad \text{s.t. } \|w\|_2 = 1. \tag{S8}
$$

Here, γ controls the balance between satisfying the regularisation criteria and maximising behavioural relevance. The denominator $w^\top C_d w$ normalises the objective by the variance of the projected beta signal.

The resulting projection is defined as

$$y = w^\top X_\beta. \tag{S9}$$

The optimisation problem was solved under the unit-norm constraint on w using the Sequential Least Squares Programming (SLSQP) algorithm (Nocedal and Wright, 2006; Kraft, 1988). The objective combines a behavioural relevance term with four regularisation terms. The coefficients α_{task}, α_{BOLD}, α_β, and α_{smooth} control the relative contribution of the task-engagement, BOLD consecutive-trial variability, beta consecutive-trial variability, and spatial smoothness penalties, respectively. The scalar γ controls the overall strength of the combined penalty terms relative to the behavioural relevance term. Hyperparameters were selected using a coarse-to-fine cross-validation procedure. In the coarse stage, the model was evaluated over a predefined set of candidate penalty-weight combinations chosen to span weaker, balanced, and stronger weighting of the different penalties. Trials were divided into contiguous folds, and for each candidate setting the model was fit on the training folds and evaluated on the held-out fold using the absolute correlation between the optimised neural projection and behaviour. After the best-performing coarse setting was identified, a fine search was performed by testing small multiplicative perturbations around that setting, both for individual penalty weights and for the overall balance of all penalty weights. The final hyperparameter setting was chosen based on held-out correlation, with preference given to settings that showed stable performance across folds and small train-test differences. For each fold, the optimisation was then solved independently using the training data, and the resulting weights were projected back from PCA space to voxel space. Final voxel maps and projected signals were obtained by averaging across folds. The resulting weight assigned to each voxel reflected its contribution to satisfying the combined criteria of task engagement, behavioural relevance, spatial smoothness, and low variability. Voxels with the largest weights then define the vigour network.

Finally, we define the vigour network as voxels whose weights exceed the 90th percentile. We choose this threshold because the 90th percentile provides a conservative but stable compromise between spatial specificity and anatomical coverage (Supplementary Figure S6). Lower thresholds, such as the 80th or 85th percentiles, retain substantially more voxels and therefore produce a broader, less selective network. In contrast, more stringent thresholds, particularly the 95th and 97.5th percentiles, sharply reduce the number of retained voxels and begin to exclude several anatomical regions. The p90 threshold preserves the main anatomical groups while avoiding the broader p80/p85 maps and the regional loss seen at p95/p97.5. It is an appropriate cutoff for defining the vigour network.

7 MEDICATION-STATE CONTROL ANALYSES

7.1 Control analysis: medication-related connectivity-pattern changes exceeded run-to-run variation

To test whether medication-related changes in the vigour-network connectivity pattern were larger than run-to-run variation, we compared within-state run 1-run 2 distances with OFF-ON medication distances for each participant. OFF-ON distances were larger than within-state run-to-run distances for both Pearson-correlation and mutual-information patterns (Pearson: $d = 1.15$, $p = 0.0006$; mutual information: $d = 0.69$, $p = 0.0015$; Figure S9). This suggests that the network connectivity pattern changed with dopaminergic medication more than would be expected from short-term measurement fluctuation alone.

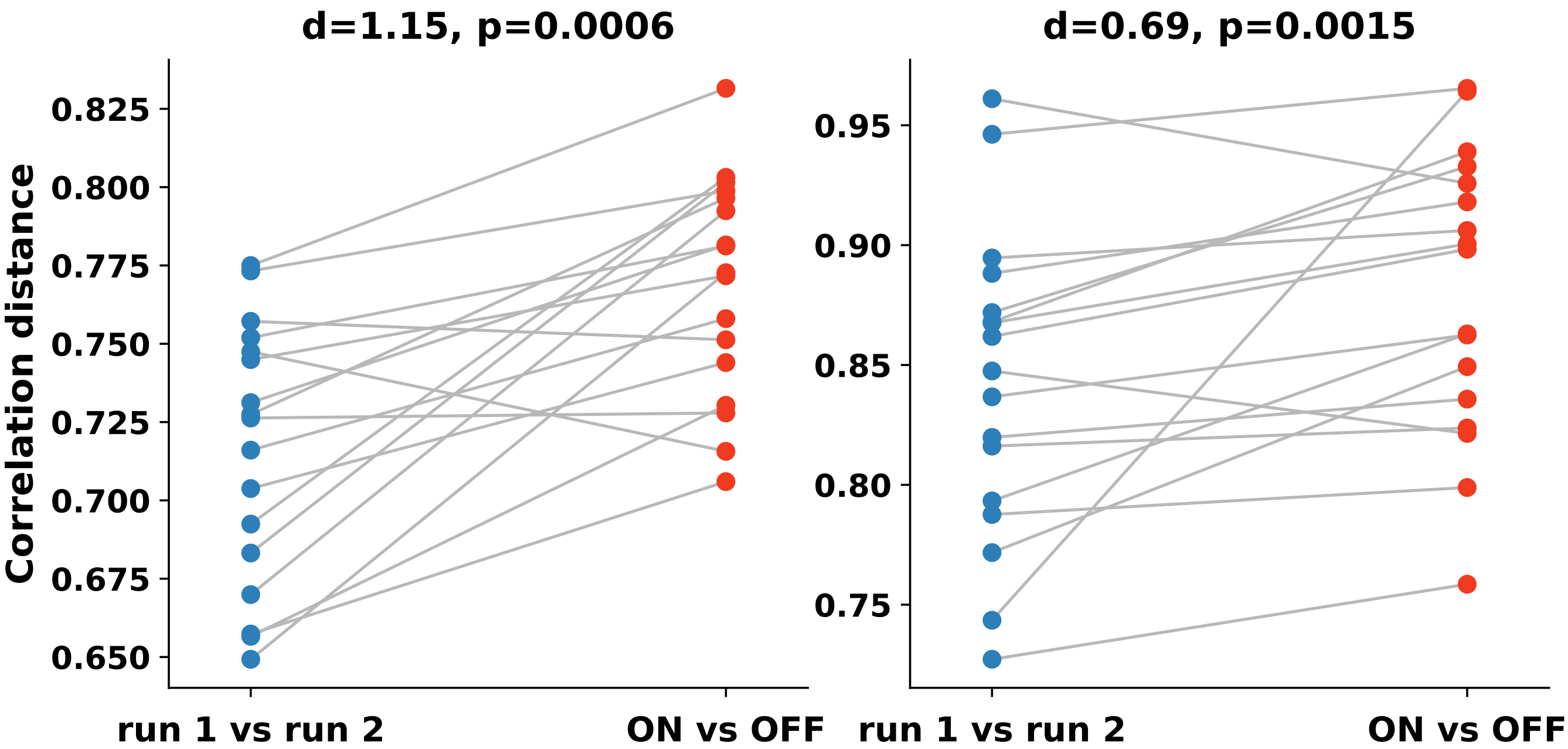

Figure S9: Medication-state control analysis for connectivity-pattern stability. Subject-level paired correlation-distance plots compare within-state run 1 versus run 2 distances with ON- versus OFF-medication distances. Lines connect paired estimates from the same participant. **Left**, Pearson-based consistency; **Right**, mutual-information-based consistency. Separate panels show the paired distances for the displayed connectivity patterns.

7.2 Control analysis: medication-related FC changes relative to run-to-run baseline variability

To test whether the medication-related within-versus-between-ROI FC pattern reported in main Figure 7 exceeded ordinary short-term variability, we repeated the same subject-level FC-change analysis using within-session run-to-run differences as a baseline and compared these values with the between-session medication-effect estimate (Figure S10). In the vigour network, medication-related between-session change was larger than run-to-run variability for intra-ROI FC ($p = 0.005$) and for the intra-minus-between contrast ($p = 0.022$), but not for between-ROI FC ($p = 0.961$). In the task-activation map, between-session change was larger than run-to-run variability for intra-ROI FC ($p = 0.011$) and between-ROI FC ($p = 0.015$), with weaker evidence for the intra-minus-between contrast ($p = 0.059$). Thus, the baseline analysis supports medication-related FC changes beyond short-term measurement fluctuation, with the local-versus-distributed specificity clearest in the vigour network.

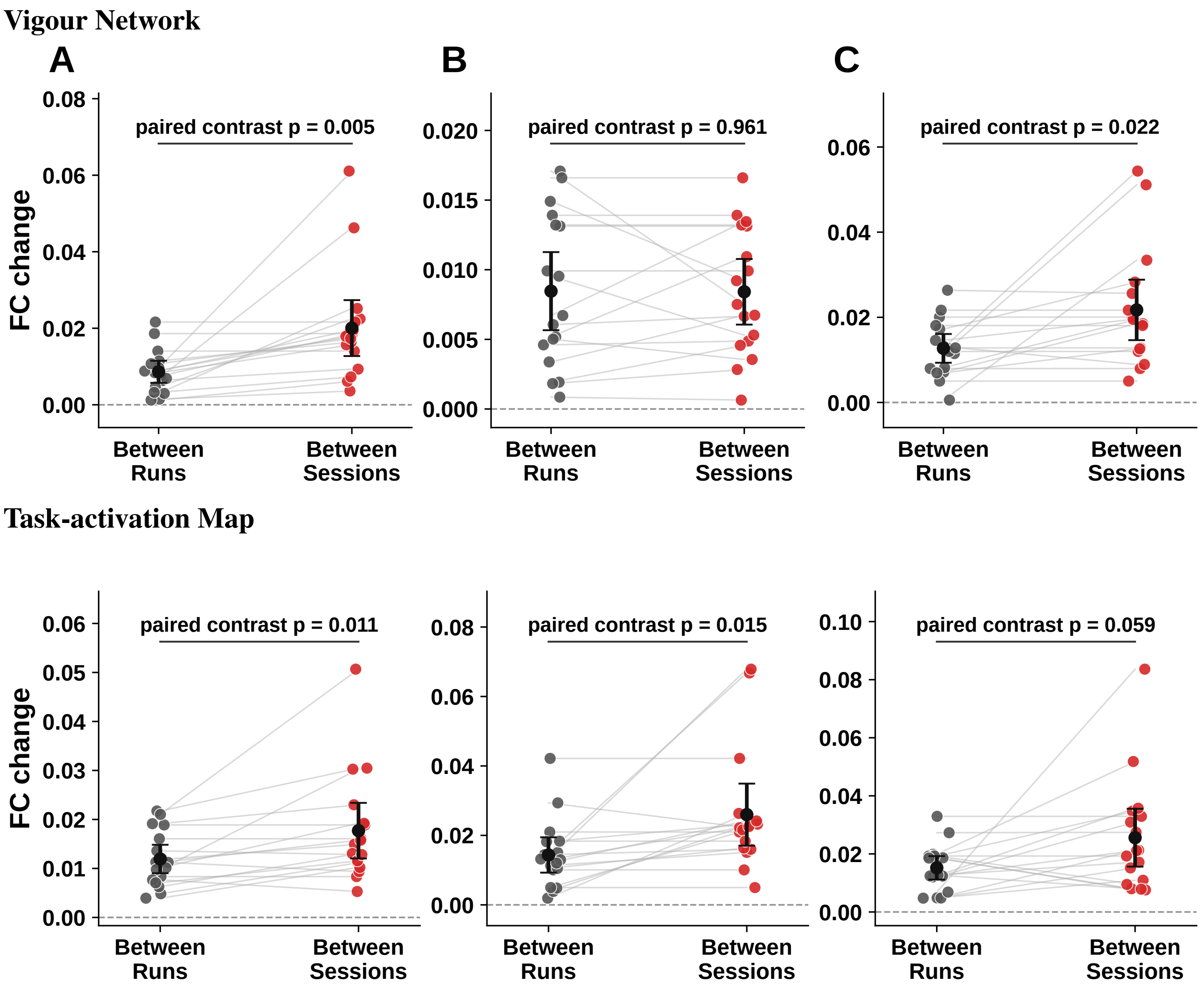

Figure S10: Run-to-run baseline control for the medication-related functional-connectivity effect. The top row shows the vigour network, and the bottom row shows the task-activation map. Within each row, panels show **A**, intra-ROI FC; **B**, between-ROI FC; and **C**, the intra-minus-between FC contrast. Grey and red points show individual participants, paired lines connect matched run-to-run and medication-effect estimates, and black markers summarise the group means with uncertainty.

We also tested whether head motion differed between sessions or accounted for the vigour-network FC result. Mean Power framewise displacement (FD) was calculated for each run and averaged within participant and session. Mean FD was 0.236 mm OFF medication and 0.264 mm ON medication; the paired ON–OFF difference was 0.028 mm (95% CI $[-0.0032, 0.0588]$ mm; $t(17) = 1.892$, $p = 0.076$; Figure S11). Linear mixed-effects models included session-level mean FD, separated into within- and between-participant components. After adjustment, the ON-OFF within-ROI difference remained significant ($\beta = 0.01594$ Fisher-z, 95% CI $[0.00590, 0.02597]$, $p = 0.0019$), whereas the between-ROI difference remained non-significant ($\beta = -0.00127$ Fisher-z, 95% CI $[-0.00515, 0.00262]$, $p = 0.523$). Excluding the participant with ON-session mean FD above 0.5 mm gave the same pattern: the within-ROI difference remained significant ($p = 0.0101$), whereas the between-ROI difference remained non-significant ($p = 0.594$).

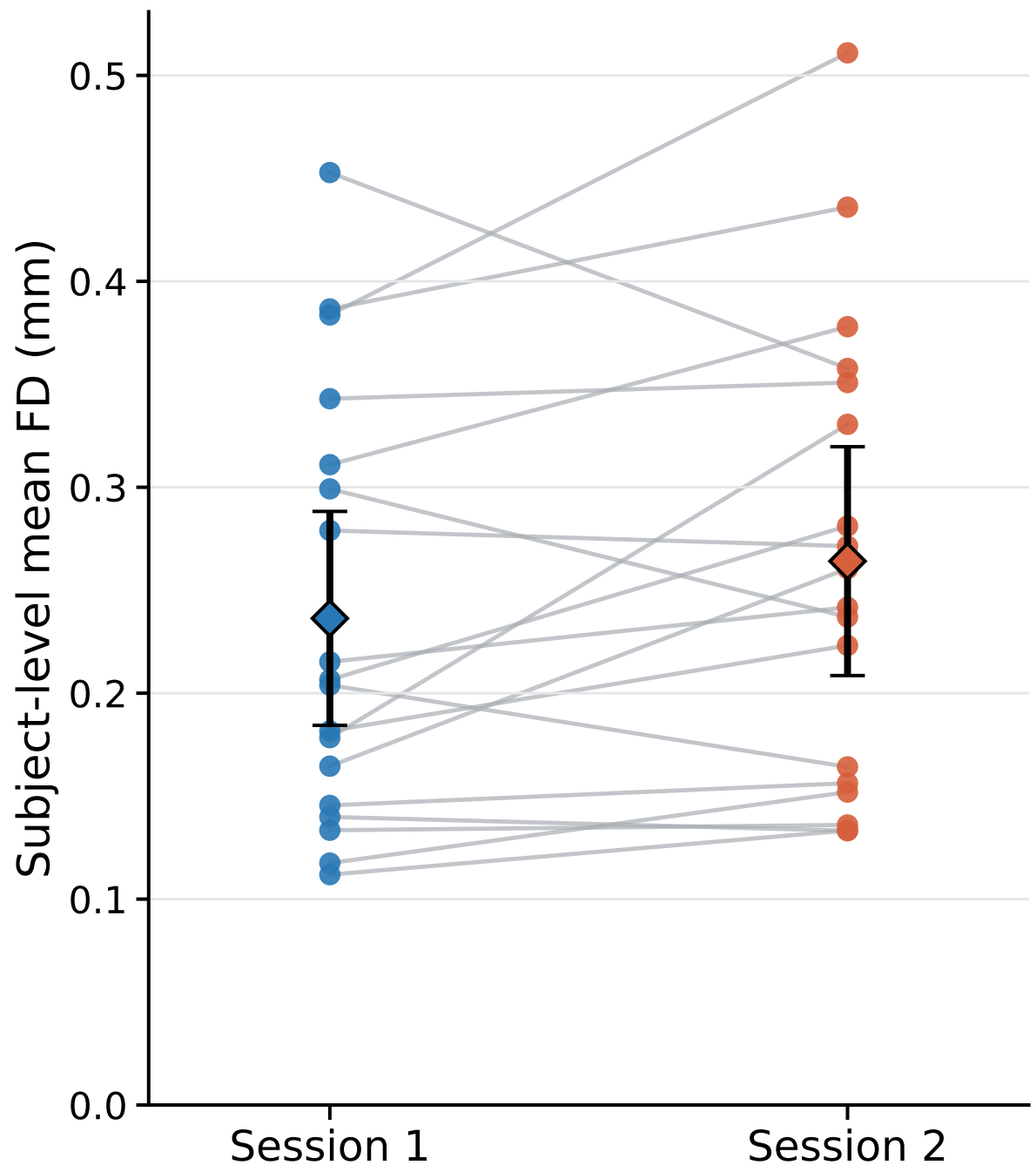

Figure S11: Participant-level mean framewise displacement (FD) in Session 1 (OFF medication) and Session 2 (ON medication). Grey lines connect paired values from the same participant. Coloured circles show individual values; diamonds and error bars show the group means and 95 percent confidence intervals.

8 GVS-RELATED SUPPLEMENTARY ANALYSES

This section provides supporting analyses for the GVS results.

8.1 Projected signal time courses

Figure S12 shows the averaged projected time courses for sham and GVS1-GVS8. The vigour-network projection shows stimulus- and medication-dependent differences in the shape of the signal. This descriptive plot provides the time-course context for the feature-based tests reported below. The task-activation map projection is included as a reference to test whether similar waveform changes are also present in task-active circuitry.

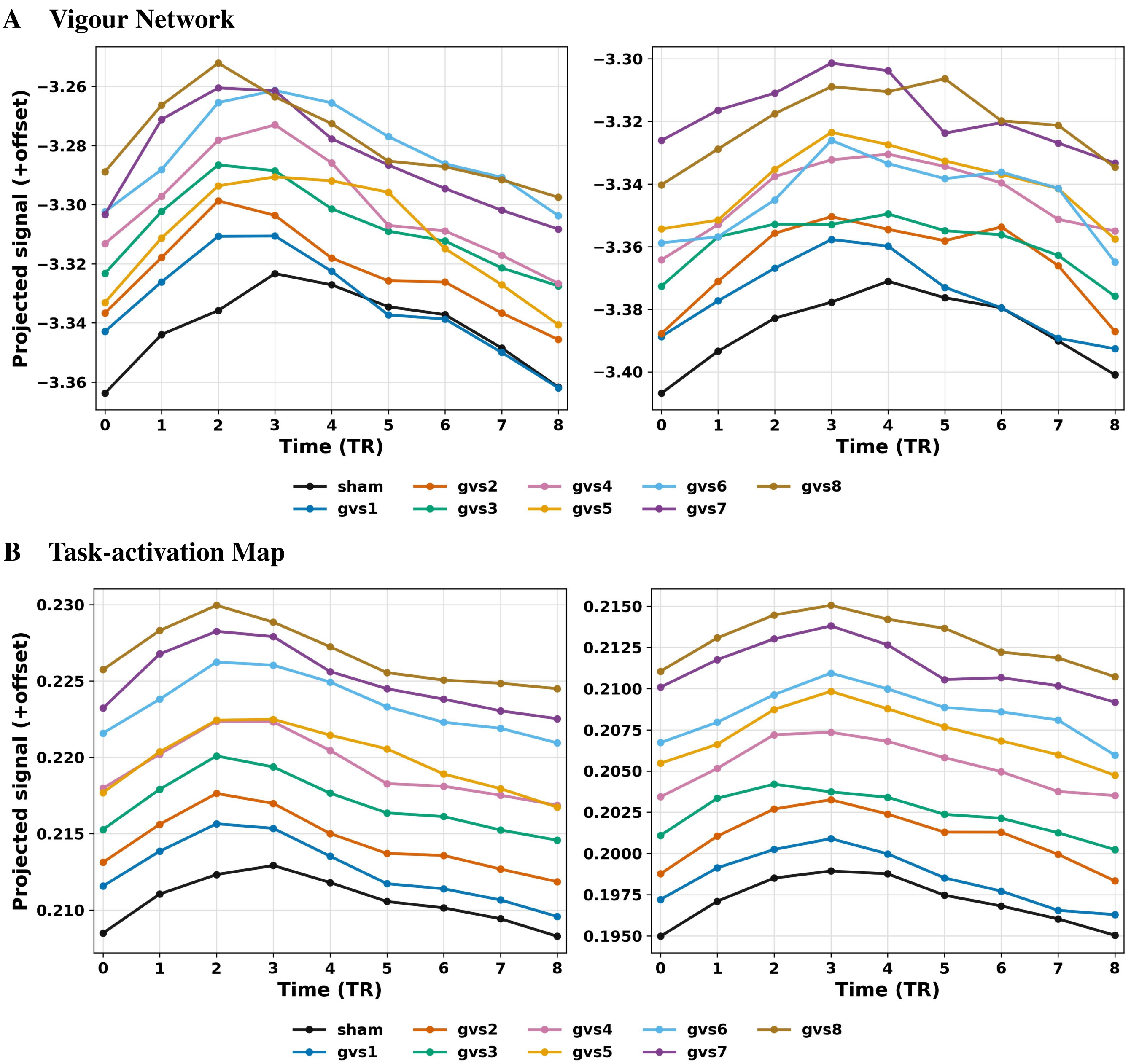

Figure S12: Projected time courses by GVS condition. **A**, Vigour-network projected signal in the OFF- and ON-medication sessions. **B**, Task-activation map projected signal in the OFF- and ON-medication sessions. Lines show condition means for sham and active GVS waveforms. Vertical offsets are used only for visual separation.

8.2 Response-feature summaries

Figure S13 summarises the four predefined active-minus-sham response features for the vigour-network and task-activation map projections: late-minus-early response, linear slope, maximum absolute baseline response, and peak-to-peak amplitude. In the vigour network, GVS7 shows the corrected change in the late-minus-early response feature in the OFF-medication session, with a standardised paired effect size of $d_z = -0.94$. This matches the main behavioural result, where GVS7 is the only waveform associated with faster responses OFF medication. No vigour-network feature survived correction in the ON-medication session. This pattern supports the interpretation that the behaviourally relevant GVS waveform is also reflected in the vigour-network projection.

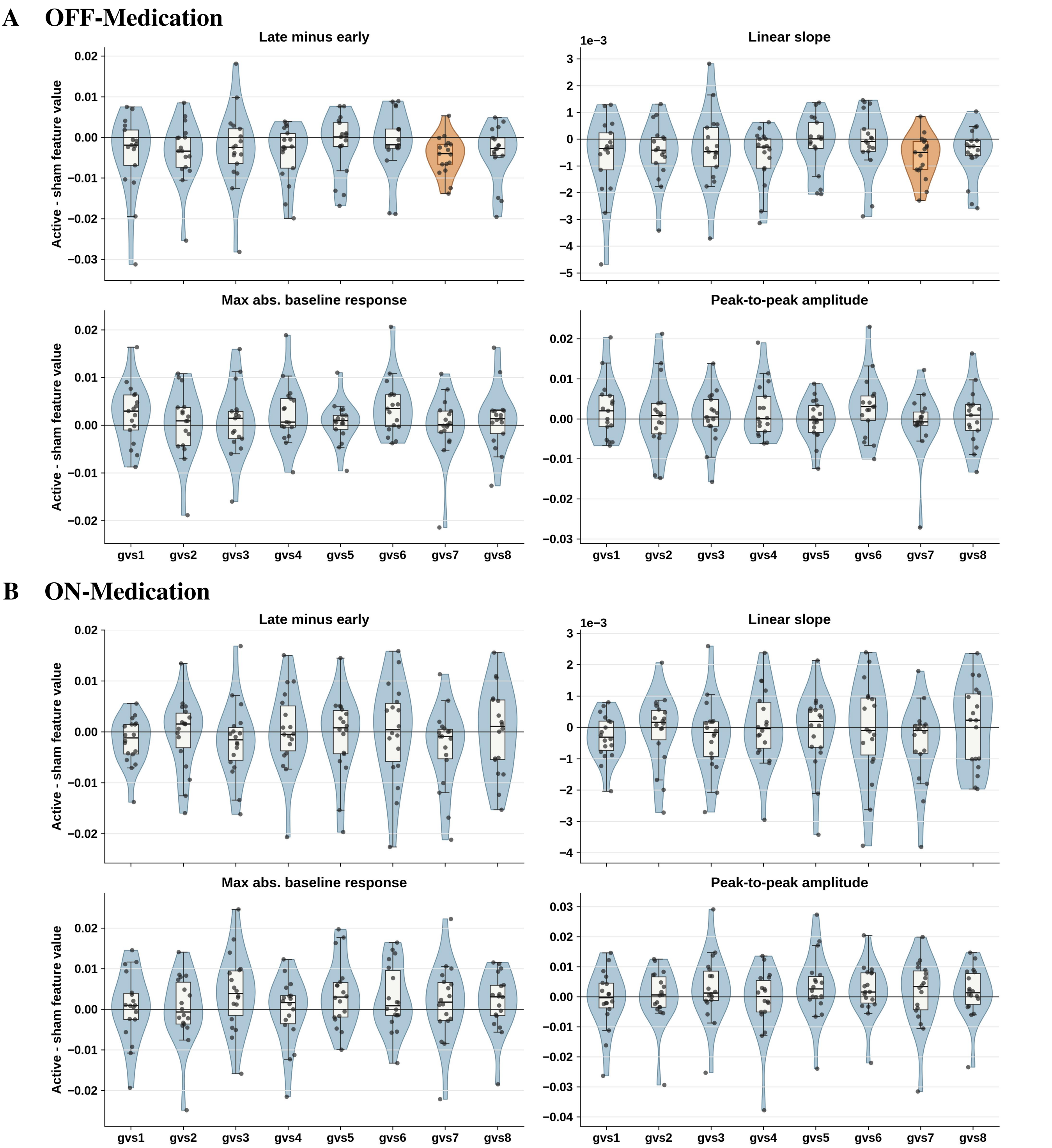

Figure S13: GVS-related response-feature changes in the vigour network. **A**, OFF-medication session. **B**, ON-medication session. Violin plots show active-minus-sham feature values for the late-minus-early response, linear slope, maximum absolute baseline response, and peak-to-peak amplitude features. Highlighted distributions mark stimulation conditions that survived FDR correction within the displayed analysis.

Figure S14 applies the same response-feature analysis to the task-activation map projection. This control analysis does not reproduce the GVS7-specific pattern observed in the vigour network. During the OFF-medication session, FDR-significant task-activation-map effects were observed for GVS2 for the late-early

response ($d_z = -0.86$) and linear slope ($d_z = -0.84$); GVS6 for the maximum absolute baseline response ($d_z = 0.76$); and GVS8 for the late-early response ($d_z = -0.92$) and linear slope ($d_z = -0.86$). No comparison survived FDR correction during the ON-medication session. These results suggest that the vigour-network projection captures the behaviourally relevant GVS effect more specifically than the task-activation map.

A OFF-Medication

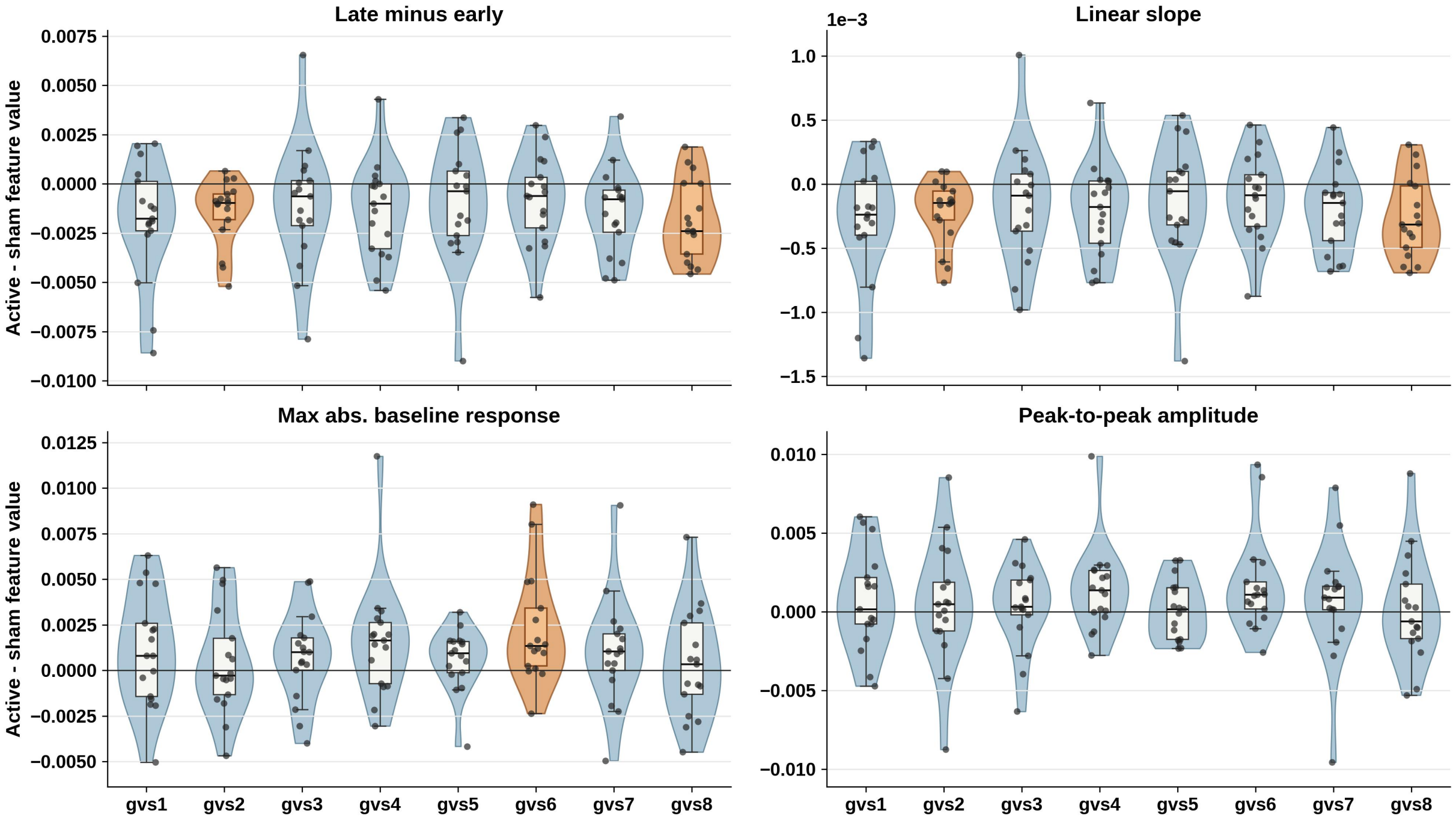

B ON-Medication

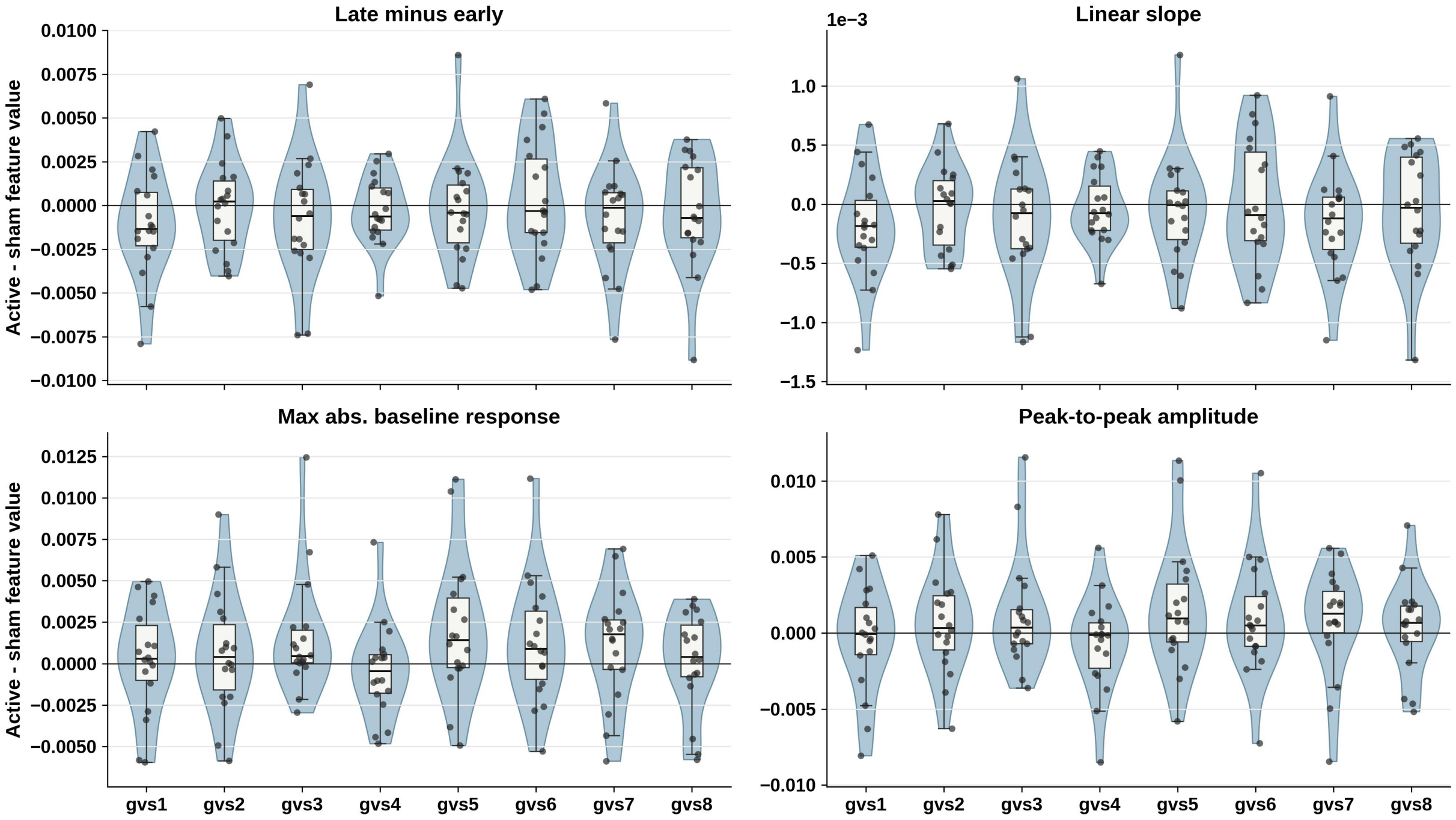

Figure S14: GVS-related response-feature changes in the task-activation map projection. **A**, OFF-medication session. **B**, ON-medication session. Violin plots show active-minus-sham feature values for the late-minus-early response, linear slope, maximum absolute baseline response, and peak-to-peak amplitude features. Highlighted distributions mark stimulation conditions that survived FDR correction within the displayed analysis.

8.3 Joint analysis of network effects

To compare GVS effects between the vigour network and task-activation map directly, we fitted a linear mixed-effects model for each temporal feature that included both networks. The model was

$$Y_{ij} = \beta_0 + \text{stimulation}_{ij} + \text{network}_{ij} + \left(\text{stimulation} \times \text{network}\right)_{ij} + \text{medication}_{ij} + b_i + \varepsilon_{ij}. \quad \text{(S10)}$$

Stimulation condition, network, their interaction, and medication state were fixed effects, and b_i was a participant-specific random intercept. Stimulation was categorical, with sham as the reference condition. Medication was additive, so stimulation effects were estimated across medication states. We estimated model-based contrasts comparing GVS1-GVS8 with sham separately within each network, giving 16 contrasts per feature. Benjamini-Hochberg FDR correction was applied across all 16 contrasts within each feature, with significance defined as $q < 0.05$.

The joint model identified corrected effects only for GVS7 in the vigour network. The GVS7-sham difference was -0.00361 for the early-late change (95% CI $[-0.00579, -0.00143]$, $p = 0.00117$, $q = 0.0187$) and -0.000562 for slope (95% CI $[-0.000915, -0.000208]$, $p = 0.00183$, $q = 0.0293$). No other contrast survived the same correction, including GVS7 in the task-activation map. Thus, when both networks were evaluated under the same model and multiplicity correction, the GVS7 temporal effect was localised to the vigour network (Figure S15).

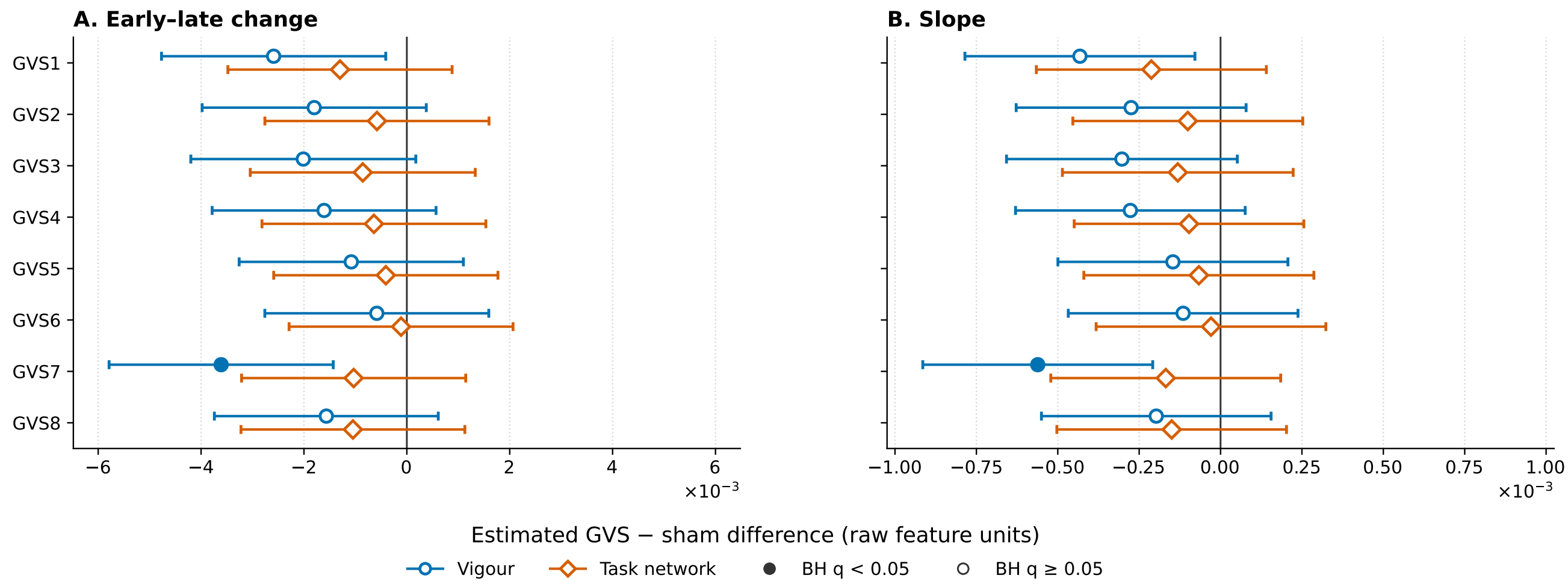

Figure S15: Joint-network model-based GVS-versus-sham contrasts for the early-late change and slope in the vigour network and task-activation map. Error bars show 95% confidence intervals. Filled markers denote contrasts surviving Benjamini-Hochberg FDR correction across all 16 contrasts within the corresponding feature ($q < 0.05$).

8.4 Connectivity analyses

Each stimulation condition contributed only 10 trials per run, limiting the stability of block-wise connectivity estimates. The analysis also pooled repeated block-level observations across participants, medication states, runs, and waveforms; accordingly, the following findings are exploratory and describe network perturbability rather than a waveform-specific or causal therapeutic effect. Connectivity was evaluated across 38 ROIs, corresponding to 703 unique undirected edges. Using mutual information, active GVS produced 156 sham-referenced edge changes that were significant after FDR correction among 703 tested edges. Of these, 46 increased and 110 decreased (Figure S16A). The strongest decreases are observed in the left amygdala-left thalamus, left cerebellum-left putamen, and left cingulate-left thalamus. The strongest increases are observed in the left parietal cortex-left thalamus and the left

putamen-left supplementary motor area. At the anatomical-sector level, decreases are most concentrated between limbic/olfactory and somatomotor/cerebellar regions, with additional decreases linking subcortical, cingulate-temporal, and somatomotor/cerebellar sectors. As a control, we repeated the mutual-information GVS edge-change analysis using the task-activation map (Figure S16B). GVS also produced significant changes in this task-activation network, but the pattern differed: the task-activation map shows 134 significant mutual-information edges, with only 14 mutual-information edges overlapping those in the vigour network.

A Vigour Network

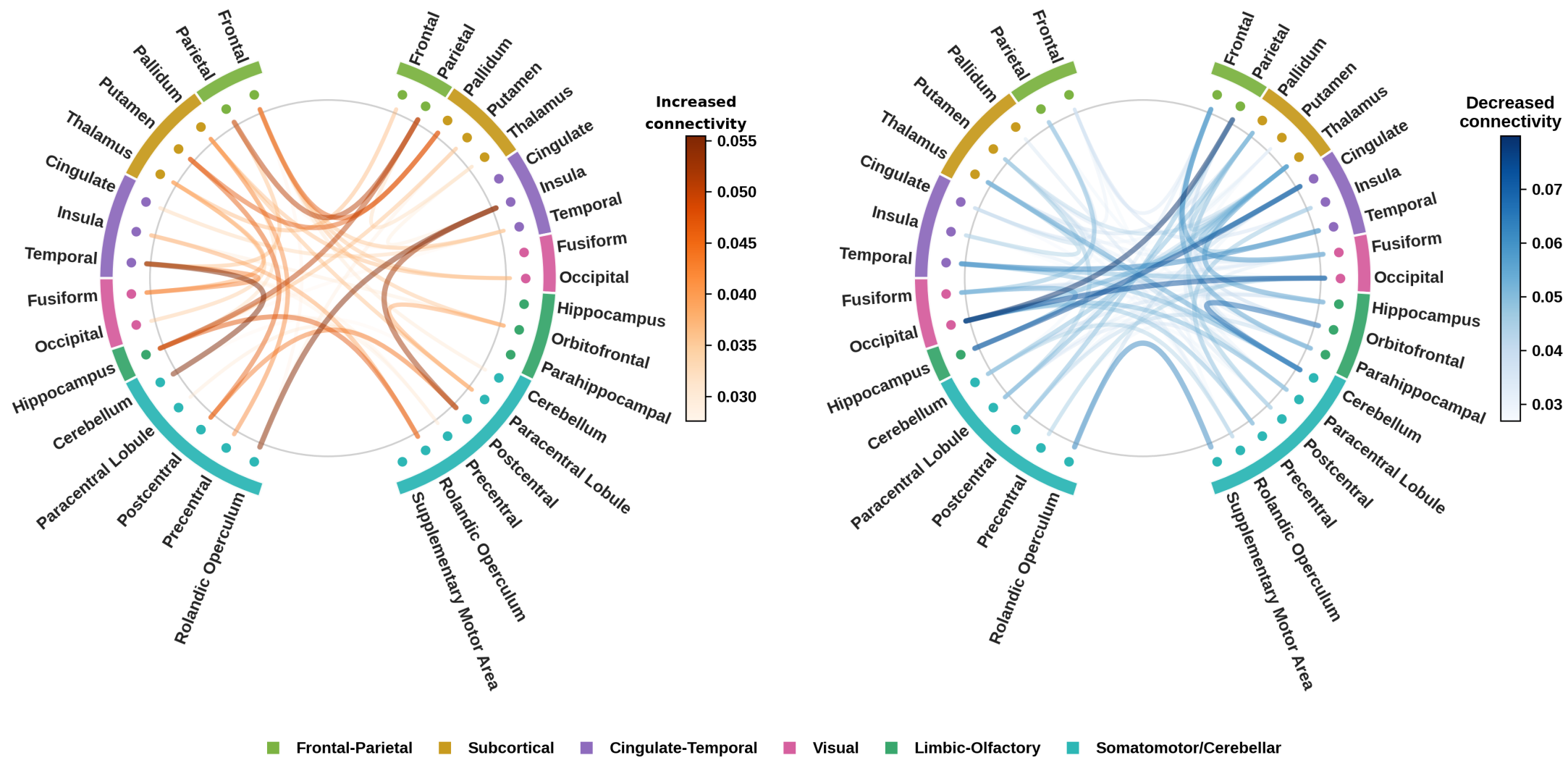

B Task-activation Map

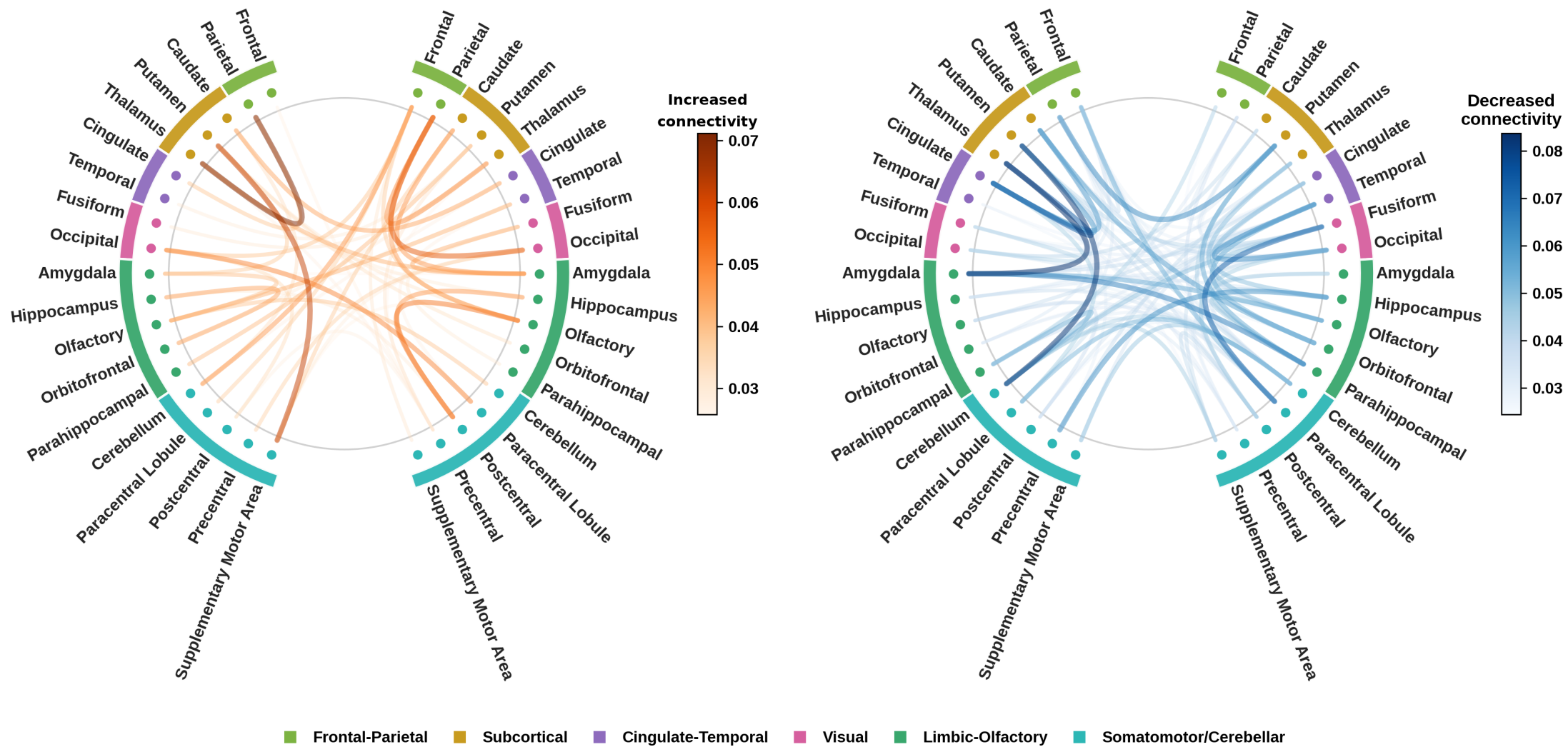

Figure S16: Exploratory GVS-related mutual-information edge changes in the vigour network and task-activation map. **A**, mutual-information analysis for the vigour network. **B**, for the task-activation map. Each panel shows active-sham edge changes that were significant after FDR correction, separately for increased and decreased connectivity. Changes were pooled across subjects, medication states, runs, and active GVS waveforms. Orange edges mark increased statistical dependence, and blue edges mark decreased statistical dependence; colour intensity scales with the mean edge-change magnitude. Nodes are labelled by ROI and grouped by anatomical sector.

Using Pearson correlation, active GVS produced 194 significant edges (106 increases, 88 decreases; Figure S17A). The strongest increases involve left amygdala-left paracentral lobule, right postcentral cortex-left temporal cortex, and left cerebellum-right paracentral lobule. The strongest decreases are observed in the left caudate-right frontal cortex, right amygdala-left precentral cortex, and left thalamus-left

temporal cortex. As a control, we repeated the Pearson-correlation GVS edge-change analysis using the task-activation map (Figure S17B). GVS also produced significant changes in this task-activation network, but the pattern differed: the task-activation map shows 148 significant Pearson edges, with only 16 Pearson edges overlapping those in the vigour network. The two metrics do not identify the same set of edges, consistent with the different dependencies captured by the two metrics. Although the two metrics identify different edges, both show distributed GVS-related changes involving limbic/olfactory, medial-temporal, subcortical, visual, cingulate-temporal, and somatomotor/cerebellar components.

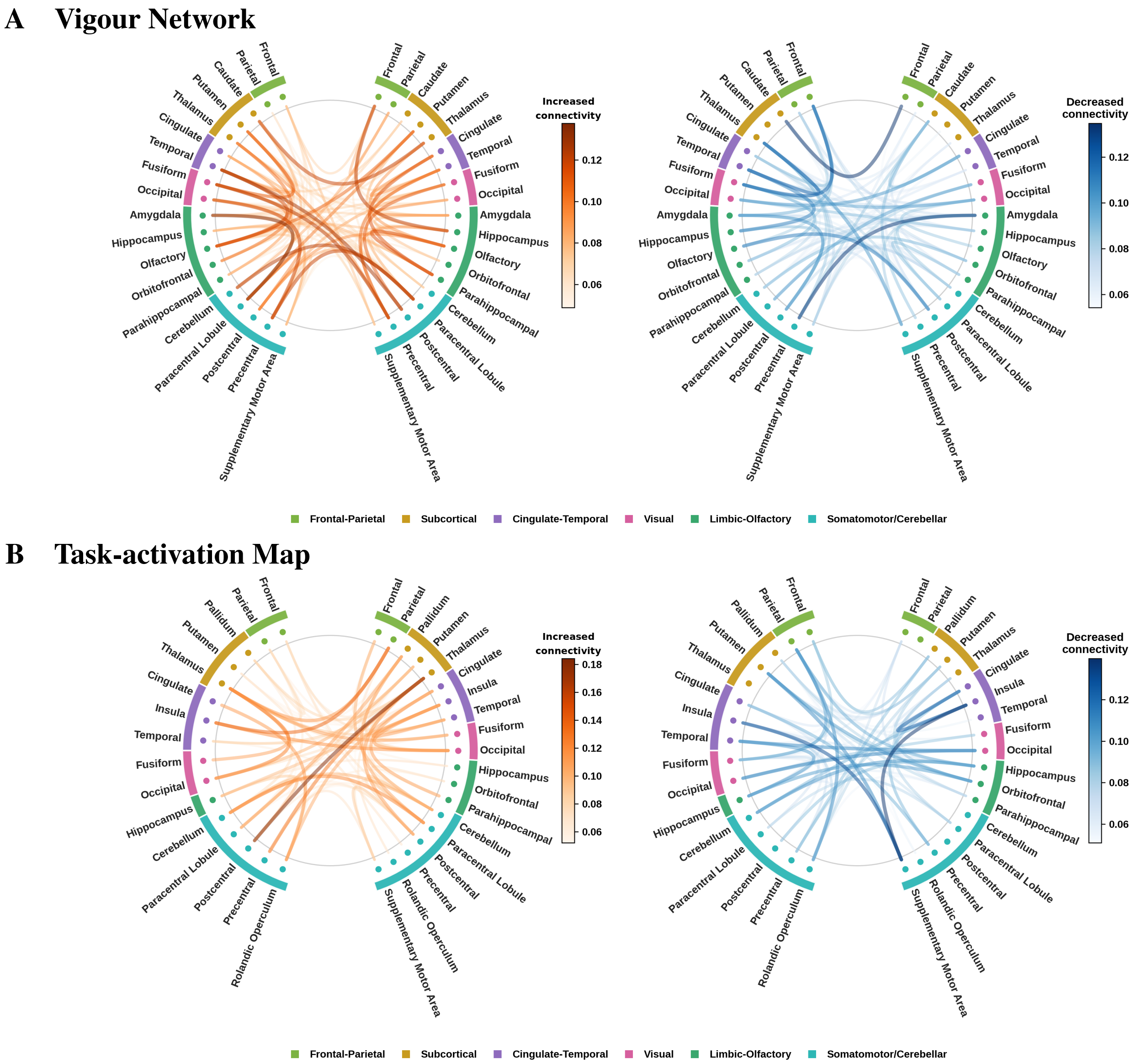

Figure S17: GVS-related Pearson correlation edge changes in the vigour network and task-activation map. **A**, Pearson correlation analysis for the vigour network. **B**, for the task-activation map. Each panel shows significant FDR-corrected active-sham edge changes. Orange edges mark increased Pearson correlation, and blue edges mark decreased Pearson correlation; colour intensity scales with the mean edge-change magnitude. Nodes are labelled by ROI and grouped by anatomical sector.

Together, these supplementary analyses support the main conclusion that GVS effects are not uniform across waveforms, medication states, response features, or network definitions. The vigour network appears to capture the GVS waveform linked to faster OFF-medication responses, whereas broader connectivity changes are present in both the vigour network and the task-activation map. This supports a cautious interpretation: GVS influences task-active circuitry broadly, but the vigour network shows a partly distinct pattern of stimulation-sensitive connectivity.

REFERENCES

Beckmann, C. F., Jenkinson, M., and Smith, S. M. (2003). General multilevel linear modeling for group analysis in fMRI. *Neuroimage* 20, 1052–1063

Garrett, D. D., Kovacevic, N., McIntosh, A. R., and Grady, C. L. (2011). The importance of being variable. *Journal of Neuroscience* 31, 4496–4503

Jenkinson, M., Beckmann, C. F., Behrens, T. E., Woolrich, M. W., and Smith, S. M. (2012). FSL. *Neuroimage* 62, 782–790

Kay, K., Rokem, A., Winawer, J., Dougherty, R., and Wandell, B. (2013). GLMdenoise: a fast, automated technique for denoising task-based fMRI data. *Frontiers in Neuroscience* 7, 54426

Kraft, D. (1988). A software package for sequential quadratic programming. *Forschungsbericht- Deutsche Forschungs- und Versuchsanstalt fur Luft- und Raumfahrt*

Nguyen, T. T., Kang, J.-J., and Oh, S.-Y. (2022). Thresholds for vestibular and cutaneous perception and oculomotor response induced by galvanic vestibular stimulation. *Frontiers in Neurology* 13, 955088

Nocedal, J. and Wright, S. J. (2006). *Numerical optimization* (Springer)

Nomi, J. S., Schettini, E., Voorhies, W., Bolt, T. S., Heller, A. S., and Uddin, L. Q. (2018). Resting-state brain signal variability in prefrontal cortex is associated with ADHD symptom severity in children. *Frontiers in Human Neuroscience* 12, 90

Perquin, M. N., van Vugt, M. K., Hedge, C., and Bompas, A. (2023). Temporal structure in sensorimotor variability: a stable trait, but what for? *Computational Brain & Behavior* 6, 400–437

Prince, J. S., Charest, I., Kurzawski, J. W., Pyles, J. A., Tarr, M. J., and Kay, K. N. (2022). Improving the accuracy of single-trial fMRI response estimates using GLMsingle. *elife* 11, e77599

Pruim, R. H., Mennes, M., van Rooij, D., Llera, A., Buitelaar, J. K., and Beckmann, C. F. (2015). ICA-AROMA: A robust ICA-based strategy for removing motion artifacts from fMRI data. *Neuroimage* 112, 267–277

Rolls, E. T., Huang, C.-C., Lin, C.-P., Feng, J., and Joliot, M. (2020). Automated anatomical labelling atlas 3. *Neuroimage* 206, 116189

Roweis, S. (1997). EM algorithms for PCA and SPCA. *Advances in neural information processing systems* 10

Smith, S. M., Jenkinson, M., Woolrich, M. W., Beckmann, C. F., Behrens, T. E., Johansen-Berg, H., et al. (2004). Advances in functional and structural MR image analysis and implementation as FSL. *Neuroimage* 23, S208–S219

Wehrheim, M. H., Faskowitz, J., Schubert, A.-L., and Fiebach, C. J. (2024). *Reliability of variability and complexity measures for task and task-free BOLD fMRI*. Tech. rep., Wiley Online Library

Woolrich, M. W., Behrens, T. E., Beckmann, C. F., Jenkinson, M., and Smith, S. M. (2004). Multilevel linear modelling for fMRI group analysis using bayesian inference. *Neuroimage* 21, 1732–1747

Worsley, K. J. (2001). Statistical analysis of activation images. In *Functional Magnetic Resonance Imaging: An Introduction to Methods*, eds. P. Jezzard, P. M. Matthews, and S. M. Smith (New York, NY: Oxford University Press), chap. 14. 251–270. doi:10.1093/acprof:oso/9780192630711.003.0014
